# Inflammatory profile of patients with tuberculosis with or without HIV-1 co-infection: a prospective cohort study and immunological network analysis

**DOI:** 10.1016/s2666-5247(21)00037-9

**Published:** 2021-05-18

**Authors:** Elsa Du Bruyn, Kiyoshi F Fukutani, Neesha Rockwood, Charlotte Schutz, Graeme Meintjes, María B Arriaga, Juan M Cubillos-Angulo, Rafael Tibúrcio, Alan Sher, Catherine Riou, Katalin A Wilkinson, Bruno B Andrade, Robert J Wilkinson

**Affiliations:** Wellcome Centre for Infectious Disease Research in Africa, Institute of Infectious Disease and Molecular Medicine (E Du Bruyn MD, N Rockwood PhD, C Schutz MD, G Meintjes PhD, C Riou PhD, K A Wilkinson PhD, B B Andrade MD, Prof R J Wilkinson FMedSci) and Department of Medicine (E Du Bruyn, C Schutz, G Meintjes, Prof R J Wilkinson), University of Cape Town, Observatory, South Africa; Instituto Gonçalo Moniz, Fundação Oswaldo Cruz, Salvador, Brazil (K F Fukutani PhD, M B Arriaga MSc, J M Cubillos-Angulo MSc, R Tibúrcio MSc, B B Andrade); Multinational Organization Network Sponsoring Translational and Epidemiological Research Initiative, Salvador, Brazil (K F Fukutani, M B Arriaga, J M Cubillos-Angulo, R Tibúrcio, B B Andrade); Curso de Medicina, Faculdade de Tecnologia e Ciências, Salvador, Brazil (K F Fukutani); Department of Infectious Diseases, Imperial College London, London, UK (N Rockwood, Prof R J Wilkinson); Department of Microbiology, Faculty of Medicine, University of Colombo, Colombo, Sri Lanka (N Rockwood); Laboratory of Parasitic Diseases, National Institute of Allergy and Infectious Diseases, National Institutes of Health, Bethesda, MD, USA (A Sher PhD); The Francis Crick Institute, London, UK (K A Wilkinson, Prof R J Wilkinson); Universidade Salvador, Laureate Universities, Salvador, Brazil (B B Andrade); Escola Bahiana de Medicina e Saúde Pública, Salvador, Brazil (B B Andrade); Division of Infectious Diseases, Department of Medicine, Vanderbilt University School of Medicine, Nashville, TN, USA (B B Andrade)

## Abstract

**Background:**

HIV-1 mediated dysregulation of the immune response to tuberculosis and its effect on the response to antitubercular therapy (ATT) is incompletely understood. We aimed to analyse the inflammatory profile of patients with tuberculosis with or without HIV-1 co-infection undergoing ATT, with specific focus on the effect of ART and HIV-1 viraemia in those co-infected with HIV-1.

**Methods:**

In this prospective cohort study and immunological network analysis, a panel of 38 inflammatory markers were measured in the plasma of a prospective patient cohort undergoing ATT at Khayelitsha Site B clinic, Cape Town, South Africa. We recruited patients with sputum Xpert MTB/RIF-positive rifampicin-susceptible pulmonary tuberculosis. Patients were excluded from the primary discovery cohort if they were younger than 18 years, unable to commence ATT for any reason, pregnant, had unknown HIV-1 status, were unable to consent to study participation, were unable to provide baseline sputum samples, had more than three doses of ATT, or were being re-treated for tuberculosis within 6 months of their previous ATT regimen. Plasma samples were collected at baseline (1–5 days after commencing ATT), week 8, and week 20 of ATT. We applied network and multivariate analysis to investigate the dynamic inflammatory profile of these patients in relation to ATT and by HIV status. In addition to the discovery cohort, a validation cohort of patients with HIV-1 admitted to hospital with CD4 counts less than 350 cells per μL and a high clinical suspicion of new tuberculosis were recruited.

**Findings:**

Between March 1, 2013, and July 31, 2014, we assessed a cohort of 129 participants (55 [43%] female and 74 [57%] male, median age 35·1 years [IQR 30·1–43·7]) and 76 were co-infected with HIV-1. HIV-1 status markedly influenced the inflammatory profile regardless of ATT duration. HIV-1 viral load emerged as a major factor driving differential inflammatory marker expression and having a strong effect on correlation profiles observed in the HIV-1 co-infected group. Interleukin (IL)-17A emerged as a key correlate of HIV-1-induced inflammation during HIV–tuberculosis co-infection.

**Interpretation:**

Our findings show the effect of HIV-1 co-infection on the complexity of plasma inflammatory profiles in patients with tuberculosis. Through network analysis we identified IL-17A as an important node in HIV–tuberculosis co-infection, thus implicating this cytokine’s capacity to correlate with, and regulate, other inflammatory markers. Further mechanistic studies are required to identify specific IL-17A-related inflammatory pathways mediating immunopathology in HIV–tuberculosis co-infection, which could illuminate targets for future host-directed therapies.

**Funding:**

National Institutes of Health, The Wellcome Trust, UK Research and Innovation, Cancer Research UK, European and Developing Countries Clinical Trials Partnership, and South African Medical Research Council.

## Introduction

Tuberculosis remains one of the most deadly infectious diseases, with 1·2 million deaths in individuals without HIV-1 co-infection and 208 000 deaths in individuals with HIV-1 co-infection reported globally in 2019.^1^ HIV-1 infection is a significant risk factor for tuberculosis infection and although antiretroviral therapy (ART) substantially mitigates tuberculosis risk, it remains higher in individuals with HIV-1 on ART than in individuals without HIV-1.^2^

The influence of HIV-1 co-infection on the immune response to *Mycobacterium tuberculosis* remains poorly understood, as each pathogen compounds the immunopathology associated with the other, adding complexity. Furthermore, the intertwined effects of ART-mediated immune reconstitution and immune clearance of *M tuberculosis* during antitubercular therapy (ATT) makes it difficult to dissect the relative contribution of each to a successful treatment response. There is thus paucity in biomarkers predictive of treatment outcome in HIV–tuberculosis co-infection.

We previously addressed this shortfall by investigating expression of the antioxidant enzyme heme oxygenase 1 (HMOX1) in the context of HIV–tuberculosis co-infection.^3^ HMOX1 expression was significantly higher in patients co-infected with HIV and tuberculosis than in those with tuberculosis alone, with increased expression in those with co-infection being driven by HIV-1 viraemia. HMOX1 expression decreased with ATT irrespective of HIV-1 status, but not in those who had treatment failure or relapse. C-reactive protein and ferritin concentrations also show substantial decline during ATT, except in those with HIV–tuberculosis co-infection in whom a slower decline is seen.^4^ HIV–tuberculosis co-infection induced inflammation appears to take longer than that of tuberculosis alone to resolve. This finding could be explained by high *M tuberculosis* bacterial load, which might occur during advanced immunosuppression, the additive effect of HIV-1-induced inflammation, or the effect of ART initiation. We previously showed that *M tuberculosis* load significantly affects the inflammatory profile of patients with tuberculosis.^4–8^ Systemic dissemination of *M tuberculosis*, more frequently observed in patients with HIV–tuberculosis co-infection, associates with an inflammatory profile distinct from pulmonary tuberculosis, and shows delayed response to ATT by network analysis.^8^ Thus, network analysis is able to detect important underlying trends in the immune profile of tuberculosis cohorts, providing insight into disease pathogenesis. We aimed to use network analysis to investigate the extent to which the HIV-1-induced immune response contributes to the inflammatory profile in pulmonary tuberculosis and the effect of ATT and ART on this inflammatory profile.

## Methods

### Study design and participants

In this prospective cohort study and immunological network analysis, an extensive panel of inflammatory markers was measured in the plasma of a prospective patient cohort. Participants aged 18 years or older with sputum Xpert MTB/RIF-positive rifampicin-susceptible pulmonary tuberculosis were recruited from the Site B clinic, Khayelitsha, Cape Town, South Africa for inclusion in the discovery cohort (see appendix 1 p 2 for eligibility criteria, clinical protocol, and STROBE statement).^3,9^ We did a post-hoc analysis of plasma inflammatory markers measured at baseline, week 8, and week 20 of ATT. One participant with HIV-1 co-infection had values less than the detection range for more than 20% of the analytes and was excluded from the analysis. The University of Cape Town Faculty of Health Sciences Human Research Ethics Committee approved the study (568/2012) and written informed consent was obtained from all study participants.

The validation cohort was part of a larger cohort study,^10^ which was recruited from Jan 16, 2014, until Oct 19, 2016, from patients admitted to the Khayelitsha Hospital, Cape Town, South Africa (appendix 1 p 8). Patients with HIV-1 with a CD4 count less than 350 cells per μL and a high clinical suspicion of new tuberculosis were enrolled (appendix 1 p 2). A control cohort of outpatients with HIV-1 with CD4 counts less than 350 cells per μL and no evidence of tuberculosis (ie, asymptomatic, sputum Xpert MTB/RIF-negative, and no radiographical evidence of tuberculosis) were also recruited from the Site B clinic. All participants provided written informed consent when possible. Those who were eligible, but could not provide informed consent due to decreased level of consciousness were enrolled and followed up daily until they regained the capacity to participate in the informed consent process. University of Cape Town Human Research Ethics Committee approved the study (057/2013) and per-protocol use of samples and information from those who died before providing informed consent, or who could not provide consent by the end of study follow-up.

### Procedures

Participants underwent standardised clinical interview and examination after enrolment. Anonymised data were collected from the patient, their clinical folder, and laboratory results and captured on an access-controlled, secure database. Baseline microbiological testing for *M tuberculosis*, CD4 cell count, and viral load was done by the National Health Laboratory Services (Cape Town, South Africa). Venous blood samples were collected at enrolment from all participants, at week 8 of ATT from the discovery cohort participants, and at week 20 of ATT from those in the discovery cohort who remained sputum culture-positive at week 8. Blood samples underwent laboratory processing, and plasma was isolated and cryopreserved in −80°C freezers until batched extraction.

We evaluated a panel of 38 cytokines, acute phase proteins, and soluble receptors using different immuno-assays in plasma samples. Marker selection was based on their potential role in tuberculosis pathogenesis as shown by previous work by our group^6,11^ and others^12^ and comprised C-reactive protein, fibrinogen, ferritin, procalcitonin, serum amyloid protein A, serum amyloid protein P, α-2-macroglobulin, haptoglobin, tissue plasminogen activator, matrix metalloproteinase (MMP)-1, MMP-3, MMP-7, MMP-8, MMP-9, MMP-10, MMP-12, tissue inhibitor of metalloproteinase (TIMP)-1, TIMP-2, TIMP-3, TIMP-4, interleukin (IL)-1α, IL-1β, IL-1RA, IL-6, IL-8, IL-10, IL-12p70, IL-17A, interferon (IFN)α2, IFNγ, tumour necrosis factor (TNF)α, C-C motif chemokine ligand (CCL)3, CCL4, CCL11, CXCL10, vascular endothelial growth factor (VEGF), soluble CD14, and 8-hydroxy-2’-deoxyguanosine (8-OH-dG). Luminex technology was used to quantify marker concentrations (appendix 1 p 2) and data were log-transformed.

### Statistical analysis

Medians and IQRs were used as measures of central tendency and dispersion. The Mann–Whitney *U* test (for two groups) and the Kruskal-Wallis test were used for group and timepoint comparisons. Fisher’s exact test (2 × 2 comparisons) and Pearson’s χ² (other types of comparisons) were used to compare variables displayed as percentages.

A summary table listing all statistical tests, study timepoints, and relevant cohorts used to construct the figures can be found in appendix 1 (pp 9–10). Statistical analyses were done using GraphPad Prism, version 8.0; STATA, version 11; JMP, version 14.0; and R, version 3.1.0.

Hierarchical cluster analyses (Ward’s method), with 100 × bootstrap^13^ of log_10_-transformed and Z score-normalised data were used to depict the expression profile of plasma biomarkers at the various timepoints in the discovery cohort. Dendrograms display the cluster hierarchy represented by Euclidean distances. Venn diagrams were used to show differentially expressed markers at each study timepoint in the discovery cohort.^14^

Fold change (log_10_) of plasma markers was calculated if relevant. Differences with p values less than 0·05 after Holm-Bonferroni’s adjustment for multiple comparisons were considered statistically significant. All comparisons were prespecified and two-tailed. The Spearman’s rank test was used to assess all correlations between all plasma markers and other variables in the discovery cohort.

Profiles of correlations between biomarkers in different subgroups and timepoints were examined using network analysis of bootstrapped Spearman correlation matrices. We displayed correlation matrices as heatmaps and visualised the networks using circular layout network plots using Gephi, version 0.82, with the circular layout plug-in. Only correlations with a p value less than 0·05 in at least 80 iterations of 100 bootstraps were included in network visualisation. Network density was established calculating the actual connections (known number of connections between nodes) by potential connections (number of potential connections between two nodes; appendix 1 p 13). Node analysis was done to calculate the number of connections per molecule (the arcs are defined as a significant correlation, p<0·05) by timepoint and build a curve of number of nodes over time, and ANOVA linear trends test was used to assess variable dependency. Area under the curve (AUC) was used to measure variation between connection numbers over time and identified the molecules with the most connections.

In the discovery cohort, network analysis was applied to investigate biomarker expression in the overall cohort at different timepoints and to assess the effects of tuberculosis and HIV-1 co-infection, ART, and HIV-1 viraemia on biomarker expression.

Network analysis was also used to measure IL-17A network connectivity in the validation and control cohorts. The numbers of connections with IL-17A were compared between those who died versus those who survived in the validation cohort; those with a CD4 count less than 100 cells per μL versus those with a CD4 count of 100 cells per μL or greater in the control cohort; and those with an HIV-1 viral load of 39 RNA copies per mL or less versus those with a viral load of more than 39 copies per mL. A receiver operating characteristic curve was constructed to evaluate whether the number of connections with IL-17A could predict death in the validation cohort, or low CD4 cell count or high viral load in the control group. For further explanation of the network analysis procedure, see appendix 1 (pp 6–7).

We used binary logistic regression modelling to identify patient characteristics that might be associated with increased network connectivity or density in the discovery cohort and to test whether IL-17A connectivity was associated with death after adjustment for potential confounding variables in the validation cohort (appendix 1 pp 11–12).

### Role of the funding source

The funder of the study had no role in study design, data collection, data analysis, data interpretation, or writing of the report.

## Results

Between March 1, 2013, and July 31, 2014, we enrolled the discovery cohort. It consisted of 129 participants (table), of whom 55 (43%) were female, 74 (57%) were male, and 76 (59%) were co-infected with HIV-1; the median age was 35·1 years (IQR 30·1–43·7). Of the participants with HIV-1, 29 (38%) were on ART and 20 (26%) were virally suppressed (HIV-1 viral load <40 copies per mL) at enrolment. The timepoint of ART initiation was at the discretion of the treating clinician and not standardised; however, most participants commenced ART between 2 and 8 weeks after ATT initiation as per national guidelines in South Africa, with 54 (71%) individuals with HIV-1 on ART by week 8. The overall age distributions of the HIV-1 infected and uninfected subgroups were similar, but there were more men in the HIV-uninfected subgroup and the median body-mass index of the HIV-1 uninfected subgroup was slightly lower. As expected, HIV-1 co-infection was associated with significantly less radiological evidence of extensive disease and the patient’s sputum smear was more commonly negative or scanty positive than that of participants who did not have HIV-1, a higher proportion of whom had higher smear grades (2+ or 3+). Of all the clinical variables recorded (male sex, age, weight, smoking, HIV-1 infection, detection of acid-fast bacilli in sputum, presence of cavities, and sputum culture conversion at week 8), only HIV-1 infection and the presence of cavities on chest radiograph was significantly associated with increased inflammatory network connectivity by binary logistic regression modelling (appendix 1 p 11).

**Figure 1:**
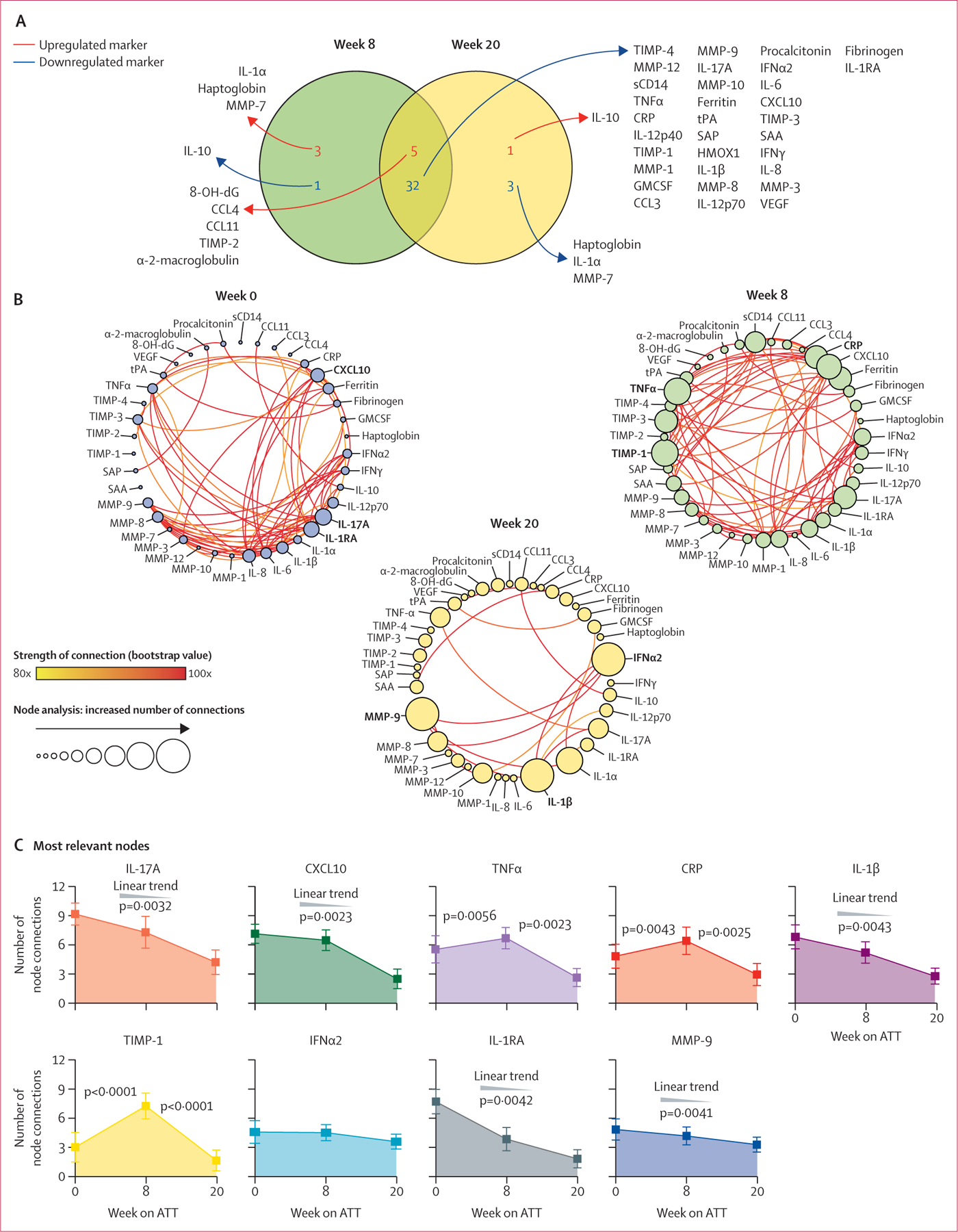
Inflammatory signatures of plasma markers in patients on ATT (A) Venn diagram shows the markers for which values were significantly different between week 8 or week 20 versus week 0 of ATT (p<0·05 after adjustment for multiple comparisons). (B) Network analysis of the biomarker correlation matrices was done with bootstrapping (100 ×). Relationships that remained significant in at least 80 of 100 bootstraps were plotted as connecting lines. Each node represents a different plasma parameter. Circle size is proportional to the number of significant correlations involving that node in each network (sizes of the circles are balanced for each network). The nature of each correlation (positive of negative) is described in appendix 1 (p 15). (C) At each timepoint, the top three markers with the highest number of significant correlations were selected. The number of connections for each marker were compared between the study timepoints using the Freedman’s matched pairs test with Dunn’s multiple comparisons ad hoc test or non-parametric linear trend analysis. p values were adjusted for multiple comparisons using the Holm-Bonferroni method. ATT=antitubercular therapy. CCL=C-C motif chemokine ligand. CRP=C-reactive protein. CXCL10=C-X-C motif chemokine ligand 10. GMCSF=granulocyte-macrophage colony-stimulating factor. IFN=interferon. IL=interleukin. MMP=matrix metalloproteinase. 8-OH-dG=8-hydroxy-2’-deoxyguanosine. SAA=serum amyloid protein A. SAP=serum amyloid protein P. TIMP=tissue inhibitor of metalloproteinase. TNF=tumour necrosis factor. VEGF=vascular endothelial growth factor. tPA=tissue plasminogen activator. HMOX1=heme oxygenase 1. sCD14=soluble CD14.

We examined the expression of inflammatory markers in patients with tuberculosis before starting ATT (n=129), at 8 weeks of ATT (n=129), and at 20 weeks of ATT (n=53) in the discovery cohort. Hierarchical cluster analysis revealed a distinct plasma inflammatory marker expression profile at each of the timepoints (appendix 1 p 14). Although the pre-treatment timepoint was characterised by predominantly higher expression of most inflammatory markers accompanied by a decrease over the course of ATT, there were several markers that did not follow this pattern. To explore this finding further, we compared the expression of these markers at week 8 and week 20 to the pre-treatment timepoint and identified markers that displayed statistically different expression (figure 1A). Concentrations of several markers of inflammation, immune activation, tissue remodelling, and oxidative damage were assessed in plasma samples from patients with pulmonary tuberculosis before and at indicated timepoints after ATT initiation. Most markers showed an early and sustained decrease on ATT.

We used correlation matrices (appendix 1 p 15) and circular layout network plots to visualise differences in correlation profiles between the inflammatory markers and calculated overall network density (figure 1B, appendix 1 p 14). Network density was highest pre-treatment and was not significantly different at week 8; however, a marked decline was observed at week 20 (figure 1B).

We did node analysis of the top nine markers with the greatest amount of significant correlations per timepoint to define inflammatory markers driving overall network density (figure 1C). Considering the number of significant connections for each marker, we calculated the AUC for each and ranked the markers in order of their relative contribution to network density. The AUC value thus reflects the overall capacity of the marker to correlate with others in the network over the three study timepoints. The marker with most network connections was IL-17A, which in turn had a significantly higher AUC than all the other ranked markers (appendix 1 p 14).

**Figure 2:**
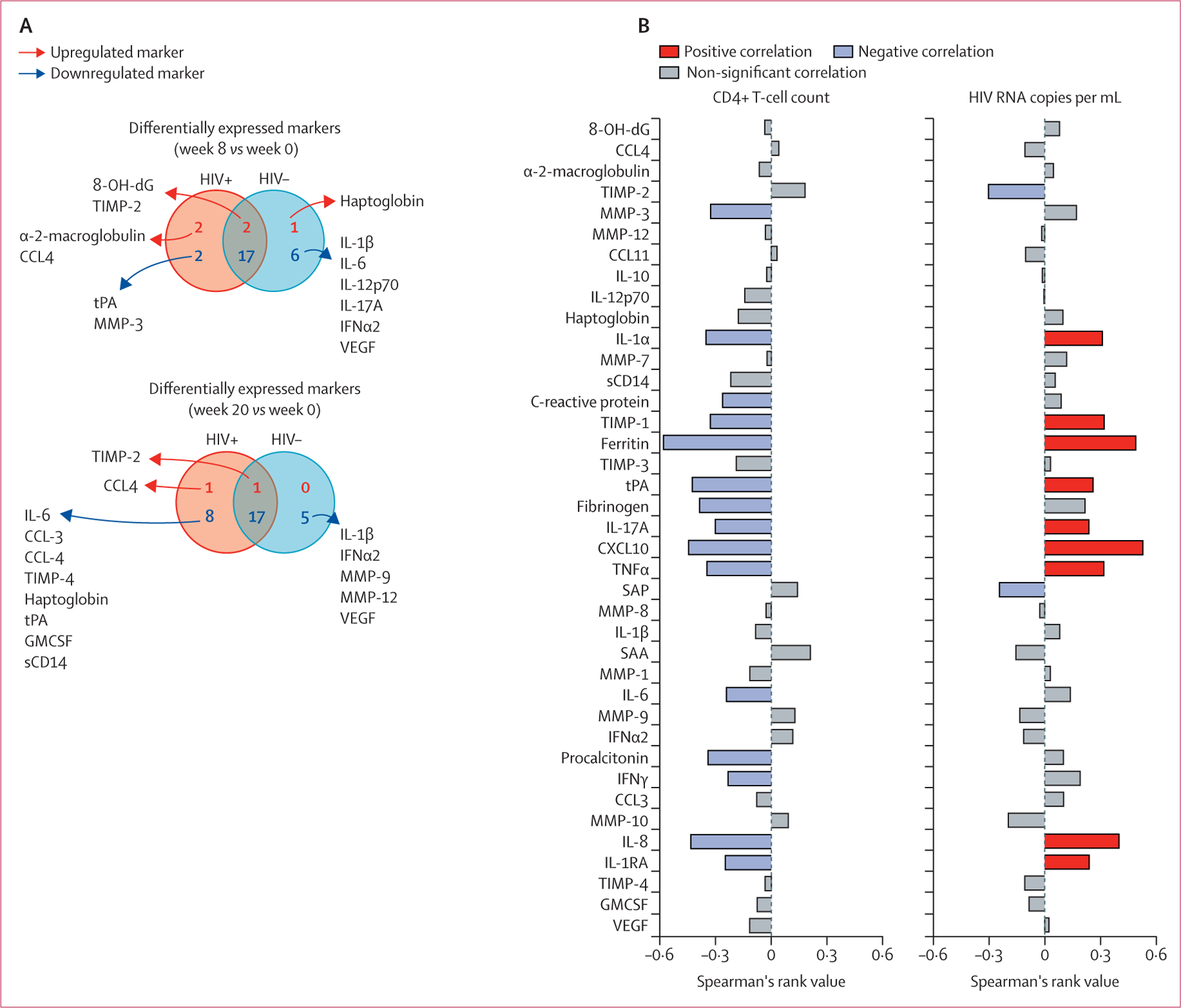
Differential expression of plasma markers in patients with tuberculosis stratified according to HIV-1 infection status (A) Venn diagrams describe the markers for which values were significantly different between week 8 or week 20 versus week 0 of antitubercular therapy in subgroups of patients stratified according to HIV-1 infection status (p<0·05 after adjustment for multiple comparisons). (B) Plasma concentrations of the indicated markers measured before antitubercular therapy initiation were tested for correlations with CD4 T-cell counts and HIV-1 RNA copies using Spearman’s correlation rank test. CCL=C-C motif chemokine ligand. CXCL10=C-X-C motif chemokine ligand 10. MMP=matrix metalloproteinase. IFN=interferon. IL=interleukin. 8-OH-dG=8-hydroxy-2’-deoxyguanosine. SAA=serum amyloid protein A. SAP=serum amyloid protein P. TIMP=tissue inhibitor of metalloproteinase. TNF=tumour necrosis factor. GMCSF=granulocyte-macrophage colony-stimulating factor. VEGF=vascular endothelial growth factor. tPA=tissue plasminogen activator. sCD14=soluble CD14.

Next, we investigated whether HIV-1 co-infection perturbed the plasma inflammatory profile of patients with tuberculosis. Hierarchical clustering revealed significant differences in inflammatory marker concentrations between patients with tuberculosis with HIV-1 (n=76) and without HIV-1 (n=53), which were quantified by calculating fold-change (appendix 1 p 16). Differences were greatest at the pre-treatment timepoint when six inflammatory markers were significantly lower in patients with tuberculosis co-infected with HIV-1 versus patients with tuberculosis without HIV-1 and seven were significantly higher. When comparing differentially expressed markers at the pre-treatment timepoint versus week 8 and week 20 on treatment, most were decreased in both patients with tuberculosis with HIV-1 co-infection and patients with tuberculosis without HIV-1 (figure 2A).

Upon assessing significant correlations of plasma inflammatory markers in the HIV-1 infected subgroup with either CD4 cell count or HIV-1 viral load, we found most markers to show significant negative correlation with CD4 cell count and significant positive correlation with HIV-1 viral load (figure 2B).

Considering the possibility that HIV-1 co-infection could have a substantial effect on individual plasma inflammatory marker expression in patients with tuberculosis, we investigated how this factor influenced correlations between these markers. Correlation matrices of participants with HIV-1 co-infection versus those without provided evidence of differential correlation between the inflammatory markers in each of these subgroups and timepoints (appendix 1 p 18). Network analysis revealed significantly greater density in the participants with HIV-1 co-infection than those without HIV-1 co-infection at pre-treatment and at week 8, with no significant difference observed at week 20 (appendix 1 p 17). A linear decrease in network density was observed in participants with HIV-1 co-infection, whereas those without HIV-1 co-infection showed slightly increased network density compared with pre-treatment at week 8, with a significant decrease only occurring at week 20. The top three markers contributing most to overall network density by AUC in the subgroup of those without HIV-1 co-infection were CXCL10, C-reactive protein, and IFNα2, whereas the top three markers in the subgroup of those with HIV-1 co-infection were IL-17A, IL-1β, and TNFα (appendix 1 p 17). IL-17A was the most influential, and TNFα was the third most influential marker in network connectivity analysis of the cohort as a whole.

To further investigate the effect of HIV-1 on the plasma inflammatory profile, we assessed the effect of ART on network connectivity in HIV–tuberculosis co-infection. 20 (69%) of 29 participants in the ART-established group were virally suppressed and the median CD4 T-cell count was higher (albeit not significantly) in this subgroup than in the ART-naive subgroup (appendix 1 p 19). Although ART status influenced the expression of some of the inflammatory markers in participants with HIV–tuberculosis co-infection at various timepoints, no significant difference was observed between the ART-established and ART-naive subgroups in terms of frequency of extensive disease on chest radiograph, culture positivity at week 8, and unfavourable outcomes (appendix 1 p 19).

Despite only modest differences in individual plasma marker expression being observed between ART-naive and ART-established participants with HIV–tuberculosis co-infection (appendix 1 p 19), network analysis of correlation matrices (appendix 1 p 20) for each timepoint pre-ATT and post-ATT showed marked differences (appendix 1 p 21). Pre-treatment network density was significantly higher in participants who were ART-naive than those established on ART (appendix 1 p 21). Similar network density to that of the pre-treatment timepoint was observed in the ART-naive group despite 8 weeks of ATT, contrasting with the ART-established group at week 8 who displayed significantly decreased network density. At week 20, both ART-naive and ART-established groups displayed similarly low network density. Nodal analysis revealed that IL-17A again had the greatest nodal connectivity in ART-naive participants with HIV–tuberculosis co-infection, with IL-1β and TNFα in second and third place (appendix 1 p 21). In the ART-established group IFNα2, IL-17A, and IL-1β were the markers with greatest nodal connectivity (appendix 1 p 21).

**Figure 3:**
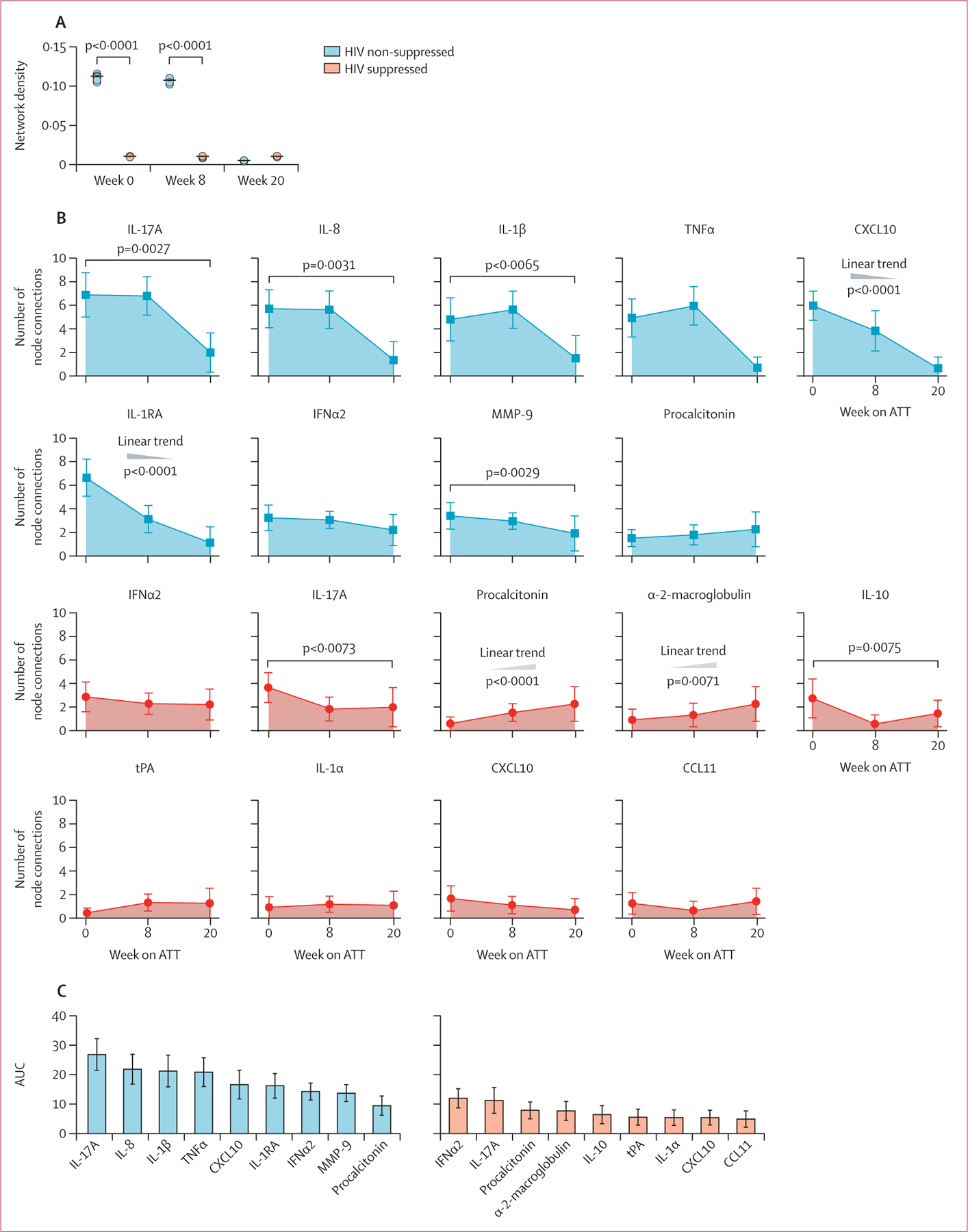
Inflammatory signatures of plasma markers in patients with HIV–tuberculosis co-infection stratified by HIV-1 viral suppression during ATT (A) Network densities of each bootstrap were calculated for each study group and timepoint as described. Data were compared using the Kruskal-Wallis test with Dunn’s multiple comparisons ad hoc test. (B) At each timepoint, the top three markers with the highest number of significant correlations were selected. The number of connections for each marker were compared between the study timepoints using the Friedman’s matched pairs test with Dunn’s multiple comparisons ad hoc test or non-parametric linear trend analysis. p values were adjusted for multiple comparisons using the Holm-Bonferroni method. (C) AUC values for each marker. ATT=antitubercular therapy. AUC=area under the curve. CCL=C-C motif chemokine ligand. CXCL10=C-X-C motif chemokine ligand 10. IFN=interferon. IL=interleukin. MMP=matrix metalloproteinase. TNF=tumour necrosis factor. tPA=tissue plasminogen activator.

Not all ART-established participants were virally suppressed and we hypothesised that HIV-1 viraemia contributed to their observed inflammatory profile. Upon stratifying participants with HIV-1 co-infection by those who had suppressed versus unsuppressed viral loads and using inflammatory network analysis of correlation matrices (appendix 1 p 22), we found that this hypothesis was correct (figure 3A, appendix 1 p 23). Those who had suppressed HIV-1 viral loads had similar low network density at all timepoints, including pre-treatment. Furthermore, those who were viraemic had high network density pre-treatment, no significant change in density at week 8 with significant decrease in network density only occurring at week 20. Nodal analysis again identified IL-17A as the most connected node in those with unsuppressed HIV-1 viral loads (figure 3B, 3C).

Finally, we applied network analysis to two validation studies: one to investigate mortality in patients admitted to hospital with HIV–tuberculosis co-infection and the other examining HIV progression in the absence of active tuberculosis (appendix 1 p 8). The HIV-1 co-infected cohort^10^ consisted of 507 patients admitted to hospital with tuberculosis, of whom 113 died within 12 weeks of enrolment. 29 participants in the outpatient control group had HIV-1 without evidence of tuberculosis. Those of the group admitted to hospital who died tended to be slightly older than those who survived and those in the outpatient control group. 277 (54%) women and 239 (47%) men in the cohort were admitted to hospital, with similar sex distribution by subgroup. The outpatient control group were predominantly female (21 [72%]). Those of the cohort admitted to hospital who died tended to have lower median CD4 cell counts than those who survived and the outpatient controls. Most of the cohort admitted to hospital was not on ART, with only 183 (36%) being on ART at enrolment. ART data are not available for the outpatient control group.

**Figure 4:**
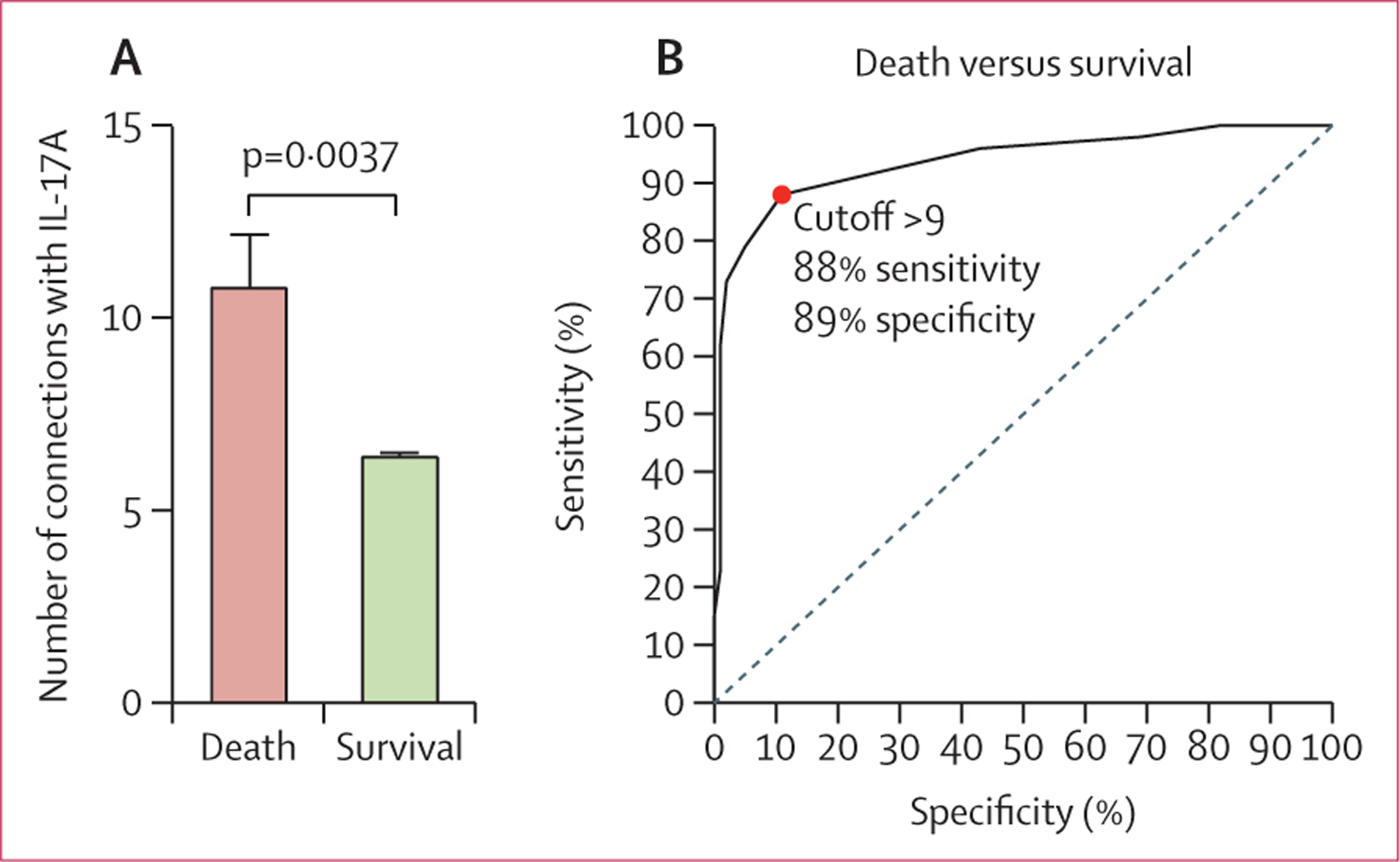
Inflammatory signatures of plasma markers in patients admitted to hospital with HIV–tuberculosis co-infection undergoing antiretroviral therapy, at week 0 of antitubercular therapy (A) Number of connections involving IL-17A in patients in the validation cohort who died versus who survived. (B) Receiver operating characteristic curve to assess whether the number of connections with IL-17A could predict death in the validation cohort. IL=interleukin.

In the co-infected cohort admitted to hospital,^10^ we found the number of connections with IL-17A to be significantly higher in those who died (n=113) than those who survived (n=394; figure 4). Binary logistic regression modelling confirmed that this association was not confounded by the effect of microbiological confirmation of tuberculosis, weight, male sex, or age (appendix 1 p 12). The number of connections with IL-17A through network analysis had 88% sensitivity and 89% specificity to differentiate those who died versus those who survived hospital admission for HIV-1-associated tuberculosis.

In the HIV-1 infected outpatient control group without active tuberculosis, the number of nodal connections with IL-17A was significantly higher in those with lower CD4 cell counts and unsuppressed viral loads (appendix 1 p 24). Conversely, network analysis of the primary discovery cohort showed that tuberculosis infection in the absence of HIV-1 co-infection contributed little to IL-17A network connectivity, even in those with extensive tuberculosis disease on chest radiograph (appendix 1 p 25).

## Discussion

We did a detailed analysis to better understand the effect of HIV-1 co-infection on the inflammatory profile of a well characterised cohort of patients with tuberculosis undergoing ATT. We did not observe any decline in the plasma network density between pre-treatment and week 8 of ATT, and significant decline in density was only observed at week 20 in the discovery cohort. This finding could not be explained by delayed culture conversion, a factor known to significantly influence the plasma inflammatory profile of those with HIV–tuberculosis co-infection and tuberculosis alone,^4–8^ as most participants were no longer sputum culture-positive at week 8. Importantly, stratification by HIV status revealed that network density in the cohort as a whole was driven by that of the subgroup who were HIV-1 co-infected, owing to much greater network density observed at week 8 than that of patients with tuberculosis without HIV-1. When we further stratified the HIV-1 co-infected subgroup by ART status, we found that the ART naive inflammatory profile seemed to underlie the high network density we had observed in the the HIV-1 co-infected group, thus potentially offering explanation for the delayed decline in network density in the cohort as a whole. Most participants who had HIV-1 co-infection were not on ART at enrolment (62%), with 29% yet to commence ART by week 8. Thus, in the absence of availability of viral load data at week 8, and considering that many participants with HIV-1 were newly commenced on ART or still ART naive at this timepoint, it can be assumed that a sizable proportion of the group co-infected with HIV-1 would still have been viraemic at week 8. Dissecting this effect further, we showed that HIV-1 viraemia was driving network connectivity in patients with HIV–tuberculosis co-infection, and that the overall immune profile of those who were virally suppressed had minimal network density. Through network analysis of key inflammatory mediators, we have thus found that HIV-1 viraemia during HIV–tuberculosis co-infection mediates ongoing systemic inflammatory activity, with ART-mediated viral suppression abrogating this effect.

Little is known about the individual contribution of HIV-1 and tuberculosis to systemic immune activation and inflammation observed during co-infection. In-vitro models of macrophage co-infection suggest that *M tuberculosis*-derived gene expression predominates over that of HIV-1, with particular upregulation of pro-inflammatory pathways and type I interferon gene signalling.^15^ Conversely, productive infection of monocyte-derived macrophages with HIV-1 only did not activate inflammatory pathways and was not associated with interferon gene expression,^16^ which was later shown to be part of an HIV-1 mediated immune evasion strategy.^17^ However, our ex-vivo findings indicate that HIV-1 viraemia might play a greater role in promoting inflammation in HIV–tuberculosis co-infection than previously appreciated.

Another important hypothesis-generating finding was that IL-17A was the most important node during network analysis in the cohort as a whole, as well as in the ART naive and HIV-1 virally unsuppressed subgroups, but did not feature in the ranking of the top influential nodes in patients with tuberculosis without HIV. This finding implicates IL-17A and its related immune networks in the inflammatory response to HIV–tuberculosis co-infection, especially in the setting of unsuppressed HIV-1 viral load. By applying network analysis to the HIV–tuberculosis co-infected cohort admitted to hospital, we found that the overall number of connections with IL-17A differentiated those who died and survived hospital admission with high sensitivity and specificity. We showed that in an HIV-1-infected outpatient control group without tuberculosis, advanced HIV-1 as denoted by low CD4 counts (<100 cells per mL) and high viral loads (>39 copies per mL) was associated with higher IL-17A network connectivity, similar to those who died of HIV–tuberculosis co-infection. By contrast, evidence of severe tuberculosis disease on chest radiograph was not associated with markedly increased IL-17A network connectivity. IL-17A network connectivity thus serves as a read-out of higher systemic inflammation and immune activation induced by advanced HIV-1 infection and uncontrolled HIV-1 viral replication, and denotes increased mortality risk in HIV–tuberculosis co-infection.

IL-17A is produced by various cell types with widespread expression of its receptor being observed and its pluripotent effects ranging from mucosal barrier protection to immunopathology in autoimmune disorders.^18,19^ IL-17 could have a beneficial role in tuberculosis by contributing to granuloma maturity; however, it can also induce tissue damage through induction of MMPs.^20–22^ Most studies report higher IL-17 production in latent tuberculosis infection than in active tuberculosis;^23^ thus, there is minimal existing evidence of the induction of IL-17 related cytokine networks by *M tuberculosis* per se. Our findings indicate that increased IL-17A network connectivity is mediated by HIV-1 viraemia and not tuberculosis. HIV-1 Tat protein induces transcriptional dysregulation with particular activation of IL-17 signalling pathways.^24^ Profound depletion of IL-17-producing T-helper (Th)17 cells is observed at mucosal barriers early after HIV-1 infection, leading to chronic immune activation.^25^ The combination of ongoing mucosal inflammation, HIV-1 replication, and *M tuberculosis* co-infection could result in heightened immune activation leading to constant triggering of the remaining IL-17A-producing cell populations during HIV–tuberculosis co-infection. This possibility is exemplified by findings of upregulated Th17 polarising cytokines and transcription factors in pleural fluid of patients with HIV–tuberculosis co-infection with tuberculosis pleurisy, despite numeric depletion of Th17 cells.^26^ It has been shown that IL-17A synergises with other cytokines, especially TNFα and IL-1β to mediate induction of IL-6 and IL-8, and promotes the synthesis of pro-inflammatory cytokines such as TNFα and IL-1β.^27–29^ IL-17A thus displays pleiotropic capacity to influence cytokine signalling and can activate diverse signalling pathways.^18^ These immunological relationships coordinated by IL-17A are reflected through our network analysis findings, with IL-17A, IL-1β, and TNFα featuring as the top three most influential nodes in our HIV-1-co-infected and ART-naive groups. This observation is reproduced in the top four most influential nodes of participants with HIV-1 who are viraemic, with IL-8 showing the second most numerous network connectivity after IL-17A, followed by IL-1β and TNFα.

Our study has limitations. First, we do not know the approximate duration of HIV-1 infection for our participants, a factor that could affect the inflammatory profile. Second, the baseline median CD4 count of the discovery cohort was 192 cells per μL; thus, it is difficult to anticipate to what extent our findings are applicable to those with higher CD4 counts. Third, the timing of ART initiation also varied in the partcipants with HIV-1 infection who were ART-naive at enrolment, precluding analysis to assess for the effect of ART duration. Fourth, only participants who were *M tuberculosis* culture positive donated plasma at week 20, representing a bias inherent to the study design. Fifth, we did not use laboratory testing to exclude all other possible concomitant opportunistic infections, but most of these would have been detected by clinical review. Finally, we measured only soluble inflammatory markers; however, further investigation of our findings using different laboratory technologies such as flow cytometry or transcriptome analysis could provide further insight.

A comprehensive assessment of inflammatory responses in the immune pathogenesis of HIV–tuberculosis co-infection remains challenging owing to numerous potential biomarkers and complex interactions. Here, we add to mounting evidence that network analysis represents a useful, rigorous, statistical approach allowing biomarker discovery and identification of immune pathways associated with disease severity and treatment outcome. Although IL-17A-related inflammatory networks have been extensively studied in autoimmune disease, here, we show for the first time to our knowledge its integral role in HIV–tuberculosis co-infection, concurring with suggestions that autoimmunity might represent an over-looked component of the pathological immune response to tuberculosis.^30^ Further studies are required to confirm whether our finding of upregulated IL-17A-related inflammatory networks in HIV–tuberculosis co-infection are indeed associated with an autoimmune response to tuberculosis, and to elucidate the exact mechanism whereby IL-17A network connectivity is enhanced during co-infection and how HIV-1 viraemia drives this effect. These mechanistic studies might reveal whether IL-17A-related inflammatory pathways are a cause or consequence of detrimental outcomes in HIV-1 and HIV–tuberculosis co-infection and thus potentially inform novel therapeutic targets.

## Supplementary Material

1

2

## Figures and Tables

**Table:** Baseline characteristics of discovery cohort

	All patients with tuberculosis (n=129)	Patients with HIV-1 co-infection (n=76)	Patients without HIV-1 co-infection (n=53)	p value
Sex				
Female	55 (43%)	43 (57%)	12 (23%)	<0·0001
Male	74 (57%)	33 (43%)	41 (77%)	<0·0001
Age, years	35·1 (30·1–43·7)	35·6 (30·6–42·1)	34·9 (27·7–50·7)	0·97
Body-mass index, kg/m^2^	21 (19–23)	22 (20–24)	20 (19–23)	0·037
Diabetes	10 (8%)	5 (7%)	5 (9%)	0·74
Smoking history	60 (47%)	29 (38%)	31 (58%)	0·0013
Acid-fast bacilli smear	..	..	..	0·0074
Negative or scanty	47 (36%)	36 (47%)	11 (21%)	..
1+	23 (18%)	15 (20%)	8 (15%)	..
2+	27 (21%)	12 (16%)	15 (28%)	..
3+	32 (25%)	13 (17%)	19 (36%)	..
Extensive radiological disease	91 (71%)	42 (55%)	49 (92%)	<0·0001
Cavitation	67 (52%)	34 (45%)	33 (62%)	0·075
Culture conversion at week 8	75 (58%)	50 (66%)	25 (47%)	0·054
Deaths	5 (4%)	5 (7%)	0	0·057
Baseline CD4 T cells per μL	..	192·0 (67·5–365·0)	..	..
Baseline HIV-1 viral load, copies per mL	..	108 692 (126–385 633)	..	..
Number on ART	..	29 (38%)	..	..
Number virally suppressed*	..	20 (26%)	..	..

Data are n (%) or median (IQR).

* Viral load less than 40 copies per mL. ART=antiretroviral therapy.

## Research in context

### Evidence before this study

We searched PubMed with the keywords “HIV”, “TB”, “inflammatory markers”, and “treatment” for papers published in English between Jan 1, 2002, and Jan 1, 2020. We found 65 articles that included the search keywords, with 33 of these focused on the immune reconstitution inflammatory syndrome and thus not applicable to our study population. Most of the remaining 32 articles described individual inflammatory markers and their potential to diagnose tuberculosis, monitor disease activity, or treatment response during HIV-1 co-infection. None of the studies used network analysis or other systems immunology approaches.

### Added value of this study

Several previous studies have measured inflammatory marker concentrations in the plasma of patients with HIV–tuberculosis co-infection; however, we show that in-depth examination of relationships between inflammatory markers through network analysis can present a valuable tool to advance knowledge of immunopathological pathways in complex disease states. Our results are the first evidence for a central role of interleukin (IL)-17A-mediated inflammatory responses in HIV–tuberculosis co-infection.

### Implications of all the available evidence

By identifying HIV-1 viraemia as the key driver of inflammation during HIV–tuberculosis co-infection regardless of duration of antitubercular therapy, our findings, along with those of others, form a solid scientific basis for advocating early antiretroviral therapy (ART) initiation in those newly diagnosed with both HIV-1 and tuberculosis and diligent monitoring of HIV-1 viral load in patients with tuberculosis who are established on ART. Furthermore, we identified IL-17A network connectivity as a biomarker of mortality in patients admitted to hospital with HIV–tuberculosis co-infection, thus providing grounds for further mechanistic study of this cytokine.
