# Supplementary material for: Inflammatory profile of patients with tuberculosis with or without HIV-1 co-infection: a prospective cohort study and immunological network analysis
Source: Lancet Microbe. Author manuscript; Available in PMC 2021 Aug 11. (PMC8357308; doi:10.1016/s2666-5247(21)00037-9)
Supplement: 1 [file NIHMS1730385-supplement-1.pdf]

THE LANCET
Microbe

Supplementary appendix 1

This appendix formed part of the original submission and has been peer reviewed. We post it as supplied by the authors.

Supplement to: Du Bruyn E, Fukutani KF, Rockwood N, et al. Inflammatory profile of patients with tuberculosis with or without HIV-1 co-infection: a prospective cohort study and immunological network analysis. *Lancet Microbe* 2021; published online May 17. https://doi.org/10.1016/S2666-5247(21)00037-9.

Appendix 1

Index

	page
**Discovery cohort: eligibility criteria**	**2**
**Discovery cohort: assessment of disease severity and treatment outcome**	**2**
**Validation cohort: definitions and eligibility criteria**	**2**
**Luminex and ELISA kits**	**2**
**Discovery cohort: STROBE Statement**	**3**
**Network analysis procedure**	**6**
**Logistic regression and k-fold cross validation models**	**7**
**Supplementary Tables**	
Supplementary Table 1	**8**
Supplementary Table 2	**9**
Supplementary Table 3	**11**
Supplementary Table 4	**12**
**Supplementary Figures**	
Supplementary Figure 1	**13**
Supplementary Figure 2	**14**
Supplementary Figure 3	**15**
Supplementary Figure 4	**16**
Supplementary Figure 5	**17**
Supplementary Figure 6	**18**
Supplementary Figure 7	**19**
Supplementary Figure 8	**20**
Supplementary Figure 9	**21**
Supplementary Figure 10	**22**
Supplementary Figure 11	**23**
Supplementary Figure 12	**24**
Supplementary Figure 13	**25**
**Appendix References**	**26**
**Clinical Protocol**	**27**

Discovery cohort: eligibility criteria

We recruited patients with sputum Xpert MTB/RIF positive rifampin-susceptible pulmonary TB to the study. Patients were excluded from the primary discovery cohort if they were under the age of 18, unable to commence ATT for any reason, pregnant, had unknown HIV-1 status, were unable to consent to study participation, were unable to provide baseline sputum samples, have had more than 3 doses of ATT or were being re-treated for TB within 6 months of their previous ATT regimen.

Discovery cohort: assessment of disease severity and treatment outcome

Disease extent on chest radiograph was assessed by experts and designated as either "extensive disease" where involvement of > one lung or involvement of ≥ one out of three zones per lung was evident or "mild disease" where only one zone of a lung was involved. All treatment failures, deaths during TB treatment, and true or possible TB recurrences were classified as "unfavorable outcomes". Participants were investigated for disseminated TB to the extent that it would change their management, granted that they all had culturable Mtb from their sputum.

Validation cohort: definitions and eligibility criteria

Only "microbiologically confirmed TB" cases and those with "probable TB" were included in the final analysis. "Probable TB" was classified as those who, in absence of microbiological confirmation, had a positive Alere urine lipoarabinomannan test result and/or had a clinical and radiological picture in keeping with TB, were treated for TB and had no alternative diagnosis made during index admission. Exclusion criteria for the validation cohort were a positive pregnancy test, those who received ATT within the previous month and those who already commenced ATT and had received three or more doses.

Luminex and ELISA kits

Luminex kits for the main cohort were used as follows: (i) C-reactive protein (CRP), fibrinogen, ferritin, procalcitonin, serum amyloid protein A (SAA), serum amyloid protein P (SAP), alpha-2-macroglobulin, haptogloblin and tissue plasminogen activator (tPA) were measured using the Bio-Plex Luminex kits from Bio-Rad (Hercules, CA); (ii) matrix metalloproteinase (MMP)-1, MMP-3, MMP-7, MMP-8, MMP-9, MMP-10 and MMP-12 as well as tissue inhibitor of metalloproteinase (TIMP)-1, TIMP-2, TIMP-3 and TIMP-4 were measured using Luminex kits from R&D Systems (Minneapolis, MN); (iii) interleukin (IL)-1α, IL-1β, IL-1Ra, IL-6, IL-8, IL-10, IL-12p70, IL-17A, interferon (IFN)-α2, IFN-γ, tumor necrosis factor (TNF)-α, CCL3, CCL4, CCL11, CXCL10 and VEGF were measured using a Luminex kit from Millipore (Merk, Burlington, MA). Plasma levels of sCD14 were measured using ELISA (R&D Systems, Quantikine). Concentrations of 8-hydroxy-2'-deoxyguanosine (8-OH-dG) were assessed by an ELISA from Cayman Chemical (Ann Harbor, MI).

Discovery cohort: STROBE Statement

	Item No	Recommendation	Page No
**Title and abstract**	1	(*a*) Indicate the study's design with a commonly used term in the title or the abstract<br>**Our study design is indicated in the title and abstract (prospective cohort study)**<br>(*b*) Provide in the abstract an informative and balanced summary of what was done and what was found<br>**We provide a summary of what was done and our findings in the abstract under Background, Methods and Findings**	1, 2<br><br>2
**Introduction**			
Background/rationale	2	Explain the scientific background and rationale for the investigation being reported<br>**The scientific background and rationale for our investigations are detailed in the Introduction section**	4
Objectives	3	State specific objectives, including any prespecified hypotheses<br>**Our objectives and hypotheses are listed in the final paragraph of our introduction**	4
**Methods**			
Study design	4	Present key elements of study design early in the paper<br>**This is presented in the abstract and in the materials and methods section early in the paper**	2, 4
Setting	5	Describe the setting, locations, and relevant dates, including periods of recruitment, exposure, follow-up, and data collection<br>**This is described in the abstract and materials and methods section**	2, 4
Participants	6	(*a*) Give the eligibility criteria, and the sources and methods of selection of participants. Describe methods of follow-up<br>**This is described in the materials and methods section**<br>(*b*) For matched studies, give matching criteria and number of exposed and unexposed<br>**Not applicable**	4
Variables	7	Clearly define all outcomes, exposures, predictors, potential confounders, and effect modifiers. Give diagnostic criteria, if applicable<br>**This is described in the materials and methods section**	4
Data sources/ measurement	8	For each variable of interest, give sources of data and details of methods of assessment (measurement). Describe comparability of assessment methods if there is more than one group<br>**This is described in the materials and methods section: immunoassays**	5
Bias	9	Describe any efforts to address potential sources of bias<br>**We used a random selection method if there were more potentially eligible patients than we had capacity to enrol. We randomly selected samples to test immunological markers (see Materials and Methods: immunoassays, immunoassay analysis).**	4, 5
Study size	10	Explain how the study size was arrived at<br>**Statistical power calculations were described in the original protocol. Please refer to reference in Material and Methods section Rockwood *et al.* (https://doi.org/10.1093/infdis/jix337), this is further described in the Statistical Analyses section of the publication.**	4
Quantitative variables	11	Explain how quantitative variables were handled in the analyses. If applicable, describe which groupings were chosen and why	5

		**This is described in the materials and methods section: immunoassay analysis**	
Statistical methods	12	*(a)* Describe all statistical methods, including those used to control for confounding<br>**This is described in the materials and methods section: immunoassay analysis, network analysis**	5
		(*b*) Describe any methods used to examine subgroups and interactions<br>**This is described in the materials and methods section: immunoassay analysis, network analysis**	5
		(*c*) Explain how missing data were addressed	5
		(*d*) If applicable, explain how loss to follow-up was addressed	5
		(*e*) Describe any sensitivity analyses<br>**We describe all analyses in detail in the Materials and Methods section: immunoassay analysis, network analysis**	5
**Results**			
Participants	13	(a) Report numbers of individuals at each stage of study—eg numbers potentially eligible, examined for eligibility, confirmed eligible, included in the study, completing follow-up, and analysed<br>(b) Give reasons for non-participation at each stage<br>(c) Consider use of a flow diagram<br>**All of the above is described in the materials and methods section under "Primary discovery cohort: clinical characteristics" and a flow diagram is provided as supplementary figure 1**	4, 6
Descriptive data	14	(a) Give characteristics of study participants (eg demographic, clinical, social) and information on exposures and potential confounders<br>**We describe this in the first paragraph of the results section and in supplementary table 1**	6
		(b) Indicate number of participants with missing data for each variable of interest	
		(c) Summarise follow-up time (eg, average and total amount)<br>**Patients from the primary discovery cohort were followed up clinically for six months (duration of ATT), patients in the validation cohort were followed up for 12 weeks; we describe this in Materials and Methods section**	5
Outcome data	15	Report numbers of outcome events or summary measures over time<br>**We describe this in the results section, in table 1 and further information is available in the previous publication by Rockwood *et al.* (https://doi.org/10.1093/infdis/jix337)**	6

Main results	16	(a) Give unadjusted estimates and, if applicable, confounder-adjusted estimates and their precision (eg, 95% confidence interval). Make clear which confounders were adjusted for and why they were included<br>**We describe all statistical adjustments to limit for confounders in the Materials and Methods section: immunoassay analysis, network analysis. Differences with p values below 0·05 after Holm-Bonferroni's adjustment for multiple comparisons were considered statistically significant.**<br>(*b*) Report category boundaries when continuous variables were categorized<br>**We report category boundaries were relevant in the results section and in our figures as it pertains to CD4 count and HIV viral load.**<br>(*c*) If relevant, consider translating estimates of relative risk into absolute risk for a meaningful time period<br>**Not applicable**	5<br><br><br><br><br><br><br>6-16
Other analyses	17	Report other analyses done—eg analyses of subgroups and interactions, and sensitivity analyses<br>**We describe all analyses in detail in the Materials and Methods section: immunoassay analysis, network analysis**	5
**Discussion**			
Key results	18	Summarise key results with reference to study objectives<br>**This is provided under *Findings* in the abstract**	2
Limitations	19	Discuss limitations of the study, taking into account sources of potential bias or imprecision. Discuss both direction and magnitude of any potential bias<br>**This is provided in the second last paragraph of the discussion**	8
Interpretation	20	Give a cautious overall interpretation of results considering objectives, limitations, multiplicity of analyses, results from similar studies, and other relevant evidence<br>**A summary of this is provided in the abstract under *Interpretation* and the results are discussed in the context of relevant evidence in the Discussion section**	2, 8
Generalisability	21	Discuss the generalisability (external validity) of the study results<br>**This is provided in the Discussion section**	8
**Other information**			
Funding	22	Give the source of funding and the role of the funders for the present study and, if applicable, for the original study on which the present article is based<br>**A summary of this is provided in the abstract under Funding and this is further detailed under Funding Information on page 9**	2, 9

Network analysis procedure

Profiles of correlations between biomarkers in different subgroups and timepoints were examined using network analysis of Boostraped spearman correlation matrices (Supplementary Fig. 2, 4 and 6). Only strong correlations, defined as having a significant p-value (<0·05) in at least 80+ iterations of 100 bootstraps, were included in network visualization. The node analysis using replication of each network at different timepoints was performed by calculating the number of connections per molecule (the arcs are defined as a significant correlation, p<0·05) by time point and network density (ND) was established calculating the actual connection [known connections number between nodes (AC)] by potential connection [A connection number that could potentially exist between two nodes (PC)]:
PC = n*(n-1)/2
Network Density = AC/PC
AC = Actual Connections
PC = Potential Connections
n = Number of nodes
The Networks figures were designed in Gephi™ 0.82 with circular layout plug-in (further overview of this is provided in supplementary figure 1).

Network analysis of plama inflammatory markers measured in the discovery cohort at the study timepoints are thus visualized as *circular layout network* plots (Fig. 1C, Supplementary Fig. 3A, Supplementary Fig. 7A, Fig. 3A and Supplementary Fig. 10A) with the direction of each correlation (positive or negative) depicted in the correlation matrices, displayed as heatmaps (Supplementary Figures 2, 4 and 6). Correlation matrices were built for each timepoint and were submitted to 100x bootstrap. The heatmaps show correlations that persisted with a significant p-value (false discovery rate of 5%) in at least 80 times out of 100 bootstraps.

Next, overall network density per study timepoint was calculated as described above (Fig. 1D, Supplementary Fig. 3B, Supplementary Fig. 7B, Fig. 3B and Supplementary Fig. 10B), with the top markers in terms of statistically significant correlations at each timepoint and for each comparison group being identified (Fig. 1E, Supplementary Fig. 3C, Supplementary Fig. 7C, Fig. 3C, Supplementary Fig. 10C). Data were compared between groups and timepoints (for summary see supplementary table 2) using the Kruskal-Wallis test with Dunn's multiple comparisons *ad hoc* test. The comparison between network densities was established by permutation test with 10000x permutation developed in native R. Each marker was considered individually, with comparison being made between the amounts of statistically significant correlations with other plasma markers for each comparator group, at each timepoint (Friedman's matched pairs test with Dunn's multiple comparisons *ad hoc* test or non-parametric linear trend analysis). In addition, at each timepoint and in each of the study groups the number of connections were used to build a curve and ANOVA linear trends test to assess variable dependency. The area under the curve (AUC) was used to measure variation between connection numbers over time and identified molecules with most connections overall for the three study visits (Fig. 1F, Supplementary Fig. 3D, Supplementary Fig.7D, Fig, 3D and Supplementary Fig. 10D.

Finally, we turned to the validation cohort to measure IL-17A network connectivity in a hospitalised cohort of HIV-TB patients (Fig. 4) and compared the number of connections with IL-17A between those in the cohort who died and those who survived (Fig. 4A). A receiver operating characteristic (ROC) curve was constructed to evaluate whether the number of connections with IL-17A could predict death in the validation cohort (Fig. 4B). The optimal cut-off value was determined and the percentage of expected true positive, false positive, true negative and false negative results using this cut-off are visually displayed in Fig. 4C. The same approach was used in the HIV-1 infected, non-TB outpatient control group where the number of connections with IL-17A during network analysis were compared between those with a CD4 count below 100 cells/µl and those with a CD4 count greater than or equal to 100 cells/µl (supplementary figure 9A). This was repeated in the same control group, this time comparing those with an HIV-1 viral load equal to or below 39 RNA copies/mL with those with a viral load (VL) over 39 copies/mL (supplementary figure 9B).

The correlation network has been constructed using all cytokine measurements from all patients in a correlation matrix. Each Network has its own density value and the comparison statistics were obtained by bootstrap (x100) re-sampling values. We did not employ stratification of the population by age, sex and comorbidities to avoid losing statistical power, but the multivariate analyses were performed and are displayed in supplementary tables 3 and 4. The Network Density does not have directions and it may be influenced by both negative of positive correlations. Indeed, enrichment

pathway analysis used in "omic" studies employ the exact same principle. In network density analysis, regulation of processes, rather than direction of influence, is computed. By definition, the network density measure how dense is the network. Networks with higher density values are thought to me more tightly regulated/coordinated, as previously described by our group in a number of publications[1-11]. Of note, our group has created such network analysis in the context of plasma biomarkers. With respect to the correlation direction: the direction of the correlations which were statistically significant in >80 of out 100 bootstraps are depicted in the heatmaps of the Spearman rank correlation matrices in the supplementary material (Supplementary Figures 2, 4, 6 and 8).

The Spearman rank correlation matrices expresses the similarity pair to pair between the two sets. The correlation matrix is square, symmetrical and the diagonal represents the autocorrelations. Also, it can be used as a dissimilarity matrix employed as a distance measurement between the pairs and applied to build a network. All the networks are based in a dissimilarity matrix with nodes (molecules) and edges (connections between molecules) presented as positive or negative correlations or the significance values of the pair of two correlated molecules. The advantage of transforming the correlation matrix in network is to use the network measurements, providing a topography analysis, such as: node analysis, network density, betweeness, centrality and other measurements. Our network analysis was performed by using the significant connections from the correlation matrix. Other differences between the network analysis and the correlation matrices in the supplementary material are: The network was employed with a bootstrap analysis it means several rounds of resampling were performed to obtain a robust consensus network and only consistent connection (+80 significant between resampling) were depicted; and the correlation matrix was performed without resampling to give an idea of correlation directions. The resampling process is random. Thus serial resampling adds to the robustness of the network analysis findings, and is thus significantly more stringent than that of the simple single round of comparative analysis conducted in the correlation matrices.

Logistic regression and k-fold cross validation models

We performed binary logistic regression modelling to identify patient characteristics aside from HIV infection status that may be associated with increased network connectivity/density in the discovery cohort (supplementary table 3). In the first instance the model was used to test association between the parameters and increases of 1 unit in network density (connectivity) of the entire study population. The resampling was performed to evaluate the model on the data sample, using a parameter called "k" that refers to the number of groups the data sample was split into. One proportion of the data was used as training dataset and the other as validation set thus using a limited sample to infer how the model is expected to perform when used to make predictions on data not used during the training of the model. The k (10) Fold Cross Validation was performed using classification and Regression Training package (CARET) available in R. Of all the variables analysed, including male sex, age, weight, smoking, HIV-1 infection, detection of acid-fast bacilli in sputum, presence of cavities and sputum culture conversion at week eight, only HIV-1 infection and the presence of cavities on chest radiograph significantly associated with increased inflammatory network connectivity (supplementary table 3).

A similar statistical model was employed to test whether IL-17A connectivity was associated with death after adjustment for potential confounding variables in the validation (supplementary table 4). Thus, binary logistic regression with k-fold cross validation modelling, as described in the aforementioned, was employed to evaluate for the possible effect of microbiologic confirmation of TB, male sex, age, weight and IL-17A network connectivity on mortality in the validation cohort (supplementary table 4). Off all these parameters, only IL-17A network connectivity was significantly associated with mortality.

Supplementary table 1. Characteristics of the validation cohort and HIV infected outpatient control group

Study group	Survived	Died	HIV infected outpatient control	*p*-value
N	**394**	**113**	**29**	
**Male**	191 (48·5)	48 (42·5)	8 (27·6)	0·06414
**Age (years)**	35·5 (30·5 - 42·1)	39·3 (32·1 - 46·7)	34·2 (29·9 - 41·5)	0·00791
**Sputum GeneXpert**				<0·00001
Inconclusive	1 (0·3)	0	0	
MTB detected	218 (66·1)	59 (79·7)	0	
MTB negative	111 (33·6)	15 (20·3)	24 (82·8)	
Unable to produce sputum	0	0	5 (17·2)	
**Sputum culture**				<0·00001
AFB	2 (0·7)	0	0	
contaminated	16 (5·5)	1 (1·5)	1 (3·4)	
MTB	205 (70·0)	54 (83·1)	0	
negative	70 (23·9)	8 (12·3)	23 (79·3)	
NTM	0	2 (3·1)	0	
Unable to produce sputum	0	0	5 (17·2)	
**Evidence of TB on CXR**	369 (96·3)	105 (97·2)	0	..
**CRP**	148·1 (88·7 - 225·3)	173 (111·3 – 262)	..	0·00785
**Haemoglobin**	8·7 (7·4 - 10·5)	7·9 (6·7 – 10)	12·1 (10·8 - 13.2)	<0·00001
**White cell count**	7·1 (4·5 - 10·2)	7·3 (4·6 - 11·3)	4 (3·4 - 5·1)	0·00001
**Platelets**	274 (190 - 358·3)	233·5 (136 - 316·8)	249 (199 – 300)	0·00293
**CD4 T (cells/µl)**	64·5 (23 - 126·5)	39 (17 – 88)	159 (74 – 204)	<0·00001
**HIV viral load (copies/ml)**	126585 (2686·5 - 489842·5)	114228 (1921 – 625454)	23375 (39 – 101396)	0·02910
**ART status**				..
Defaulted	90 (22·8)	31 (27·7)	..	..
Naive	161 (40·9)	41 (36·6)	..	..
Established on ART	143 (36·3)	40 (35·7)	..	..

Data above reflect the number (%) except for age, CRP, Haemoglobin, White cell count, Platelets, CD4 and HIV viral load where the median value (interquartile range) is displayed. MTB: *Mycobacterium tuberculosis*. AFB: acid-fast bacilli. NTM: Nontuberculous mycobacteria. TB: tuberculosis. CRP: C-Reactive Protein. ART: anti-retroviral therapy.

Supplementary table 2. Summary of statistical tests applied by study cohort, comparator group and timepoint

Timepoints	Comparitor groups	Cohort	Statistical tests applied (corresponding Figures)
Week 0, 8 & 20	entire cohort at specified study timepoints	discovery cohort	- Hierarchical cluster analysis (Fig. 1A)<br>- Venn Diagram (Fig. 1B)<br>- Network analysis (Fig. 1C)<br>- Correlation matrices (Supplementary Fig. 2)<br>- Network density: Kruskal-Wallis test with Dunn's multiple comparisons (Fig. 1D)<br>- Top correlating markers: Friedman's matched pairs test with Dunn's multiple comparisons, Holm-Bonferroni correction for multiple comparisons (Fig. 1E)<br>- ANOVA linear trends test, Area Under the Curve (AUC) (Fig. 1F)
Week 0, 8 & 20	HIV-1 infected versus - uninfected group	discovery cohort	- Hierarchical cluster analysis (Fig. 2A)<br>- Fold change with Holm-Bonferroni's adjustment for multiple comparisons (Fig. 2A)<br>- Venn Diagram (Fig. 2B)<br>- Spearman rank correlation (Fig. 2C)<br>- Network analysis (Supplementary Fig. 3A)<br>- Correlation matrices (Supplementary Fig. 4)<br>- Network density: Kruskal-Wallis test with Dunn's multiple comparisons (Supplementary Fig. 3B)<br>- Top correlating markers: Friedman's matched pairs test with Dunn's multiple comparisons, Holm-Bonferroni correction for multiple comparisons (Supplementary Fig. 3C)<br>- ANOVA linear trends test, AUC (Supplementary Fig. 3D)
Week 0, 8 & 20	HIV-1 infected group: ART established versus the ART-naïve group	discovery cohort	- Hierarchical cluster analysis (Supplementary Fig. 5A)<br>- Fold change with Holm-Bonferroni's adjustment for multiple comparisons (Supplementary Fig. 5B)<br>- Network analysis (Supplementary Fig. 7A)<br>- Correlation matrices (Supplementary Fig. 6)<br>- Network density: Kruskal-Wallis test with Dunn's multiple comparisons (Supplementary Fig. 7B)<br>- Top correlating markers: Friedman's matched pairs test with Dunn's multiple comparisons, Holm-Bonferroni correction for multiple comparisons (Supplementary Fig. 7C)<br>- ANOVA linear trends test, AUC (Supplementary Fig. 7D)
Week 0, 8 & 20	HIV-1 infected group: HIV virally suppressed versus –unsuppressed group	discovery cohort	- Network analysis (Fig. 3A)<br>- Correlation matrices (Supplementary Fig. 8)<br>- Network density: Kruskal-Wallis test with Dunn's multiple comparisons (Fig. 3B)<br>- Top correlating markers: Friedman's matched pairs test with Dunn's multiple comparisons, Holm-Bonferroni correction for multiple comparisons (Fig. 3C)<br>- ANOVA linear trends test, AUC (Fig. 3D)

Week 0, 8 & 20	those with mild radiographic disease extent compared to those with evidence of severe disease on chest radiograph	discovery cohort	- Network analysis (Supplementary Fig. 10A)<br>- Network density: Kruskal-Wallis test with Dunn's multiple comparisons (Supplementary Fig. 10B)<br>- Top correlating markers: Friedman's matched pairs test with Dunn's multiple comparisons, Holm-Bonferroni correction for multiple comparisons (Supplementary Fig. 10C)<br>- ANOVA linear trends test, AUC (Supplementary Fig. 10D)
Week 0 (enrolment)	Participants who died versus survived	validation cohort	- Network analysis (Fig 4A)<br>- Receiver Operating Characteristic (ROC) curve (Fig 4B)<br>- true positive, false positive, true negative and false negative rates at given specificity and sensitivity (Fig 4C)
Week 0 (enrolment)	Participants with a CD4 count < 100 cells/µl versus a CD4 count ≥ to 100 cells/µl	HIV-1 infected, non-TB outpatient control group	- Network analysis (Supplementary Fig. 9A)<br>- Receiver Operating Characteristic (ROC) curve (Supplementary Fig. 9A)<br>- true positive, false positive, true negative and false negative rates at given specificity and sensitivity (Supplementary Fig. 9A)
Week 0 (enrolment	Participants with a CD4 count < 100 cells/µl and those with a CD4 count ≥ 100 cells/µl	HIV-1 infected, non-TB outpatient control group	- Network analysis (Supplementary Fig. 9B)<br>- Receiver Operating Characteristic (ROC) curve (Supplementary Fig. 9B)<br>- true positive, false positive, true negative and false negative rates at given specificity and sensitivity (Supplementary Fig. 9B)

Parameter	adjusted OR	95% CI		p-value	adjusted OR (10k-fold validation)	95% CI		p-value
		Lower	Upper			Lower	Upper	
**Male**	1•05	0•15	1•18	0•728	1•09	0•43	1•32	0•526
**Age** (per 1 year increase)	1•01	0•95	1•08	0•450	1•00	0•87	1•01	0•465
**Weight** (per 1Kg increase)	0•99	0•92	1•02	0•089	0•98	0•86	1•00	0•110
**Smoking history**	1•08	0•98	1•96	0•072	1•05	0•99	1•25	0•065
**HIV infection**	3•42	1•25	5•32	**<0•001**	2•55	1•12	4•38	**<0•001**
**AFB positive**	1•20	0•92	1•66	0•072	1•18	0•92	1•55	0•081
**Lung cavitary lesion**	1•84	1•12	3•45	**0•030**	1•22	1•01	2•20	**0•038**
**Culture conversion at week 8**	1•02	0•85	1•66	0•089	1•00	0•92	1•33	0•072

Supplementary Table 3. Factors associated with increased inflammatory network density. Binary logistic regression and k-fold cross validation model for increased connectivity in the networks. The models were used to test association between the indicated parameters and increases of 1 unit in network density (connectivity) of the entire study population. The resampling was performed to evaluate the model on the data sample, using a parameter called "k" that refers to the number of groups the data sample was split into. One proportion of the data was used to discovery the classification and the rest to validate and measure the prediction power of limited data. The k (10) Fold Cross Validation was performed using classification and Regression Training package (CARET) available in R. P-values in bold-type font indicate statistically significant.

Abbreviations: CI: confidence interval; AFB: acid-fast bacilli

Parameter	adjusted OR	95% CI		p-value	adjusted OR (10k-fold validation)	95% CI		p-value
		Lower	Upper			Lower	Upper	
**Microbiologically Confirmed TB**	1•52	0•72	3•17	0•270	1•60	0•23	1•73	0•281
**Male**	1•29	0•42	2•33	0•138	1•45	0•80	2•61	0•220
**Age** (per 1 year increase)	1•03	1•01	1•06	0•006	1•02	1•00	1•05	0•066
**Weight** (per 1Kg increase)	0•98	0•96	1•00	0•062	0•98	0•95	1•00	0•068
**IL-17A** (per 1log^{10} increase in connectivity)	2•10	1•03	3•37	0•001	2•10	1•02	2•51	0•005

Supplementary Table 4. Binary logistic regression and k-fold cross validation model for mortality. The resampling was performed to evaluate the model on the data sample, using a parameter called "k" that refers to the number of groups the data sample was split into. One proportion of the data was used to discovery the classification and the rest to validate and measure the prediction power of limited data. The k (10) Fold Cross Validation was performed using classification and Regression Training package (CARET) available in R.

Abbreviations: CI: confidence interval; MTB: Mycobacterium tuberculosis

Supplementary Figures

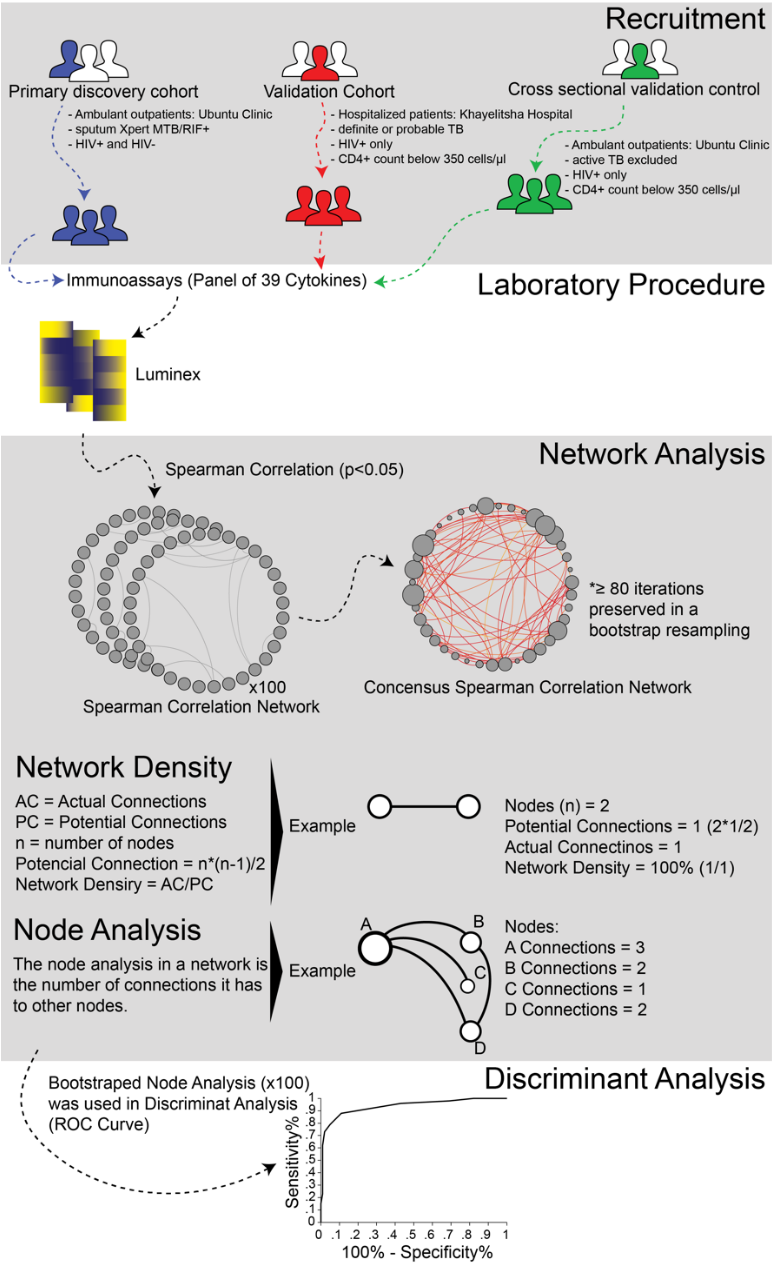

Supplementary Figure 1. Outline of the network analysis. Data from three different studies were used. Description of the studies is shown in the Methods section. Cryopreserved plasma samples were used in Luminex assays to quantify concentrations of several proteins and surrogates of systemic inflammation, immune activation and tissue remodeling. Data were extracted and Spearman correlation matrices for each clinical subgroup and timepoint were calculated. Each resulting matrix was submitted to 100x bootstrap. The Networks display correlations that persisted with a significant p-value (false discovery rate of 5%) in at least 80 times out of 100 bootstraps. Network density was calculated, and node analysis was performed for each clinical subgroup and timepoint as described in the Methods section. The number of connections of a given node (e.g. IL-17A) was inputted in a Receiver Operator Characteristics (ROC) curve analysis to test the discriminant power to predict mortality in the validation cohort.

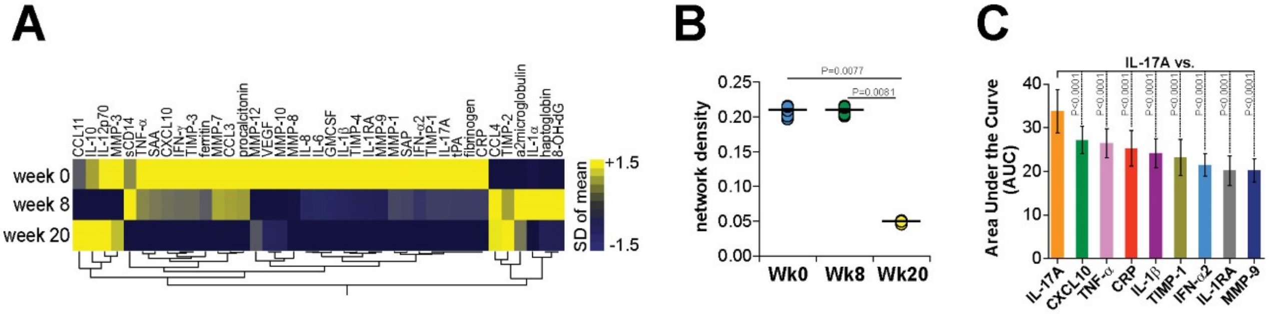

Supplementary Figure 2. Inflammatory signatures of plasma markers in pulmonary TB patients undergoing antitubercular therapy. Concentrations of several markers of inflammation, immune activation, tissue remodeling and oxidative damage were assessed in plasma samples from a cohort of 129 pulmonary TB patients before and at indicated timepoints after anti-TB treatment initiation. Luminex technology was used to quantify the markers. Data were log-transformed.

(A) Hierarchical cluster analysis using z-scored values of each parameter (Ward's method) was employed to depict the overall biomarker expression profile in the study population at the different timepoints.

(B) Network densities of each bootstrap were calculated for each study group and timepoint as described in Materials and Methods. Data were compared using the Kruskal-Wallis test with Dunn's multiple comparisons *ad hoc* test.

(C) At each timepoint, the top three markers with the highest number of statistically significant correlations were selected. The number of connections for each marker were compared between the study timepoints using the Freedman's matched pairs test with Dunn's multiple comparisons *ad hoc* test or non-parametric linear trend analysis.P-values were adjusted for multiple comparisons using the Holm-Bonferroni method. Area under the curves (AUC) values were calculated for each marker shown in (Fig. 1C).

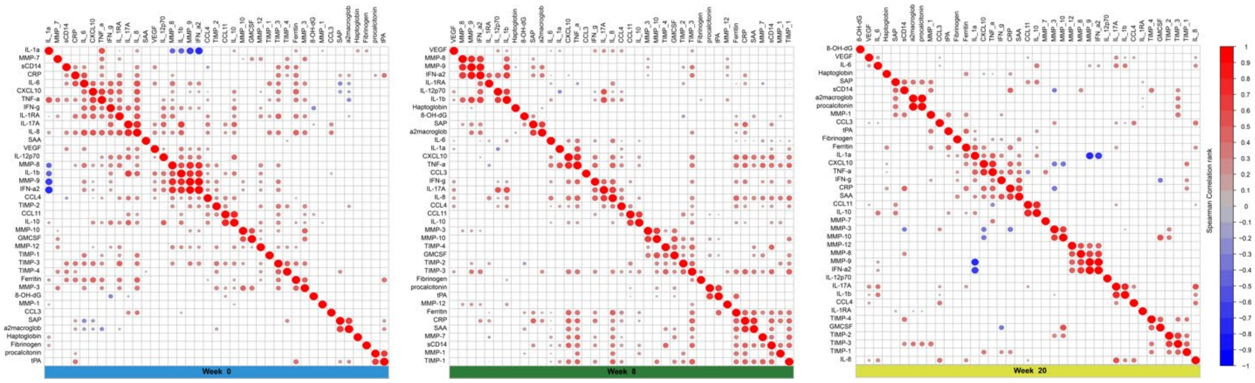

Supplementary Figure 3. Heatmap of the Spearman rank coefficient values of the correlations between plasma inflammatory markers in the discovery cohort at weeks 0, 8 and 20. Correlation matrices were built for each timepoint and were submitted to 100x bootstrap. The graphs show correlations that persisted with a significant p-value (false discovery rate of 5%) in at least 80 times out of 100 bootstraps. Red circles indicate positive correlations whereas blue circles denote negative correlations. Size of the circles is proportional to the strength of the correlation.

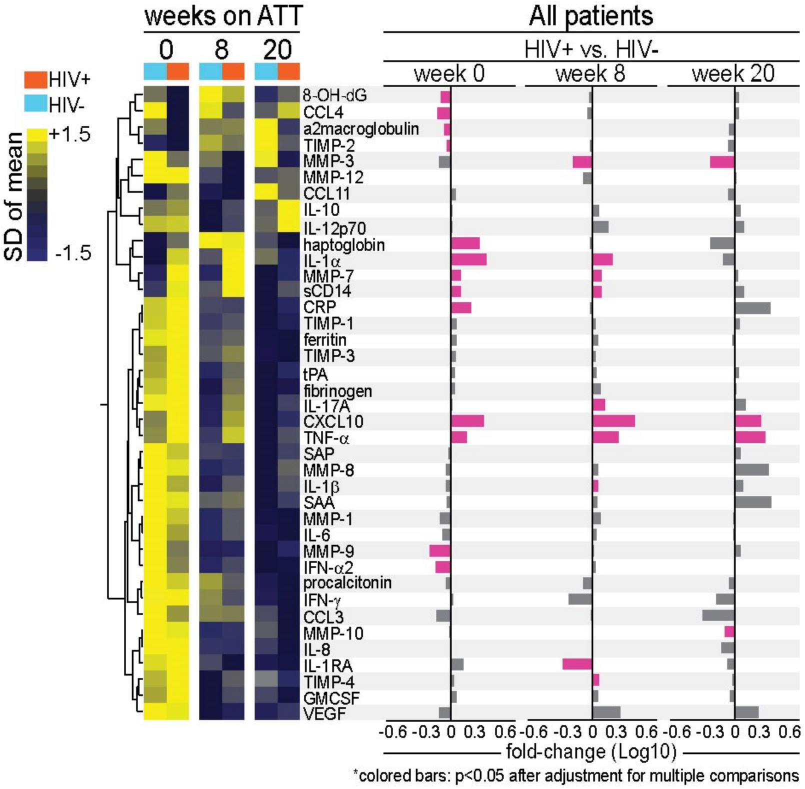

Supplementary Figure 4. Differential expression of plasma markers in pulmonary TB patients stratified according to HIV-1 infection status.

Hierarchical cluster analysis using z-scored values of each parameter (Ward's method) was employed to depict the overall biomarker expression profile in the study population at the different timepoints of antitubercular therapy (ATT) and stratified according to HIV-1 infection status. Fold-variation values (HIV-1 positive vs HIV-1 negative) were calculated and statistically significant differences of several biomarkers are highlighted in pink.

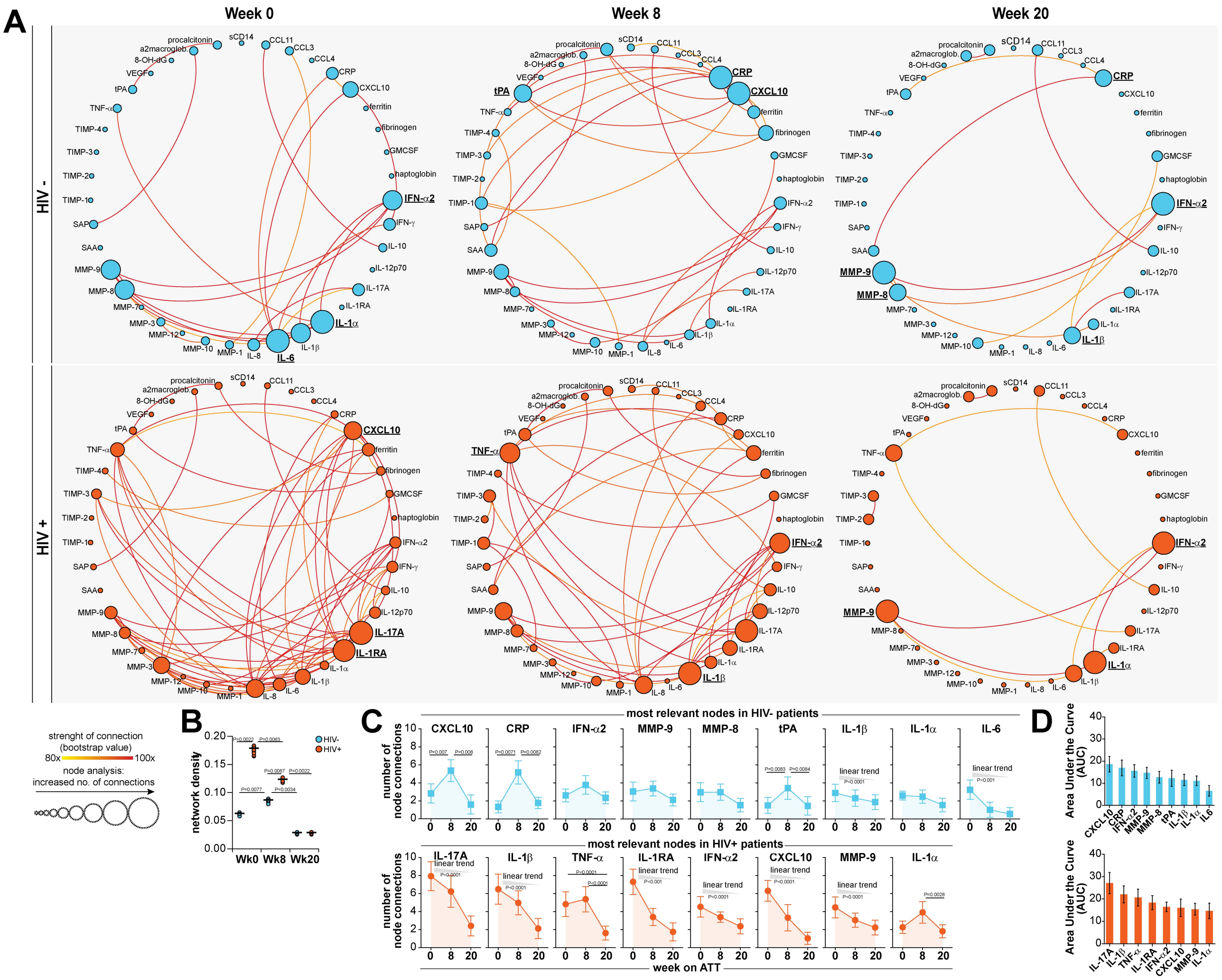

Supplementary Figure 5. Inflammatory signatures of plasma markers in pulmonary TB patients with and without HIV-1 co-infection.

(A) Network analysis of the biomarker correlation matrices was performed with bootstrap (100x). Relationships which remained statistically significant in at least 80 out of 100 bootstraps were plotted as connecting lines. Each node represents a different plasma parameter. The size of each circle is proportional to the number of significant correlations involving such node in each network (sizes of the circles are balanced for each network). The nature of each correlation (whether is positive of negative correlation) is described in Supplemental Figure 4.

(B) Network densities of each bootstrap were calculated for each study group and timepoint as described in the Methods section. Data were compared using the Kruskal-Wallis test with Dunn's multiple comparisons *ad hoc* test.

(C) At each timepoint, the top three markers with the highest number of statistically significant correlations were selected. The number of connections for each marker were compared between the study timepoints using the Freedman's matched pairs test with Dunn's multiple comparisons *ad hoc* test or non-parametric linear trend analysis. P-values were adjusted for multiple comparisons using the Holm-Bonferroni method.

(D) Area under the curves (AUC) values were calculated for each marker shown in (C).

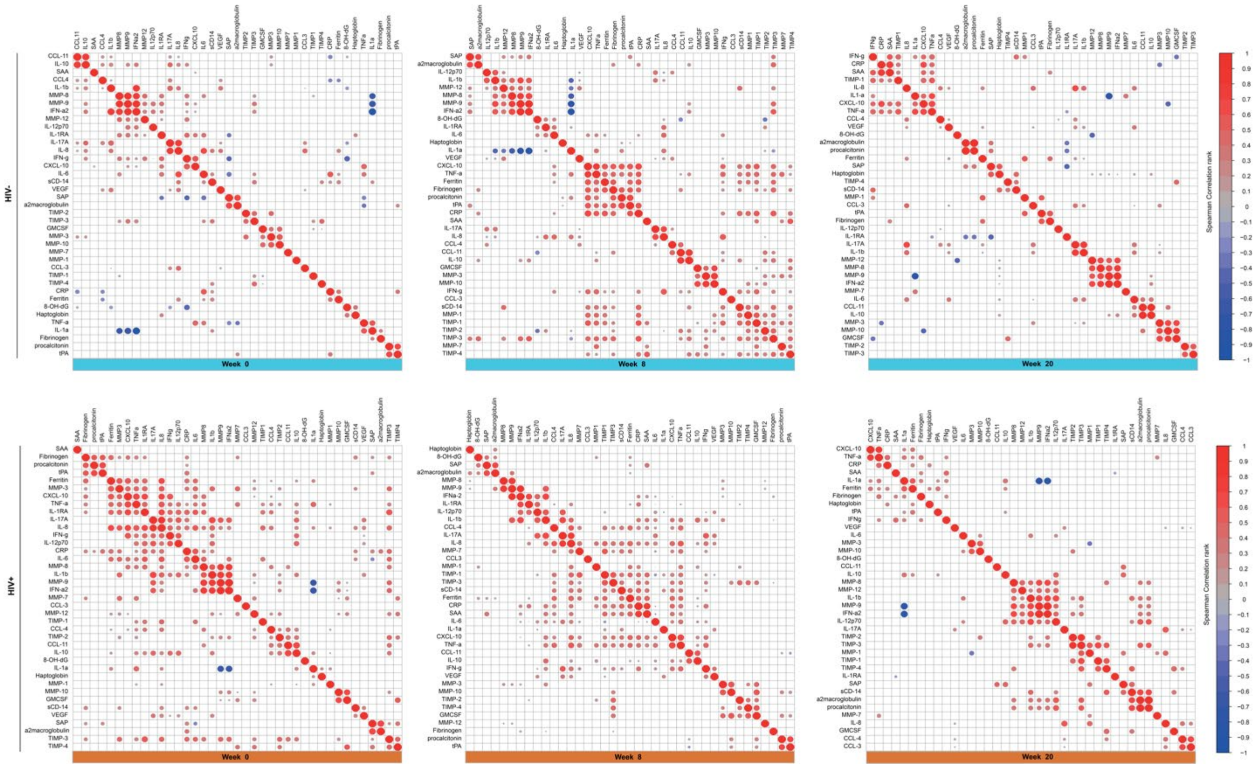

Supplementary Figure 6. Heatmap of the Spearman rank coefficient values of the correlations between plasma inflammatory markers in the HIV infected and – uninfected subgroups of the discovery cohort at weeks 0, 8 and 20. Correlation matrices were built for the indicated clinical group and timepoint and were submitted to 100x bootstrap. The graphs show correlations that persisted with a significant p-value (false discovery rate of 5%) in at least 80 times out of 100 bootstraps. Red circles indicate positive correlations whereas blue circles denote negative correlations. Size of the circles is proportional to the strength of the correlation.

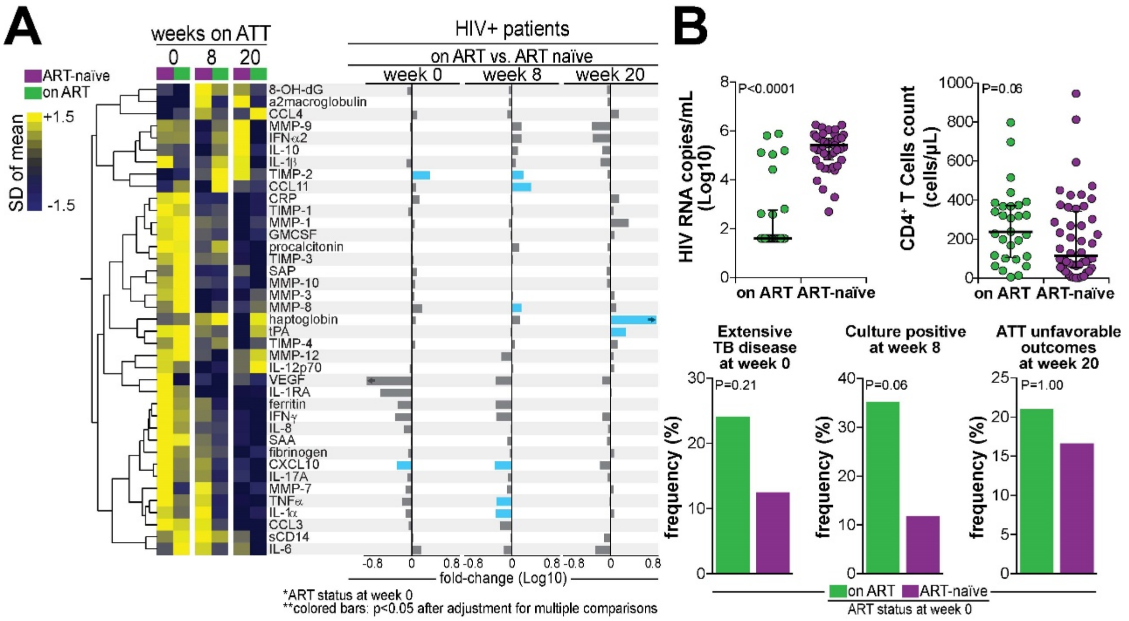

Supplementary Figure 7. Comprehensive analyses of differences in levels of inflammatory markers as well as clinical and virologic parameters in HIV-1-infected patients with pulmonary TB stratified according to ART status at various timepoints during antitubercular therapy.

(A) Hierarchical cluster analysis using z-scored values of each parameter (Ward's method) was employed to depict the overall biomarker expression profile in the HIV-1 infected individuals at the different timepoints of antitubercular therapy (ATT) and stratified according to ART status at the study baseline. Fold-variation values (on ART vs. ART naïve) were calculated and statistically significant differences of several biomarkers are highlighted in blue.

(B) Upper panel shows comparisons in HIV-1 RNA levels in plasma and CD4+ T-cell counts between individuals on ART and those who were ART naïve at the study enrollment. Data were compared using the Mann-Whitney *U* test. Lower panels show frequency of extensive TB disease, positive TB cultures at week eight of ATT and ATT-associated unfavorable outcomes between the groups of patients on ART or ART-naïve at study enrollment. Data were compared using the Fisher's exact test.

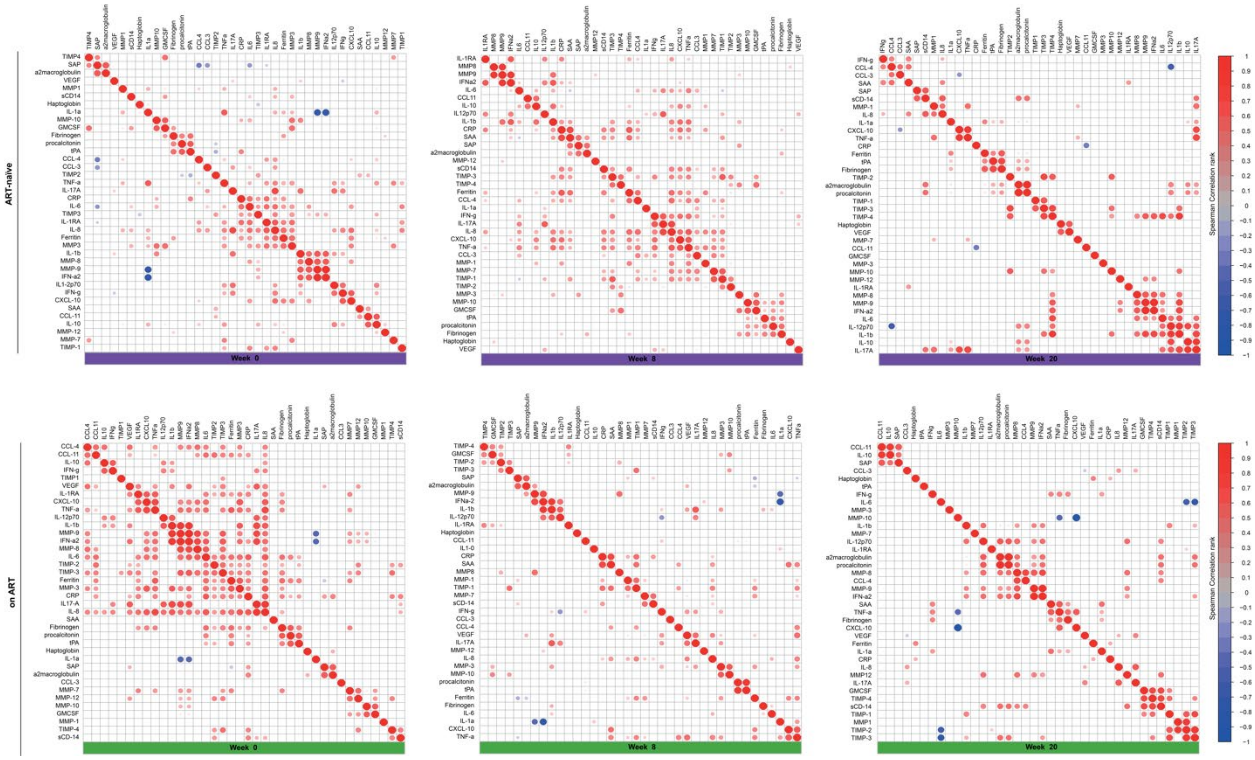

Supplementary Figure 8. Heatmap of the Spearman rank coefficient values of the correlations between plasma inflammatory markers in the ART naïve and established, HIV co-infected participants of the discovery cohort at weeks 0, 8 and 20. Correlation matrices were built for the indicated clinical group and timepoint and were submitted to 100x bootstrap. The graphs show correlations that persisted with a significant p-value (false discovery rate of 5%) in at least 80 times out of 100 bootstraps. Red circles indicate positive correlations whereas blue circles denote negative correlations. Size of the circles is proportional to the strength of the correlation.

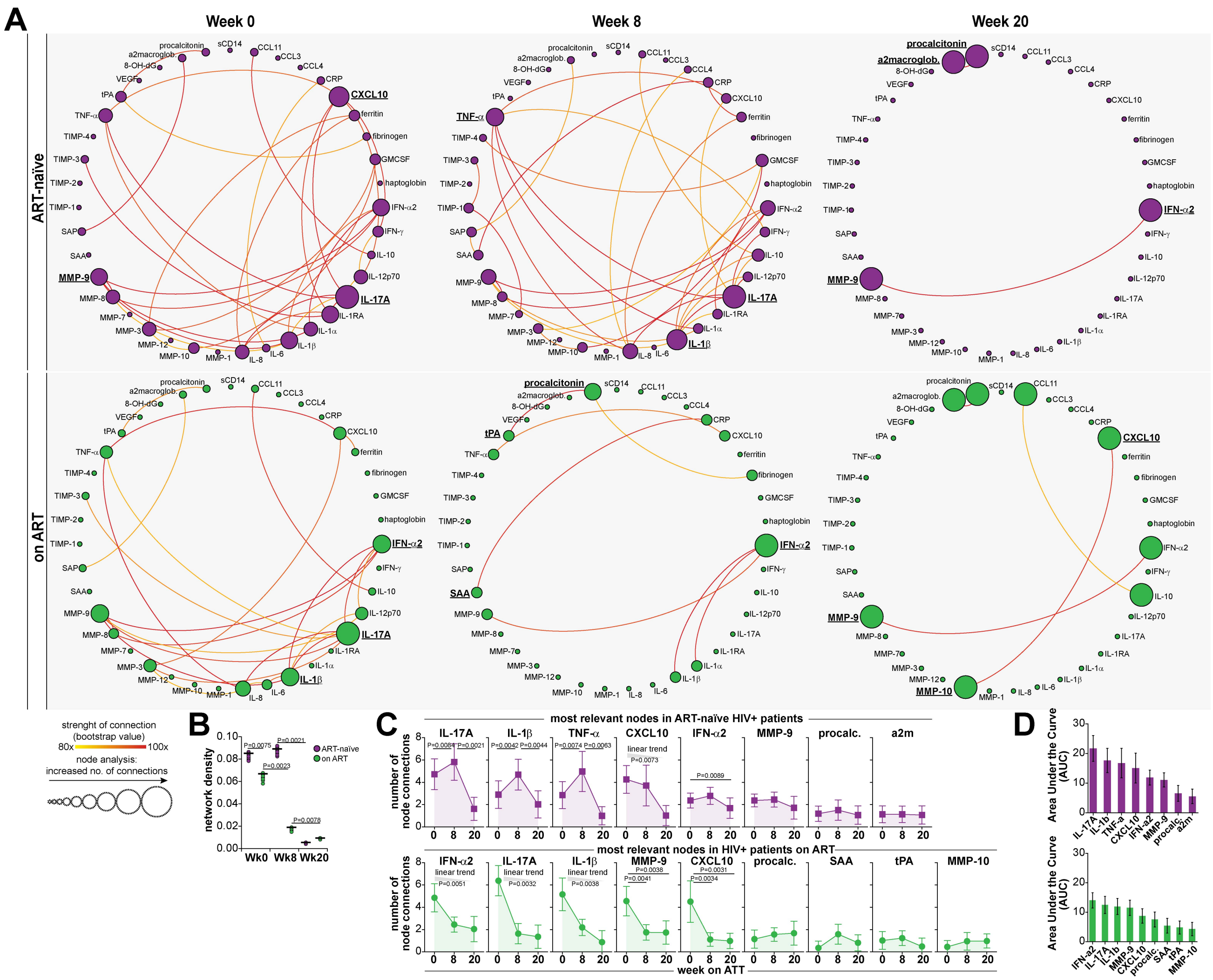

Supplementary Figure 9. Inflammatory signatures of plasma markers in pulmonary HIV-1-TB patients according to ART status at various timepoints during antitubercular therapy.

(A) Network analysis of the biomarker correlation matrices was performed with bootstrapping (100x). Relationships which remained statistically significant in at least 80 out of 100 bootstraps were plotted as connecting lines. Each node represents a different plasma parameter. The size of each circle is proportional to the number of significant correlations involving such node in each network (sizes of the circles are balanced for each network). The nature of each correlation (whether positive or negative) is described in Supplemental Figure 6.

(B) Network densities of each bootstrap were calculated for each study group and timepoint as described in the Methods section. Data were compared using the Kruskal-Wallis test with Dunn's multiple comparisons *ad hoc* test.

(C) At each timepoint, the top three markers with the highest number of statistically significant correlations were selected. The number of connections for each marker were compared between the study timepoints using the Freedman's matched pairs test with Dunn's multiple comparisons *ad hoc* test or non-parametric linear trend analysis. P-values were adjusted for multiple comparisons using the Holm-Bonferroni method.

(D) Area under the curves (AUC) values were calculated for each marker shown in (C).

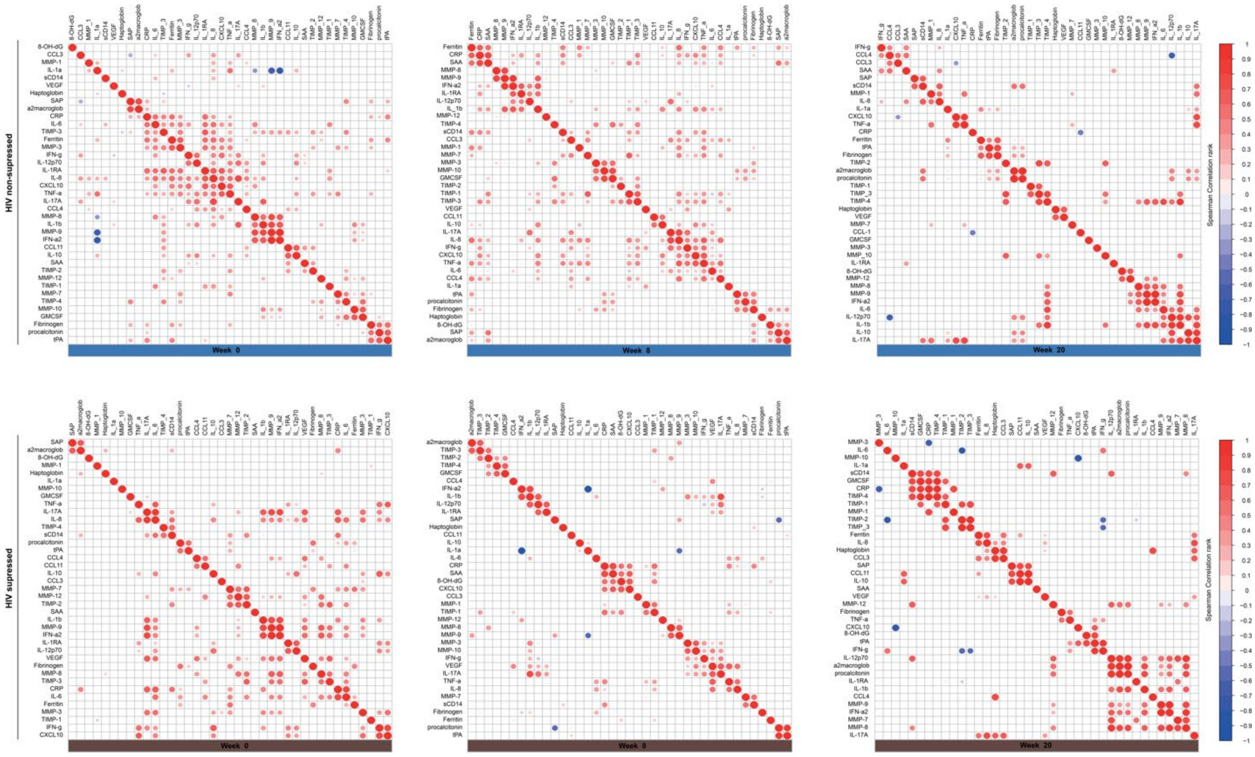

Supplementary Figure 10. Heatmap of the Spearman rank coefficient values of the correlations between plasma inflammatory markers at weeks 0, 8 and 20 in HIV co-infected participants of the discovery cohort with suppressed versus unsuppressed viral load. Correlation matrices were built for the indicated clinical group and timepoint and were submitted to 100x bootstrap. The graphs show correlations that persisted with a significant p-value (false discovery rate of 5%) in at least 80 times out of 100 bootstraps. Red circles indicate positive correlations whereas blue circles denote negative correlations. Size of the circles is proportional to the strength of the correlation.

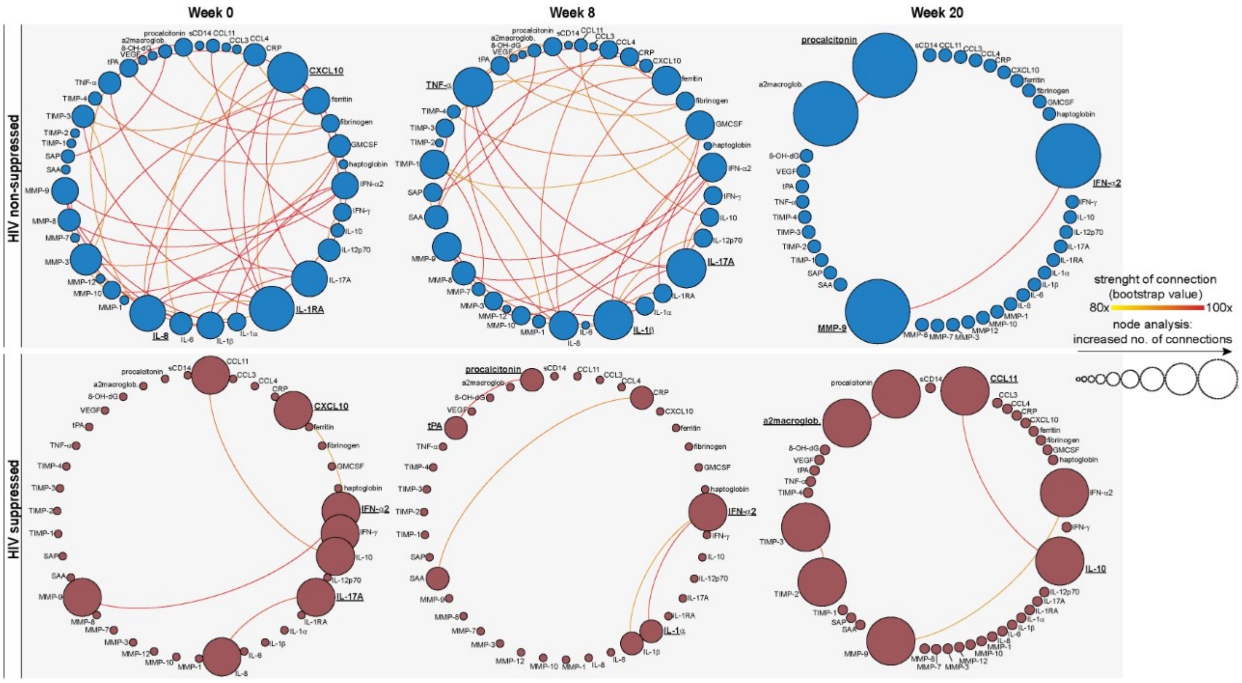

Supplementary Figure 11. Inflammatory signatures of plasma markers in pulmonary HIV-1-TB co-infected patients stratified by HIV-1 viral suppression during ATT.

Network analysis of the biomarker correlation matrices was performed with bootstrapping (100x). Relationships which remained statistically significant in at least 80 out of 100 bootstraps were plotted as connecting lines. Each node represents a different plasma parameter. The size of each circle is proportional to the number of significant correlations involving such node in each network (sizes of the circles are balanced for each network). The nature of each correlation (whether positive or negative) is described in Supplementary Figure 10.

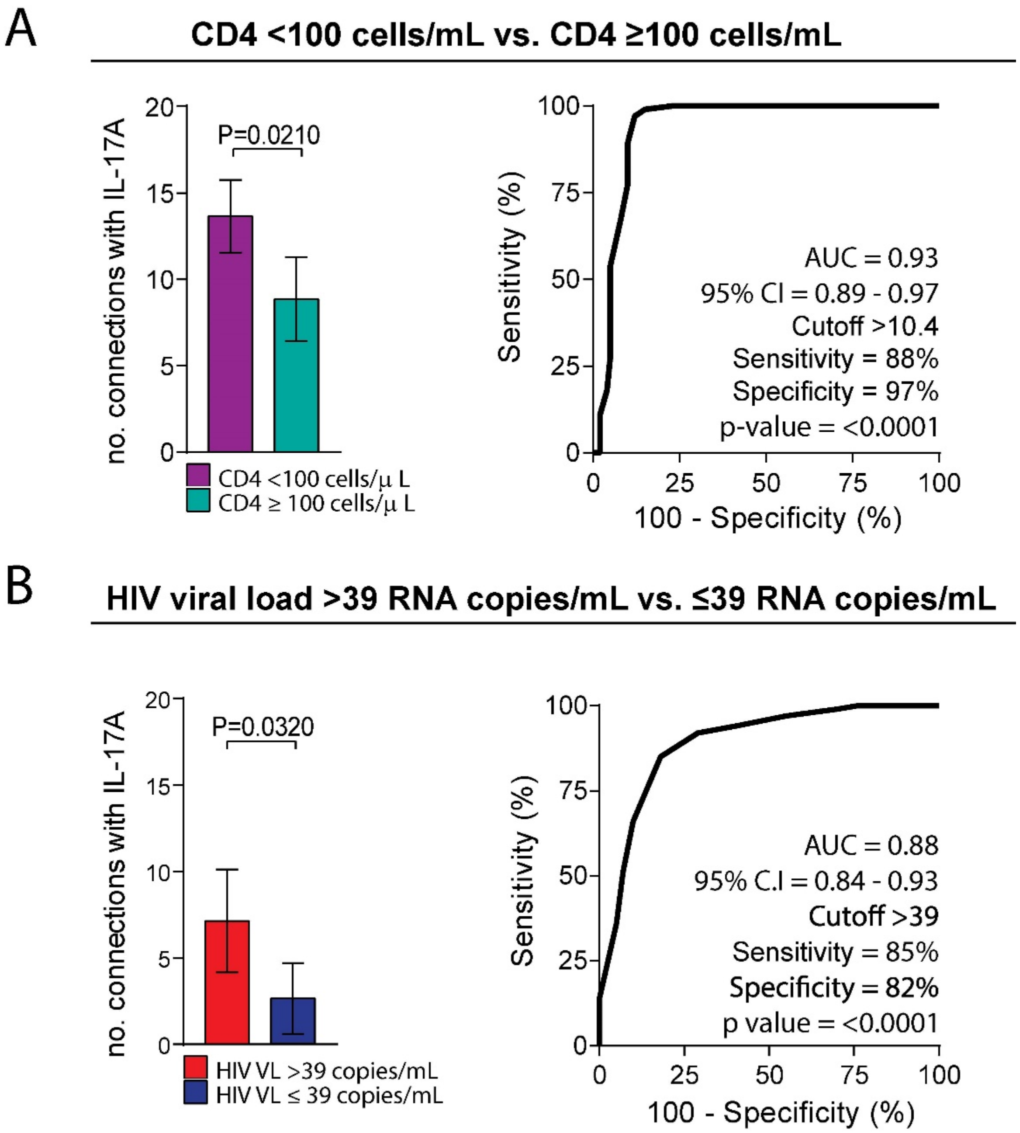

Supplementary Figure 12. Inflammatory signatures of plasma markers in outpatients with HIV-1 infection and no evidence of active TB disease.

(A) Number of connections involving IL-17A were compared in participants with CD4 counts <100 cells/μl and those with a CD4 count ≥ 100 cells/μl.

(B) Number of connections involving IL-17A were compared in participants with detectable HIV-1 viral loads (>39 copies/ml) and those with viral loads below the limit of detection (≤39 copies/ml).

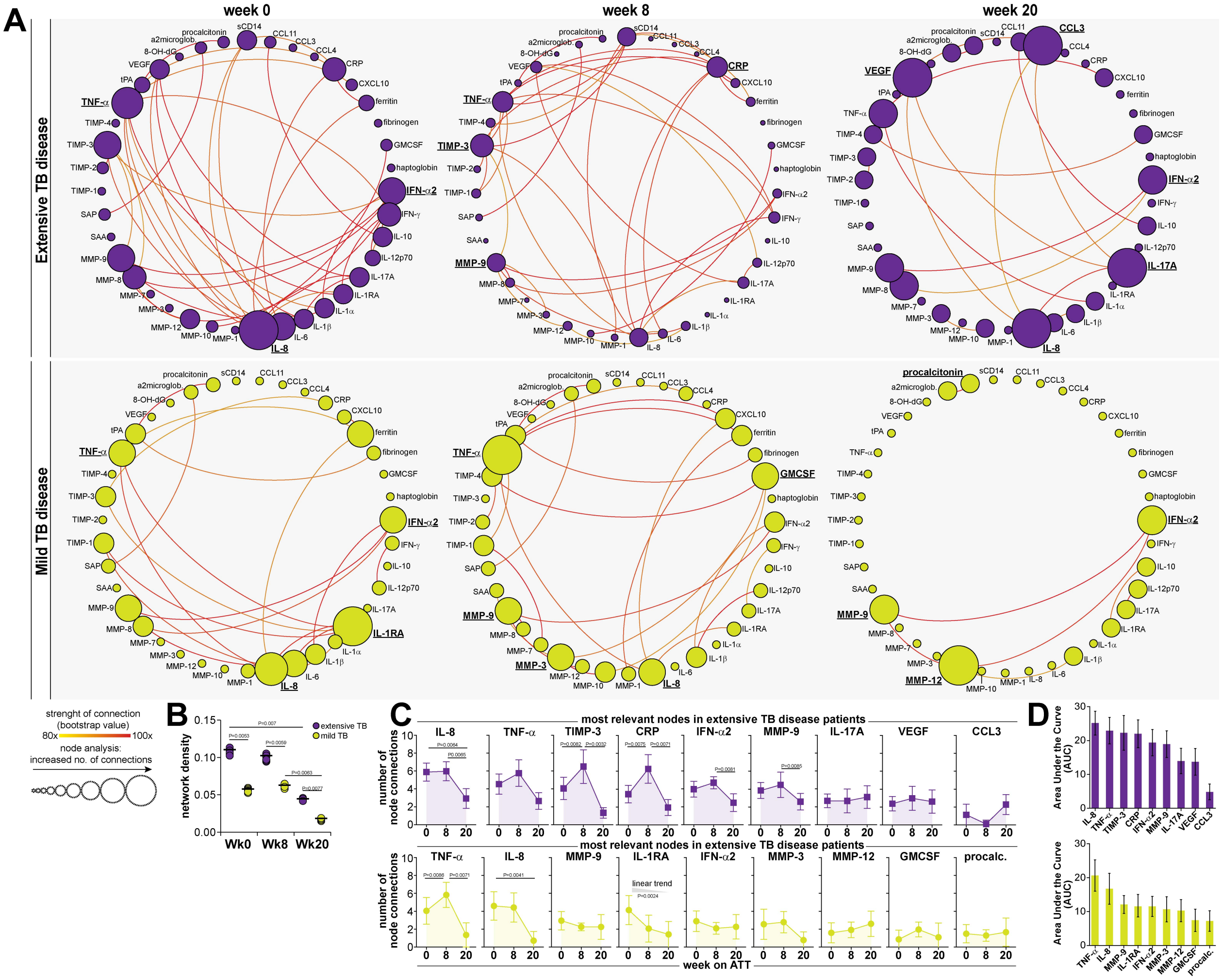

Supplementary Figure 13. Inflammatory signatures of plasma markers in HIV-1 uninfected pulmonary TB patients with radiographic evidence of mild versus extensive TB disease.

(A) Network analysis of the biomarker correlation matrices was performed with bootstrapping (100x). Relationships which remained statistically significant in at least 80 out of 100 bootstraps were plotted as connecting lines. Each node represents a different plasma parameter. The size of each circle is proportional to the number of significant correlations involving such node (sizes of the circles are balanced for each network).

(B) Network densities of each bootstrap were calculated for each study group and timepoint as described in the Methods section. Data were compared using the Kruskal-Wallis test with Dunn's multiplecomparisons ad hoc test.

(C) At each timepoint, the top three markers with the highest number of statistically significant correlations were selected. The number of connections for each marker were compared between the studytimepoints using the Freedman's matched pairs test with Dunn's multiple comparisons ad hoc test or non-parametric linear trend analysis. P-values were adjusted for multiple comparisons using the Holm-Bonferroni method.

(D) Area under the curves (AUC) values were calculated for each marker shown in (C).

References

1. Oliveira-de-Souza D, Vinhaes CL, Arriaga MB, et al. Aging increases the systemic molecular degree of inflammatory perturbation in patients with tuberculosis. Sci Rep. 2020;**10**(1):11358.
2. Vinhaes CL, Oliveira-de-Souza D, Silveira-Mattos PS, et al. Changes in inflammatory protein and lipid mediator profiles persist after antitubercular treatment of pulmonary and extrapulmonary tuberculosis: A prospective cohort study. Cytokine. 2019;**123**:154759.
3. Vinhaes CL, Sheikh V, de-Souza DO, et al. An inflammatory composite score predicts mycobacterial IRIS in people with HIV and severe lymphopenia: A prospective international cohort study. J Infect Dis. 2020.
4. Oliveira-de-Souza D, Vinhaes CL, Arriaga MB, et al. Molecular degree of perturbation of plasma inflammatory markers associated with tuberculosis reveals distinct disease profiles between Indian and Chinese populations. Sci Rep. 2019;**9**(1):8002.
5. Vinhaes CL, Arriaga MB, de Almeida BL, et al. Newborns With Zika Virus-Associated Microcephaly Exhibit Marked Systemic Inflammatory Imbalance. J Infect Dis. 2020;**222**(4):670-80.
6. Vinhaes CL, Cruz LAB, Menezes RC, et al. Chronic Hepatitis B Infection Is Associated with Increased Molecular Degree of Inflammatory Perturbation in Peripheral Blood. Viruses. 2020;**12**(8).
7. Andrade BB, Singh A, Narendran G, et al. Mycobacterial antigen driven activation of CD14++CD16- monocytes is a predictor of tuberculosis-associated immune reconstitution inflammatory syndrome. PLoS Pathog. 2014;**10**(10):e1004433.
8. Andrade VMB, de Santana MLP, Fukutani KF, et al. Multidimensional Analysis of Food Consumption Reveals a Unique Dietary Profile Associated with Overweight and Obesity in Adolescents. Nutrients. 2019;**11**(8).
9. Cruz LAB, Moraes MOA, Queiroga-Barros MR, Fukutani KF, Barral-Netto M, Andrade BB. Chronic hepatitis B virus infection drives changes in systemic immune activation profile in patients coinfected with Plasmodium vivax malaria. PLoS Negl Trop Dis. 2019;**13**(6):e0007535.
10. Barretto JR, Boa-Sorte N, Vinhaes CL, et al. Heightened Plasma Levels of Transforming Growth Factor Beta (TGF-beta) and Increased Degree of Systemic Biochemical Perturbation Characterizes Hepatic Steatosis in Overweight Pediatric Patients: A Cross-Sectional Study. Nutrients. 2020;**12**(6).
11. Schutz C, Barr D, Andrade BB, et al. Clinical, microbiologic, and immunologic determinants of mortality in hospitalized patients with HIV-associated tuberculosis: A prospective cohort study. PLoS Med. 2019;**16**(7):e1002840.

Clinical Protocol

Frequency and determinants of acquired drug resistance in individuals receiving treatment for *M. tuberculosis*

Investigators

Prof Robert J Wilkinson[1,2]
Prof Helen McIlleron[1]
Prof Graeme Meinjties[1,2]
Prof Sebastien Gagneux[3]
Dr. Rachel Lai[4]
Prof Douglas Young[4]

Dr Neesha Rockwood[1,2] (lead investigator)

Institutions

[1]University of Cape Town, Cape Town, South Africa
[2]Imperial College, London, UK
[3]Swiss Tropical and Public Health Institute, Basel, Switzerland
[4] National Institute of Medical Research, London, UK

Contents

Summary ... 4

Background ... 5

Rationale and scope of study ... 13

Hypotheses ... 18

Aims ... 19

Methodology ... 20

Study population, clinical site and treatment regimens ... 20

Inclusion criteria: ... 21

Further exclusion criteria for Group A only (if any of these apply, patient will be re-allocated to Group B) ... 21

Procedures ... 22

Screening visit- only for patients who are being started on TB treatment without microbiologically confirmed diagnosis of MTB ... 22

Study visit ABC1 -Group A, B and C ... 23

Study visit A1 -Group A only ... 25

Study visit ABC2- Group A, B and C ... 26

Study visit A2- Group A ... 26

Study visit BC1- group B and C ... 27

Study visit ABC3- group A, B and C ... 27

Study visit BC2- group B and C ... 28

Proposed collaborative multi-regional TB genomic study ... 29

sRNA sub-study ... 29

Statistical Analyses ... 31

Sample size calculation ... 32

Confidentiality ... 33

Social/Economic & Ethical implications 34

Potential adverse effects for participants 34

Financial implications 35

Time expected for participation 35

Benefits to participants/ patients 36

Potential adverse effects for researchers 36

Ethical statements 37

Informed consent process 37

Data entry and results 37

Outcome 38

Timetable and Milestones 39

Budget 39

Key personnel 40

Appendix 1 Study Flow Chart (PTO as continued on next page) 42

Appendix 2 Budget summary 44

References 45

Summary

Tuberculosis (TB) is the largest cause of death in HIV-infected people. An increasing problem is the development of drug resistant strains of *M. tuberculosis* (MTB). Drug resistance is thought to arise because of poorly supervised treatment and poor adherence. Recent evidence suggests however, that even when treatment is apparently optimal, inadequate drug concentrations may occur in the tissues of the body. Thus, drug resistance may develop during therapy even in those in whom treatment is thought adequate. Acquired drug resistance (ADR) is the development, fixation and amplification of mutations conferring resistance under drug pressure over the course of treatment.

We will sequentially recruit and follow up patients who present to the TB clinic/inpatient facilities and commence treatment for GeneXpert confirmed pulmonary TB. The patients will be predominantly recruited from an outpatient setting and will be a mix of new and retreatment cases. HIV infection rates are likely to be 50-75%, reflecting high HIV-TB co-infection rates in Khayelitsha. We aim to ascertain the frequency of acquired drug resistance (ADR).We will collect sequential sputa at baseline, 2 and 5 months of follow up. We will sequence entire pathogen genomes and will analyse these to confirm ADR and study microevolution of drug resistance. The majority of patients are likely to be rifampicin sensitive at baseline. We aim to ascertain the frequency of ADR over follow up. We will also follow up cases of baseline multi drug resistant (MDR) TB and will be able to ascertain development of amplified drug resistance.
We will also contribute MTB strains to a multicentre genomic study. It aims to study the pathway of accumulation of drug-resistance-associated mutations in HIV-infected and HIV-uninfected patients, and to determine the levels of phenotypic drug resistance associated with particular mutations in HIV-infected versus non-infected patients and across different genetic backgrounds (i.e. phylogenetic lineages) of (MTB).

In the sRNA sub-study, to better understand the phenotype of MTB and the transcriptomic changes induced by antibiotic treatment, we plan to study MTB in the sputum collected from patients displaying active TB disease, with or without concurrent HIV co-infection, during the course of anti-TB treatment. We aim to follow up a subgroup of smear positive pulmonary TB patients over 8 weeks of chemotherapy and collect paired blood and sputum samples at times points baseline, 1-2, 4 and 8 weeks follow up.

There is inconclusive data on the role of HIV co-infection in the evolution of drug resistant MTB strains. We will collect data on determinants of ADR, focussing on HIV co-infection and severity of immunosuppression secondary to HIV. We will also collect data on time to detection of MTB in liquid cultures and smear grade, as a surrogate of baseline bacterial load and baseline radiological degree of severity. We will also take a detailed history of previous TB and previous anti-TB drug regimens and screen for adherence to therapy

during treatment follow up. We will identify individuals who develop ADR during anti-TB therapy and compare pharmacokinetic (PK) profiles (via intensive sampling methods) with matched controls at 2 months of follow up. We hypothesize that TB drug concentrations will be significantly lower in cases of ADR compared with controls.

Low plasma concentrations of anti-TB drugs have been associated with longer treatment response times, treatment failure, and the emergence of drug resistance. A particular dose does not lead to a specific time-concentration profile in all patients. There is considerable interindividual (between individuals) and interoccasional (in the same individual at different time points) variability in TB PK due to multiple factors, both known and unknown. Exposure to anti-TB drugs is known to change during treatment. The significance of change in TB PK in HIV co-infected individuals pre and post antiretroviral (ART) therapy is unclear and will also be addressed in our study. We will explore determinants of change in TB PK in a cohort of HIV co-infected individuals undergoing therapy for pulmonary TB, focussing on individuals with CD4 ≤100 cells/mm^3. These severely immunocompromised individuals are likely to have greatest physiological change post immune reconstitution secondary to ART and hence may potentially have significant change in TB PK.

Background

Drug resistant TB

Globally, in 2009, the rate of treatment completion or cure for new registered cases of sputum smear-positive pulmonary TB was 87%. This exceeded the WHO target of 85%. However, these treatment success rates varied widely and were as low as 66% in the European region. (1)In South Africa, this figure was 71.9%. (2)Rates of treatment success are lower in retreatment cases. In sub-Saharan Africa, there has been reduced sputum culture conversion at 2months compared with non-African countries.(3)(4)(5)

Despite the WHO implemented directly observed therapy short course (DOTS) programme in the 1990s, drug resistant TB is an increasing global health problem, particularly in ‘hot spots’ such as Sub-Saharan Africa, Eastern Europe, the Russian Federation, central Asia, India and China.(6)Multi drug resistant (MDR) TB has been described since the late 1980s. MDR TB is defined by the WHO as resistance to the first line drugs rifampicin and isoniazid. Loss of efficacy of these drugs significantly compromises a regimen and necessitates addition of multiple other second line anti-TB drugs. Extensively drug resistant (XDR) TB is defined by the WHO as infection with *Mycobacterium tuberculosis* (MTB) which is resistant to rifampicin and isoniazid, a fluoroquinolone and at least one second-line injectable drug (capreomycin, kanamycin and amikacin).(7) Patients with MDR and XDR TB require intensive phase treatment with

5-7 drugs, including co-administration of an injectable drug, for a minimum of 6 months and 18 months continuation phase treatment after sputum culture conversion.(8)This is in contrast to the recommended 6-8 months for drug susceptible TB. Second line drugs are associated with significantly greater toxicity. (9)(10)(11)(12)This is particularly pertinent in HIV co-infected individuals who may have added side effects from their anti-retroviral therapy (ART).(13)MDR and XDR TB use up a disproportionate percentage of overall national TB budgets due to costs of hospitalisation and second line drugs (Pooran et al. *What is the cost of diagnosis and management of drug resistant tuberculosis in South Africa?* Submitted to PLoS ONE).

Globally there were an estimated 650,000 new cases of MDR TB in 2010 (14)and 150,000 deaths due to MDR TB in 2008.(6) XDR TB has been reported in 58 countries globally and in 8 of these countries, more than 10% of MDR TB cases have met criteria for classification as XDR TB. (1) In drug resistant TB, cure and completion rates are lower, with higher mortality rates. (15)(16,17)(18)(19)

Pulmonary TB presents with dynamic lesions containing subpopulations of MTB differing in metabolic activity. (20)A MTB isolate is defined as drug resistant if it has a minimum inhibitory concentration (MIC) which prevents it from being effectively killed by drug concentrations achieved by 90% of a population given a specific dose. (21) Isoniazid and rifampicin comprise the cornerstone of the DOTS regimen due to their potent bactericidal activity. (22)(23).Isoniazid is responsible for about 95% of the initial bactericidal activity during the first 2 days of combined therapy. (24) Rifampicin then takes over as the chief active drug against MTB in the exponential growth phase. It also possesses activity against non-replicating persistent bacilli.(25)Pyrazinamide is bactericidal against intracellular intermediately active MTB in macrophages.(26) Ethambutol has bacteriostatic activity and protects against development of rifampicin resistance. These drugs have a post antibiotic effect of a persisting antimicrobial effect even after serum concentrations fall below the MIC. For the above reasons, multidrug therapy is important, particularly in the context of multibacillary disease e.g. in cavitatory pulmonary TB, to effectively kill MTB and prevent amplification of resistant sub-populations. At least 6 months of therapy is believed necessary to eradicate persistent subpopulations of less metabolically active bacteria, thereby preventing disease recurrence. (27)

Drug resistant TB is primarily a man-made phenomenon where MTB has been inadequately treated with regimens containing only one or two active drugs, leading to inadequate drug pressure and allowing the selection of bacteria with mutations conferring resistance to anti-TB drugs. In places where there was a lack of laboratory infrastructure for culture and drug sensitivity testing (DST), empirical antibiotic treatment is likely to have fuelled the emergence of drug resistant TB. This is particularly pertinent in cases where patients who had previously failed to culture convert were being retreated

for TB with the addition of only a single additional drug (streptomycin) to their regimen. Traditionally, the development of resistance has been believed to be linked to poor adherence to treatment. Some important risk factors for poor adherence and early defaulting from prescribed regimens include alcohol and drug abuse and concurrent socio-demographic issues such as homelessness. These in turn have been found to be associated with poor treatment outcomes.(28)(29)(30)(9,12) Increased pill burden, particularly in the context of HIV co-infection and adverse drug events are also associated with poorer adherence.

The spontaneous mutation rate conferring resistance to rifampicin is estimated to be 10^8 and for isoniazid it is 10^6.(31)Hence, it is possible that a tuberculous cavity harbouring an initial baseline bacterial load of $10^7$to 10^9,may contain a few bacteria (<1000) with mutations conferring resistance to TB drugs. However, less than 1% of the overall population have resistance, the phenotypic DST will be reported as sensitive and the minimum inhibitory concentration will be within the range for wild type bacteria. For purposes of this proposal, acquired drug resistance (ADR) encompasses both the development and fixation within the clonal population of de novo mutations conferring resistance and the selection and amplification of pre-existing resistant mutants in the original bacterial population under drug pressure over the course of treatment. Molecular techniques have been successfully used in studies to differentiate exogenous re-infection from reactivation of disease, to identify simultaneous dual infection with mixed strains, genetically homogenous drug susceptible and drug resistant strains in the same isolate (heteroresistance) and ADR. The frequency of ADR varies widely in published literature.(32)(33)(34)(35)(36) Overall, results have been limited by retrospective study designs, small sample sizes, different cohort selections, missing culture results and drug sensitivity testing (DST) incomplete MTB strain genotyping and limited pharmacokinetic (PK) studies.

Study	Location	Acquired/amplified drug resistance (ADR)	Other relevant information
Pepper et al (33)	South Africa	8/73 (11%) of those being admitted as inpatients for clinical deterioration acquired new RIF resistance	Sample size limited as many in deteriorator cohort had missing drug sensitivities
Cox et al(36)	Turkmenistan Uzbekistan	19/382 (5%) acquired new or further resistance. 1.2% ADR with initial pansusceptibility or monoresistance and 17% with initial polyresistance	Standardised short course therapy irrespective of baseline drug sensitivities
Seung et al(34)	Tomsk Oblast, Russian Federation	30/1681 (1.8%) acquired drug resistance. 17/24 (70.8%) treatment failures with initial INH/RIF monoresistance acquired MDR 13/31(41.9%) treatment failures with	Standardised short course therapy given regardless of baseline drug sensitivities

		initial pan-sensitivity acquired MDR	
Temple et al (32)	Uganda	13/250 (5.2%) acquired drug resistance. 6/13 (46%) had baseline MDR	WHO regimen 2 given
Yoshiyama et al(35)	Thailand	18/2032 (0.9%) acquired drug resistance. 4/31(13%) of re-registered patients with initial pan-sensitivity acquired resistance	Majority of ADR cases HIV infected
Bonnet et al (37)	Georgia	7/326 (2.1%) acquired drug resistance, all had baseline MDR TB or polyresistance	Individualized TB treatment regimens
Buu et al (38)<br><br>Quy et al (39)	Vietnam<br><br>Vietnam	3/1106 (0.3%) acquired drug resistance<br>15/2901 (0.52%) acquired MDR TB	2/3 amplified baseline MDR<br>13/15 had baseline resistance
Porco et al (40)	USA	100/33725 (0.3%) patients acquired MDR TB, 27/3039 (0.9%) acquired drug resistance if initially isoniazid-resistant	Repeat DST results only available for 1792 patients

A summary of results from studies of acquired and amplified drug resistance MDR- multi drug resistant RIF- rifampicin INH- isoniazid STREP- streptomycin

In certain settings such as nosocomial outbreaks, and particularly when drug resistant strains are of high virulence, cases of MDR TB and XDR TB can be directly transmitted as opposed to ADR. Underlying contributory factors include poor infection control strategies, HIV co-infection and a high prevalence of untreated/inappropriately treated MDR and XDR TB in the community.(41)(42)

Hence, it is important to understand the evolution of ADR and to prevent the spread of highly adaptive drug resistant MTB strains.

Population genomics of drug resistant TB

Strategies for controlling drug resistance in Mtb include drug susceptibility testing, surveillance, as well as ensuring completion of an adequate treatment regimen and patient follow-up. However, bacterial factors may also contribute to the global emergence and spread of drug-resistant TB. In particular, epistatic interactions between drug resistance mutations, compensatory mutations and different strain genetic backgrounds could play a role in this context (43). While many drug resistance-conferring mutations in MTB lead to a reduction in bacterial fitness, some are associated with no fitness cost and successful transmission (44-46). Furthermore, when analyzing clinical strains that acquired drug-resistance during TB treatment, some of these strains had no fitness cost compared to the initial drug-susceptible isolate, suggesting that compensatory evolution mitigated initial fitness defects. In support of this view, compensatory mutations were identified in the RNA polymerase of rifampicin-resistant M. tuberculosis strains and were associated with a high competitive fitness (47). Moreover, these mutations occurred in more than 30% of MDR clinical strains from countries with a high MDR TB burden, suggesting that these mutations contribute to the success of MDR strains. A recent study from South Africa has shown that these compensatory mutations were also associated with increased transmission (48). Given the phenomenon of compensatory evolution , it is interesting to speculate that HIV-infected TB patients could serve as a 'breeding ground' for highly compensated drug-resistant strains, with an increased capacity to spread in the general population.

In addition to the relevance of the particular drug resistance-conferring mutation and the potential for compensation, the strain genetic background has also been shown to impact drug-resistant M. tuberculosis. For example, the strain genetic background has been shown to modulate the level of resistance conferred by particular drug resistance mutations (46). Moreover, the “Beijing” lineage of M. tuberculosis has repeatedly been associated with drug resistance (45), suggesting that this strain lineage might be 'pre-

adapted' to drug resistance. Importantly, the “Beijing” strain lineage has also been associated with HIV (49,50) and is emerging in South Africa (51). The “Beijing” lineage belongs to Lineage 2, one of seven main phylogenetic lineages defined in the human-associated MTB complex. These lineages are associated with different geographic regions and human populations. Hence, it is possible that this global phylogeography of MTB complex could impact the emergence and spread of drug resistant TB (52).

The role of pharmacokinetics/pharmacodynamics in development of drug resistant TB

Low plasma concentrations of anti-TB drugs have been associated with longer treatment response times, treatment failure, and the emergence of drug resistance. (53)(54)(55)(56)

In TB, microbial killing, resistance suppression and post-antibiotic effect of anti-TB therapy are concentration dependent. The findings of studies support the hypothesis that it is the ratio of maximum concentration C_{max} to MIC that is the chief pharmacokinetic (PK) determinant of the development MTB populations resistant to rifampicin and isoniazid. (25)'(57) Said another way, if peak concentrations of anti-TB drugs are increased, for example via an increase in dose, the persisting ‘tolerant’ MTB populations, are more likely to be eliminated during chronic TB treatment. In time-kill *in vitro* studies and in a murine aerosol model, area under curve (AUC) to MIC was closely related with bactericidal activity. (58)' (25,59)(60)For pyrazinamide and ethambutol, time concentration of drug remained above MIC (%T_{MIC}) was an important determinant of drug resistance. (21)(61,62)

Pulmonary PK and pharmacodynamic data is scarce. There is significant heterogeneity, both structurally and biochemically, of pulmonary lesions ranging from solid granulomas to cavities surrounded by a fibrotic wall. Differential lesion specific penetration is likely to be important in TB. (63)The majority of metabolically active replicating bacteria are extracellular within liquefied caseating matter. Semi-dormant bacteria are more likely to be intracellular.(27)A nonparametric PK model which described the plasma and pulmonary concentrations of RIF in 34 human volunteers, along with Monte Carlo simulations of 10,000 subjects showed that concentrations of rifampicin in epithelial lining fluid (ELF) were slightly lower than in plasma and the C_{max}/MIC ratios of ≥175 for plasma and ELF were achieved in less than 50% of cases, even at the lowest MIC of 0.01 mg/liter. (64) In another study, concentrations of rifampicin post a single dose were above the MIC_{90} in the ELF, alveolar macrophages and bronchial biopsies. However, the assays were not carried out in individuals infected with TB and they had not received multiple doses to achieve a steady state of rifampicin concentrations (65). From the published data, it appears that a 600mg dose of rifampicin produces adequate bactericidal intracellular (alveolar macrophage) concentrations but inadequate extracellular (ELF) bactericidal

concentrations.(64),(65)Dartois *et al* used nonlinear mixed effects PK modelling to compare plasma, pulmonary and lesion specific TB drug penetration in rabbit models post single and multiple dosing. There was significantly reduced penetration of 4 anti-TB drugs in diseased tissue relative to plasma and also variable interlesional penetration within the same individual.(63)

Interoccasional variability is usually understood to be random i.e. there is no specific trend in the data over time. Ascertainment of population PK of rifampicin in South African pulmonary TB patients via semimechanistic models has shown interindividual variability of 52.8% for clearance and 43.4% for volume of distribution and an interoccasional variability of 22.5% for apparent clearance.(66)Of relevance, the PK analyses were done inpatients established on anti-TB treatment for between 10 and 60 days, in order to compensate for the expected time-dependent effect of autoinduction on rifampicin PK. (62)A population model for isoniazid PK in a South African population showed significant variability in absorption, primarily due to interoccasional variability. (67)A population model for pyrazinamide PK in a South African population showed 2 distinct subgroups with significant variation in absorption, volume of distribution and clearance.(68)

A particular dose does not lead to a specific time-concentration profile in all patients.(21) Variability in TB PK is due to multiple factors, both known and unknown. Known factors include sex (69,70), age (70) weight (69)(70) levels of plasma protein binding (71) , changes in intestinal permeability including malabsorption(72)(73)(74), food effects (75) and fixed dose versus single drug formulation. Formulation can affect how soon drug is absorbed (66). The activity of drugs such as pyrazinamide and aminoglycosides are particularly sensitive to changes in acidity.(24)Pharmacogenetics such as N-acetyltransferase-2 (NAT-2) and SLCO 1B1 status is likely to be an important determinant of interindividual variability of isoniazid and rifampicin respectively.(67)(56,69)(76)The proportion of isoniazid fast acetylators in South Africa has been estimated to be 60%. (77)In another study, the polymorphism SLCO1B1 rs4149032 ,which is associated with lower rifampicin concentrations, was found in 70% of the population.(78)

Studies have shown that patients dosed with either 450/480 mg rifampicin if <50 kg or 600 mg rifampicin if ≥50 kg (resulting in dose/kg of approximately 8-13 mg/kg) and where the MIC is estimated tobe 250 µg/L, the AUC/MIC should be around 184. (27),(79) However, in a significant proportion of patients, plasma concentrations are low on the dose/response curve. (80,81)(78) This in turn is due to factors such as rifampicin autoinduction. In simulations by Smythe et al, fewer than one third of patients reached minimum recommended C_{max} of 8 µg/ml at induced state post multiple 450 mg or 600 mg daily doses of rifampicin. (82)Pharmacogenetic variables are also important and therefore more significant in certain geographical regions, including sub-Saharan Africa.(56,78)(76)There also cases of low level phenotypic rifampicin resistant MTB

strains.(83)Higher doses of rifampicin would be needed in order to produce sufficient drug concentrations corresponding to the required MIC.(83)(84)(85) Further studies are required to evaluate the clinical outcomes of higher dosing +/- individualised dosing of TB treatment.

Through the use of hollow fibre models, Srivastava *et al* have measured the impact of omitted doses on development of drug resistant strains of TB. They found therapy failure only at non-adherence rates of greater than 60% and non-adherence did not lead to proportions of MTB strains that were resistant to rifampicin and isoniazid rising above 1%. Computer-aided clinical trial simulations were used to show that PK variability was sufficient to give rise to approximately 1% multidrug resistance, even in a scenario of 100% adherence. (86) It is also likely that the repercussions of non-adherence is most significant in those who have lower AUC and C_{max}.(87) PK mismatch is the variation in concentration of components of multidrug therapy due to asynchronous dosing frequency, varying drug half life and post antibiotic effect. It is hypothesised that PK mismatch could lead to periods of functional monotherapy allowing for selection of drug resistant populations. Srivastava *et al* showed that in hollow fibre models, 12 hour and 24 hour mismatched administration of rifampicin and isoniazid did not lead to drug resistance. One possible explanation of these results is that change in dosing frequency (and hence change in PK mismatch) can only affect the AUC. It has no effect on C_{max} which maybe the primary determinant of evolution of drug resistance to isoniazid and rifampicin.(88)These findings have yet to be confirmed in a clinical setting.

There is also evolving data showing alternative mechanisms of anti-TB drug resistance. For example, reverse transcriptase polymerase chain reaction (RT-PCR) has been used to show that the overexpression of certain efflux/transporter genes in the presence of anti-TB drugs can significantly decrease anti-TB drug levels and hence reduce drug susceptibility. This increased expression of drug transporters can be both by the host (78)and pathogen.(89),(90),(91)

Phenotypes and transcriptomes of mycobacteria during the course of TB treatment

Chemotherapy for TB usually consists of 2 months of intensive combined therapy of isoniazid, rifampin, ethambutol and pyrazinamide follows by an additional 4 months of isoniazid and rifampin. One reason for this extended treatment duration is the presence of persistent bacteria that are more tolerant to antibiotics. Failure to complete the treatment often results in bacterial load relapse and contributes to the rise of drug-resistant TB. The adaptation of TB to environmental changes during the course of infection is likely mediated by differential bacillary gene expression (92,93). There is growing evidence from studies in other bacteria that transcriptional control alone is not

solely responsible for gene regulation (94). A post-transcriptional regulatory network exists, that is dependent on small regulatory RNA (sRNA) molecules. sRNAs are short RNA transcripts (typically consisting of 50-250 nucleotides) which are not translated (94). These molecules work by either pairing with complementary mRNA or by interaction with proteins resulting in either degradation of mRNA or, enhancement or repression of translation (95) . With this ability to regulate protein expression, sRNAs enable bacteria to adapt to changing environments.

In bacteria other than TB, the role of sRNAs in both the stress response and also the regulation of virulence determinants, has been described (96-98). It is therefore reasonable to expect that sRNAs play a role in the pathogenesis of TB. A large number of sRNAs in TB have now been identified (99-101). Some of these have shown markedly differing levels in MTB under different in vitro growth phases and stress conditions (100). Microarray analysis on H37Rv cultured in antibiotics showed that persistent MTB is similar to their dormant counterpart with down-regulation of growth and energy metabolic and biosynethetic pathways (102). More contrasting phenotypes were reported in the mouse model and were suggested to depend on the tissue environment and immune response (103). In situ hybridization of a panel of selected genes of MTB in human lung granuloma tissues also showed that gene expression of the MTB is dependent on its local environment (104). More recently, microarray study on MTB isolated from sputum of patients suggested that MTB is of persistent phenotype, but the limited sample size and the lack of analytical significance rendered the finding inconclusive (105). Although the phenotypes of persistent bacteria have been studied using both in vitro culture system and mouse models, the impact of chemotherapy on MTB transcriptomes in human has yet to be investigated.

Rationale and scope of study

There is inconclusive data on the role of HIV co-infection in the evolution of drug resistant MTB strains. The data comes from epidemiological studies in different geographical populations. (106)(107)(108)(109)(110)(111)Proposed mechanisms for ADR include an overall increased bacterial burden in HIV co-infection leading to an increased spontaneous rate of mutation. In immunocompromised hosts, it is hypothesised that less fit drug resistant strains survive longer, allowing for development of compensatory mutations to restore fitness. (47)

Proposed collaborative genomic study

We have been approached to collaborate on an international TB genomic study which is entitled ‘Population genomics of drug-resistant tuberculosis’. It aims to study the pathway of accumulation of drug-resistance-associated mutations in HIV-infected and HIV-uninfected patients, and to determine the levels of phenotypic drug resistance associated with particular mutations in HIV-infected versus non-infected patients and across different genetic backgrounds (i.e. phylogenetic lineages) of MTB.

HIV co-infection may lead to important change in anti-TB drug pharmacodynamics/PK. There is conflicting data on the role of HIV co-infection in contributing to rifampicin PK.(66), (74), (112), (73), (113), (114,115), (116)(108)(55,117)(70) However, these data came from patients who had varying degrees of immunosuppression, co-morbidities predisposing to malabsorption and enteropathy, different dosing regimens and varying proportions of treatment with antiretroviral therapy (ART). In a semimechanistic PK-enzyme turnover model, HIV was associated with a 30% increase in apparent volume of distribution but its effect on rifampicin exposure was negligible. (82)HIV-infected individuals were found to have a 17% reduction in isoniazid clearance, possibly linked to reduction in NAT-2 activity. (67) HIV co-infection and low CD4 counts were independently associated with low median pyrazinamide concentrations. (55) HIV co-infection was associated with 15% reduction in bioavailability of ethambutol(118) and a low ethambutolC_{max}.(117)(70)

Immune reconstitution post ART may lead to variability in PK, for example via changes in gut permeability, although one would speculate levels would increase as absorption improved.(119)The clinical significance of change in exposure (C_{max} and AUC) toTB treatmentin HIV co-infected individuals pre and post ART treatment is unclear. As discussed, TB PK has considerable interindividual and interoccasional variability of both known and unknown aetiology.(66-68)

Saleri*et al*, showed an 83% increase in AUC at 10 weeks post commencement of ART compared with the pre-ART time point (this was 2weeks post commencement of TB treatment).(120) This was in a cohort of individuals from Burkina Faso with advanced immunosuppression (baseline median CD4 lymphocyte count of 73.5 cells/mm^3 (IQR 5-99)). Only 14 out of 16 individuals completed the study. Nevirapine was given to all patients along with a stavudine/lamivudine backbone. The increase in rifampicin concentrations is surprising in light of expected autoinduction of rifampicin at the later time point. Importantly, the results were not adjusted for covariates such as increase in weight during treatment. It should also be noted that none of the individuals reached rifampicin C_{max}>8 μg/ml.

McIlleron *et al*, examined variation in anti-TB drug PK in a cohort of 60 HIV co-infected patients from KwaZulu-Natal over 4 time points during the first 30 days post

commencement of TB treatment. This cohort included patients who commenced ART at 2weeks and a comparison group those who did not commence ART during TB treatment. The ART combination given to patients was different to Saleri *et al* and consisted of zidovudine/lamivudine/efavirenz. Multilevel linear mixed effect regression was used to quantify the effects of age, sex, change in weight, drug dose per kilogram of body weight at baseline, baseline CD4 lymphocyte count, drug dosing schedule, the concurrent first dose of ART (day 15) and the presence of steady-state ART (day 29) on the AUC of the respective anti-TB drugs measured on days 1, 8, 15 and 29. There was no independent effect of first dose ART or steady state ART on change in TB PK compared with day 1.(69) This study did not examine if ART had any significant effect on TB PK after 30 days.

Saleri *et al* and McIlleron *et al* present conflicting results on the effect of ART on TB PK over time presenting a dearth of knowledge in this field. Our study would aimto mechanistically separate the effect of ART on TB PK over time.It is likely that greatest PK variability will be seen in the most immunocompromised HIV co-infected individuals i.e. those with CD4 lymphocyte cell count less than or equal to 100 cells/mm^3. This will be the cohort in which we will address this question.

We cannot assume that all ART regimens have the same effect on TB PK. However, we will focus on patients commencing an ART combinations consisting of 2 nucleos(t)ides (in most tenofovir and lamivudine) and efavirenz, in order to reduce heterogeneity. Efavirenz based ART combinations are most widely used in HIV/TB co-infected patients in Africa as non-nucleoside reverse transcriptase inhibitors (NNRTI) based ART is first line and the alternative NNRTI nevirapine is associated with more toxicity and drug interactions.

It is unclear at which point in treatment, exposure to anti-TB drugs is most critical to prevent evolution of drug resistance MTB populations. On one hand, early on in treatment, the replicating bacterial load is greatest and the host is likely to be most immunocompromised. On the other hand, later on in treatment, around 2months, patients are being switched from quadruple therapy to dual therapy. It maybe at this point, that individuals with significantly lower concentrations of anti-TB drugs, could be at greatest risk of selecting for drug resistant populations. This is particularly pertinent in those with a greater bacterial burden e.g. cavitatory disease, who are frequently still culture positive in the sputum at this time.(4) At 2 months, there is also assurance that rifampicin autoinduction is complete and rifampicin steady state has been reached.

From a logistical point of view, in a programmatic setting, patients are reviewed with attainment of follow up sputa at 2 months post commencement of treatment. It is at this point, that patients are monitored for evidence of sputum smear/culture conversion and if non-converters, DST will be performed to check for drug resistant MTB. It would hence, be relevant to accumulate concurrent PK data at this time point so that conclusions

regarding the role of PK variability in the acquisition of drug resistance which are reached from the study, can be applicable to a programmatic setting.

Because we aim to determine the frequency and risk factors for ADR within a programmatic setting it is essential that the DST that is performed on patients in this study conforms to that advised in the TB programme guidelines. The TB treatment guidelines in the Western Cape Province have been recently updated with specific recommendations regarding DST at different timepoints during treatment. We have thus altered our DST schedule to be done in real-time to be in line with these. We will ensure that all patients on the study receive these tests either within the clinical service or performed by study staff:

- GeneXpert at baseline (it is an inclusion criteria that all patients are GeneXpert positive for MTB at baseline)
- Patients with baseline rifampicin resistance as diagnosed on GeneXpert will have secondline DST for ofloxacin, amikacin and ethionamide
- If smear positive at baseline and remains positive at 2 months then line probe assay *(MTBDRplus)* directly on specimen, followed by culture and second line drug sensitivities if MDR TB

The only additional DST test that we will perform (compared with the programme guidelines) in real time is at 2 month follow up: all patients with baseline rifampicin susceptiibility will have GeneXpert testing to screen for acquired rifampicin resistance.

We will however also perform cultures on all patients at 0, 2 and 5 months and store the isolates from positive cultures for potential future drug susceptibility testing (phenotypic and/or line probe assay) to detect amplified drug resistance. This storage (and not performing this additional DST in real time) is being done for both cost reasons as well as to ensure that our findings reflect the programmatic setting.

Measure of adherence in TB treatment is challenging. The data suggests that directly observed therapy, which would hypothetically maximise adherence, does not appear to improve clinical outcomes.(121) It is not feasible to admit patients as inpatients for the duration of the study. Hence, to measure adherence, we will review TB treatment charts, keep track of pill counts from returned blister packs and measure a urinary metabolite of isoniazid on 2 occasions. The latter is only a measure of recent adherence.

It is possible that disease resolution due to TB treatment alone will also cause change in TB PK over time, some of which can be explained by measurement of known covariates such as weight gain. Our study would not aim to quantify the effect of disease resolution

secondary to TB treatment on TB PK. We assume that this effect will occur in all TB patients, both mono-infected and HIV/TB co-infected.

We choose to focus on rifampicin, isoniazid and pyrazinamide as there is most data showing that inadequate concentrations could be linked with poor clinical outcomes. (55)(56)(24) They have also shown more PK variation over time compared with ethambutol.(69) We will not do PK analyses of second line drugs in patients with baseline MDR TB as we would not be sufficiently powered to assess differences in PK between cases of acquired drug resistance and controls.

MTB is believed to have an array of phenotypes in patients with active disease. Depending on the surrounding microenvironment and metabolic state, the bacilli can range from actively replicating to persistence and stationary (103,122). In vitro culture models have shown that MTB at different growth phase (exponential, stationary or starvation) have differential mRNA and small RNA (sRNA) expression ratio to ribosome RNA (rRNA). A panel of RNA targets representative of these different growth phases has been selected. Comparative expression analysis of RNA extracted from MTB in sputum to the in vitro benchmark will offer insight on the phenotypes of the bacilli in patients. Expectorated sputum is believed to originate from active infiltrates or liquefied lesions, recapitulates the bacterial population in the lungs and may harbour MTB with a variety of phenotypes. To better understand the phenotype of MTB and the transcriptomic changes induced by antibiotic treatment, we plan to study MTB in the sputum collected from patients displaying active TB disease, with or without concurrent HIV co-infection, during the course of TB treatment. . We aim to perform RNA-seq on bacterial RNA extracted from sputum, collected prior to and along the course of treatment. In our experience, approximately 25% of the patients will display “persistent” bacteria, which remained detectable after 8-weeks of antibiotics. Understanding whether specific genes alter their expression profiles to increase the bacteria’s survival in response to antibiotics will be a key to study the emergence of persistent and drug-resistant bacteria as well as identify gene products that should be targeted for pathogen clearance.

Hypotheses

1. Inadequate drug exposure and HIV co-infection predispose to ADR in persons being treated for *M. tuberculosis*.
2. The change in exposure to rifampicin, isoniazid and pyrazinamide secondary to commencement of ART is not significant having adjusted for appropriate quantifiable covariates.
3. Certain transcriptional changes of the MTB genome allows the mycobacteria to persist during antibiotic treatment
4. Co-infection with HIV decreases immune selection pressure on TB and the transcriptome of MTB isolated from co-infected patients will differ from those without co-infection.

   Collaborative TB genomics project

5. The overall hypothesis is that the molecular mechanisms underlying the development of drug resistance in MTBC differ in HIV-infected TB patients when compared to HIV-negative TB patients, and that this difference results from the inherent differences in immune competency. In particular, we will address the following three hypotheses:
   a) Drug-resistance-associated mutations in Mtb (including compensatory mutations) may differ in HIV-positive patients, compared to HIV-negative patients, and this as a result of the degree of immunodeficiency.
   b) The level of phenotypic drug resistance resulting from particular drug-resistance-associated mutations may differ in isolates collected from HIV-positive patients, compared to HIV-negative patients.
   c) The levels of phenotypic drug resistance are associated with both particular drug-resistance-associated mutations and particular MTB lineages.

Research questions

1. What is the frequency of ADR in patients undergoing therapy for pulmonary TB in a programmatic setting in Western Cape, South Africa?
2. Are HIV co-infection and the degree of immunodeficiency secondary to HIV risk factors for ADR?
3. Do rifampicin, isoniazid and pyrazinamide exposure (as measured by C_{max}/AUC) change inimmunocompromised HIV-infected patients (CD4 count ≤100cells/mm^3)

post commencing ART comprising of 2 nucleos(t)ide reverse transcriptase inhibitors and efavirenz and if so, is this independently due to ART?

4. Are C_{max}/AUC of rifampicin, isoniazid and pyrazinamide lower in cases of ADR compared with controls?
5. What are the mechanisms for acquisition of drug resistance?
6. What is the phenotype of MTB in sputum?
7. What is the transcriptomes of MTB in sputum prior to and during drug therapy?
8. Are there specific genes that allow MTB to persist during drug therapy?
9. Does HIV co-infection alter the phenotypes and transcriptomes of MTB?

Aims

1. Mechanistic separation ofthe independent effect of ART on exposure ($C_{max}$and AUC) to rifampicin, isoniazid and pyrazinamide in HIV co-infected persons with CD4 count ≤100 cells/mm^3undergoing therapy for pulmonary TB
2. To identify individuals who develop ADR during TB treatment and examine risk factors for ADR.
3. To compare PK profiles of cases of ADR with matched controls
4. To build up a clinically well-defined collection of serially obtained MTB isolates collected during follow up in which full genome analysis can be used to study microevolution of drug resistance
5. To characterise the transcriptomes of the mycobacteria (mRNA and sRNA) during chemotherapy and to determine if expression of specific genes is related to persistence.
6. To investigate the phenotype of MTB pathogen in the sputum
7. To examine the impact of HIV co-infection on the phenotype and transcriptome of MTB

Methodology

Patients consenting to the study will continue to receive on-going investigation and treatment by the clinical medical staff at the presenting facility, according to national guidelines for the treatment of tuberculosis and HIV. Patients will be recruited for the study and seen at follow up visits by the lead investigator, with assistance of designated research nurses.

Study population, clinical site and treatment regimens

The patients will be enrolled to the study at presentation to clinical services in Cape Town. The majority of recruitment (80%) will occur at Ubuntu clinic, Site B, Khayelitsha. Professor Wilkinson's group is currently enrolling patients for other HIV and TB studies at Ubuntu clinic. A minority (20%) of patients will be co-recruited at Khayelitsha District Hospital where Prof Meintjes is recruiting patients to an ongoing concurrent study. Follow up will occur at Ubuntu clinic. Patients will also be seen for their follow up appointment at Khayelitsha District Hospital, G. F. Jooste, D.P. Marais and Brooklyn Chest Hospital if they are admitted as inpatients during the study. Eligible patients will be identified by a designated Xhosa and English-speaking research nurse, they will be informed about the study using a standard information sheet (which will be available in Xhosa and English) and invited to participate in the study. Only patients who provide written informed consent will be enrolled. We will sequentially recruit patients who present to the TB clinic on meeting appropriate criteria. This will minimize selection bias.
On study completion, we will undertake a database query of the electronic TB register at Ubuntu clinic to ascertain that the study cohort is generalizable to the overall clinic in terms of clinical demographics (gender, % retreatment, % HIV co-infection, median CD4, % on ART at diagnosis) and treatment outcomes (death and failure) in microbiologically confirmed cases of TB. This will be done in liaison with Prof Andrew Boulle at the Western Cape Provinicial Department of Health. This database will have user-level security and will be password protected and will be accessed only by the principle user Dr. Rockwood.

PK studies will take place at Khayelitsha District Hospital. On meeting eligibility criteria, potential participants will be separately consented for the PK studies.

Patients will be allocated to specific groups as per the following criteria:
Group A: HIV positive TB patients who are ART naive or have not taken ART in the past 2months and who have CD4 lymphocyte count ≤100 cells/mm^3
Group B: HIV positive TB patients who do not fall into group A
Group C: HIV negative TB patients
Group D: HIV positive patients who have respiratory symptoms (TB suspects) but are culture negative for TB
Group E: HIV negative patients who have respiratory symptoms (TB suspects) but are culture negative for TB

The decision regarding ART timing will be taken by the HIV-TB clinic based on CD4 count criteria and provincial guidelines. Unless contra-indicated, patients in group A will commence ART by 2 weeks of commencing TB treatment.

Inclusion criteria:

- Patients will be enrolled if diagnosed with MTB on testing of sputum using GeneXpert

Exclusion criteria

- Age <18 years
- Pregnancy
- Unknown HIV status
- Unable to give informed consent (cognitive deficit), language barrier
- Unable to provide baseline sputum sample either spontaneously or via induced sputum
- Mental illness
- Prisoners
- Have had >3 doses TB therapy
- Been on TB treatment within the last month (excluding current episode)
- Any factor which precludes commencement of standard recommended regimen (as per national protocol) for drug sensitive or drug resistant TB

Further exclusion criteria for Group A only (if any of these apply, patient will be re-allocated to Group B)

- Excessive alcohol intake (>6 u/day) or current recreational drug use
- Weight <30 kg
- On concurrent enzyme inducing drugs
- If patients are unwilling to commence ART by 4 weeks post commencement of TB therapy

Patients will be identified when commencing therapy for pulmonary TB at Ubuntu Clinic, Site B Khayelitsha or as inpatients at Khayelitsha District Hospital. Participants may be enrolled if they have a positive GeneXpert test for MTB. Both rifampicin sensitive and rifampicin resistant cases are eligible for enrolment. Diagnosis will be confirmed by sputum culture and drug sensitivity testing as described in microbiological analysis section below.

Drug sensitive TB treatment will be delivered in a 4-drug fixed dose combination supplied by the National Tuberculosis Control Programme (Rifafour e-275 [Sanofi-Aventis, South Africa]. Each tablet will contain rifampicin at 150 mg, isoniazid at 75 mg, pyrazinamide at 400 mg, and ethambutol at 275 mg. Weight band-based dosing will be used in line with

WHO guidelines. Patients weighing 30 to 37 kg, 38 to 54 kg, 55 to 70 kg, and >70 kg will be given doses of 2, 3, 4, and 5 tablets, respectively 7 days a week for the first 2 month intensive phase. They will be switched to rifampicin/isoniazid during the continuation phase for a further 4 months. This is regimen 1. The TB regimen for retreatment cases is (Regimen 2): rifampicin/isoniazid/ethambutol/pyrazinamide plus streptomycin for 2 months,rifampicin/isoniazid/ethambutol/pyrazinamide for 1 month and then rifampicin/isoniazid/ethambutol for 5 months. In patients starting tenofovir during the intensive phase of this regimen the streptomycin is stopped when the tenofovir is started because both drugs are potentially nephrotoxic. It is likely that this Regimen 2 will be altered by the Department of Health in the near future, with streptomycin being omitted.

Multidrug resistant TB treatment will be delivered by the TB service as per national protocol with a 6-month intensive phase of 5 drugs (Ethionamide/Pyrazinimide/Kanamycin/Moxifloxacin/Terizidone) followed by an 18-month continuation phase with 4 drugs (Ethionamide/Pyrazinimide/Moxifloxacin/Terizidone).

On referral, patients will have a consultation with a member of the study team who will conduct the informed consent process using “Consent form for Patients” available in Xhosa and English (languages commonly spoken by patients attending study sites). See “Informed consent process” below.

All patients presenting with a diagnosis of TB are offered HIV counselling and testing (HCT) within the clinical service and uptake is >95% in Khayelitsha, outside the study setting. Patients who decline VCT will not be eligible for the study.

ART will be commenced at between 2 and 8 weeks in HIV-infected patients by the attending physician at Ubuntu Clinic as per national protocol. Individuals in group Awill commence ART as a matter of priority, most commonly 2 weeks post commencement of TB treatment. First line ART treatment will be with a combination of tenofovir/lamivudine/efavirenz. Other possibilities if tenofovir is to be avoided include zidovudine/lamivudine/efavirenz and stavudine/lamivudine/efavirenz.

Procedures

Screening visit- only for patients who are being started on TB treatment without microbiologically confirmed diagnosis of MTB

The aim of this screening visit is to explain the purpose of the study, obtain informed consent and obtain 2-3 induced sputum samples, one of which will be sent for GeneXpert and culture to confirm presence of MTB. Other sputa will be stored in case of culture contamination and for future research, should the patient be enrolled into the study. At screening visit, the patient will also be screened for pregnancy (if female of child bearing age) and will be referred for voluntary counselling and testing for HIV, if they have not

already been tested through the integrated HIV/TB clinic. No clinical record form will be filled at screening visit. Patients will be given a follow up date for their sputum results in 2-3 days where if found to have a microbiologically confirmed diagnosis of TB, they will be consented to participate in the study. Their participation will not affect TB treatment which will be continued by the clinic. If these screened patients are found to be eligible and are successfully enrolled into the study, at baseline visit ABC1, they will not be required to provide further induced sputa as the samples would have already been obtained at the screening visit.

Study visit ABC1 -Group A, B and C

Patients must be seen within 3 days of commencing TB treatment.

During a standardised clinical interview and examination, anonymised data will be collected by the study investigator on all patients using study data collection forms and will be entered into an anonymised database:
- Demographic information (age, sex, ethnic origin, occupation)
- Focused medical history including symptoms, past medical history including previous TB history (previous treatment for LTBI and active TB, drug resistant TB contacts, failed and default TB treatment). Details of current TB regimen and concurrent medication will be recorded. Patients will be screened for misuse of alcohol .Smoking status will be classified as ever or never smoked.
- HIV-related opportunistic infections and appropriate WHO staging.
- Clinical examination findings (general including body mass index, respiratory, cardiovascular, abdominal, neurological)
- Patients will be screened for pregnancy (if women of child bearing potential) with testing of urinary BHCG.

The hospital medical notes and investigation results will be reviewed by a member of the study team and information recorded will be:
- Chest radiographic appearance if chest radiograph has been performed as part of routine care (see additional notes below).
- GeneXpert, sputum microscopy and culture results (including time to detection)
- Recent CD4 count & viral load (if HIV positive)
- Relevant blood tests (e.g. full blood count, ALT, albumin, creatinine)

A venous blood sample (30ml)will be collected by a trained experienced phlebotomist (study team member), using universal precautions to minimise risk of needle stick injury. This will be used for:
- If the patient is HIV-infected and does not have a recent CD4 count recorded (within 1 month prior to recruitment), a CD4 will be requested. A HIV viral load (VL) will be performed on all HIV positive patients at the time of recruitment. A sample will be sent for HIV serology in those who test HIV negative on point of care testing.
- Biobank- for host pharmacogenomic and cytokine profiling and saved serum

Three induced sputa will be collected by following the induced sputum protocol (see below). One sputum sample will be used for:
- Microbiological analysis- smear grading of AFB (Ziehl-Neelsen staining),mycobacterial culture and evaluation of time to detection(BACTEC MGIT 960 system, Becton Dickinson, Sparts, Maryland). Drug sensitivity testing via BACTECT 960 system and rapid line probe assay (MTBDR*plus* test, HainLifescience, Nehren, Germany) will be done at a later date on stored culture isolates. All strains which are rifampicin and isoniazid resistant will undergo culture based drug sensitivity testing for ethambutol, pyrazinamide, streptomycin, ofloxacin, ethionamide and the second-line injectable agents (amikacin, kanamycin and capreomycin)
- Growth on LJ medium for future DNA extraction
- The other 2 sputa will be frozen and retained along with MGIT cultures

All patients will have a chest radiograph as part of the study, if not done routinely by their treating clinician. The chest radiograph will be reviewed by the study clinician (Dr Rockwood) who will receive training in Chest Radiograph Reading and Recording System and evaluation made of a) the number of lung fields in which opacities are presentb)presence of lung cavities c) number of zones of lung affected by cavities. Patients with opacification in more than the upper lobe or involvement of both lungs will be classed as having extensive disease.

Significant clinical results will be communicated by the study clinician to the treating clinician.

<u>Collection of induced sputum</u>

Any patient with significant respiratory distress (respiratory rate > 30) or bronchospasm will not have induction done.

Collection - Sputum induction will be performed in accordance with a locally agreed standard operating procedure which is already in use for routine clinical care. All induction will be performed in a designated collection room, isolated from all other clinical buildings. Following each induction, the chamber will be fully aired to prevent cross-contamination.

All sputum induction will be supervised by a member of the study team to ensure participant safety, consistency and adequacy of sample collection. Sputum will be induced with 10 – 40 ml of 3 - 5% saline delivered via an ultrasonic nebuliser. Single-use face masks, filters and tubing will be used. Subjects will be asked to clear their mouth and nose of any residual saliva or secretions using a paper towel. Sputum will be induced in five minute blocks to a total of 20 minutes. The reporting of chest discomfort will trigger discontinuation of the procedure. Subjects will be asked to repeat the process of nose and

mouth cleaning prior to any attempt to expectorate sputum. Sputum may be expectorated at any point during the 5 minute cycles if the subject feels a sample is ready. Sputum will be expectorated without assistance into 3 sterile containers. One induced sputum procedure is expected to generate 3 induced sputa samples.

The patient will be unaccompanied in the chamber during the 5 minute periods of nebulisation, but kept under observation. At the end of nebulisation, the subject will remain in the chamber until coughing has ceased, after which they will be free to leave and the chamber will be left to air prior. Researchers will wear N95 masks whilst assisting the patient with the sputum induction procedure.

Induced sputa samples will be labelled using the study identifier only and sent to the TB reference laboratory. Results will be communicated to treating clinicians when they become available.

Sputum processing

All manipulations with potentially infectious clinical specimens are performed in a Class II safety cabinet in a BSL2 laboratory. Sputum specimens are decontaminated with N-acetyl-L-cysteine-sodium hydroxide. After centrifugation, the pellet is suspended in 1.0 ml of phosphate buffer (pH 6.8). A concentrated auramine smear is prepared and examined under 3500 magnification using a fluorescent microscope and graded according to International Union Against Tuberculosis and Lung Disease (IUATLD) guidelines. A 0.5-ml portion
of the sediment will be cultured using the BACTEC MGIT 960 system. BACTEC vials are incubated at 37°C and the growth index (culture status) read daily at an identical time to limit reading variability. The time to detection (TTD) is recorded as the number of days required for each vial to reach a growth index of 10. Cultures are monitored for 42 days before being classified as negative.

The MTBDR*plus* is performed according to the manufacturer's instructions. A 500 µl portion of the decontaminated sediment is used for DNA extraction, a 1-hour process that includes heating, sonification, and centrifugation. The amplification procedure consists of preparation of the master mix and addition of the DNA also required 1 hour. Hybridization is performed with the GT Blot 48 (HainLifescience), which is an automated hybridization machine. After hybridization and washing, strips are removed, allowed to air dry, and fixed on paper.

Study visit A1 -Group A only

Intensive PK pre ART (day 11-17 post commencement of TB treatment)

The purpose of study visit A1 is to establish rifampicin, isoniazid and pyrazinamide PK profile prior to commencement of ART. ART will commence the day after study visit 2. The protocol for intensive PK evaluation is described below.

Women of child bearing age will be screened for pregnancy via urinary βHG testing

Patients will be weighed, a record made of adherence via use of TB treatment cards and a record made of any concomitant medications.

Study visit ABC2- Group A, B and C

Study visit ABC2 is at day 57-63 post commencement of TB treatment

Patient treatment, adherence and clinical status will be reviewed. Adherence will be assessed via review of TB treatment cards, returned blister packs and a urine sample will be taken for testing of the metabolite acetylisoniazid (acetylINH). Patients will be weighed. Three induced sputum samples will be taken. One sputum sample will be used for:

- Genotype MTBDRplus (HainLifescience, Nehren, Germany) which will be done directly on the specimen
- Mycobacterial culture and growth on LJ agar solid medium for future DNA extraction
- Drug sensitivity testing via BACTEC 960 system and rapid line probe assay (MTBDRplus test, HainLifescience, Nehren, Germany) will be done at a later date on stored culture isolates.All strains which are rifampicin and isoniazid resistant will undergo culture based drug sensitivity testing for ethambutol, pyrazinamide, streptomycin, ofloxacin, ethionamide and the second-line injectable agents (amikacin, kanamycin and capreomycin)

-The other sputum samples will be frozen and retained along with MGIT cultures

A 10 ml venous blood sample will be stored as biobank for future studies

* Study visit ABC2 will be brought forward if any patient clinically deteriorates as assessed by study physician (Dr. Neesha Rockwood).Patients will be given contact details for the study team and information on how to recognise severe symptoms e.g. recurrence of fever for >1day, worsening cough or difficulty in breathing and access emergency care, should the need occur. The patient will be informed that the study team is unable to provide emergency care, but should the patient clinically deteriorate, the patient will be asked to contact the clinical team for review at the earliest possible time.The study team will facilitate emergency care at Ubuntu and District Hospital by consulting with doctors in the clinical service and referring patient appropriately.

Study visit A2- Group A

PK evaluation post ART (day 57-63 post commencement of TB treatment). For Group A, Study visit A2 and visitABC2 will be combined on a single day.

The purpose of study visit A2 is to establish rifampicin, isoniazid and pyrazinamide PK profile at 2 month follow up.The protocol for intensive PK evaluation is described below.

Patients will be weighed, and a record made of any concomitant medications. A venous blood sample will be taken to check serum albumin. A serum sample will also be stored in order to measure an archived HIV viral load (as a surrogate of efficacy of ART) if indicated at a later date.

Women of child bearing age will be screened for pregnancy via urinary βHG testing

Study visit BC1- group B and C

ADR PK evaluation (within 5 days of GeneXpert result from Study visit ABC2)

Study visit BC1 will only be for patients who develop rifampicin resistance and matched controls.

Development of rifampicin resistance will be used as a surrogate measure of ADR.

Cases of rifampicin resistance - These are patients whose sputa are positive for MTB and have mutations in rpoB gene which are picked up on GeneXpert testing during visit ABC2.

Controls- These are individuals whose sputa are negative for MTB or positive for MTB with no rifampicin resistance picked up on GeneXpert testing during visit ABC2. They will be matched for HIV serostatus and CD4 count strata , weight and sex.

Patients will be weighed, and a record made of any concomitant medications. A venous blood sample will be taken to check serum albumin.

The protocol for intensive PK evaluation is described below.

Study visit ABC3- group A, B and C

Study visit ABC3 is at 147-153 days post treatment. This visit will only be for patients who are culture positive during visit ABC2

Patient treatment, adherence and clinical status will be reviewed. Adherence will be assessed via use of TB treatment cards and returned blister packs. Patients will be weighed. Three induced sputum samples will be taken. One sputum sample will be used for:

- Genotype MTBDRplus (HainLifescience, Nehren, Germany) which will be done directly on the specimen
- Mycobacterial culture and growth on LJ solid medium for future DNA extraction
- Drug sensitivity testing via BACTEC 960 system and rapid line probe assay (MTBDRplus test, HainLifescience, Nehren, Germany) will be done at a later date

on stored culture isolates. All strains which are rifampicin and isoniazid resistant will undergo culture based drug sensitivity testing for ethambutol, pyrazinamide,streptomycin, ofloxacin, ethionamide and the second-line injectable agents (amikacin, kanamycin and capreomycin

The other sputum samples will be frozen and retained along with MGIT cultures

A 10ml venous blood sample will be collected as biobank for future analyses.

Study visit BC2- group B and C

ADR PK evaluation (within 5 days of GeneXpert result from study visit ABC3)

Visit BC2 will only be for patients who develop rifampicin resistance (picked up during visit ABC3) and controls who have not already previously participated in a PK study.

Cases of rifampicin resistance - These are patients whose sputa are positive for MTB and have mutations in rpoB gene which are picked up on GeneXpert testing during study visit 7.

Controls- These are individuals whose sputa are negative for MTB on GeneXpert testing.They will be matched for HIV serostatus and CD4 count strata , weight and sex.

Patients will be weighed, and a record made of any concomitant medications. A venous blood sample will be taken to check serum albumin.

In addition to ABC1 (baseline visit), ABC2 (2 month study visit) and ABC3 (5 month study visit), patients may be recalled to expectorate at other time points for the following indications:

- If multidrug resistance is suspected based on the most recent culture and drug sensitivity result and this needs to be confirmed via sending off of another sample. The rationale for this is to rule out laboratory contamination, potential mix up of samples or false positive results. A specimen will be concurrently sent on the patient’s clinic number with specific arrangements for extended drug sensitivity testing if appropriate to guide clinical management. All results are fed back to the attending clinician and acted upon in real time.
- If a culture is contaminated or an environmental non-tuberculous mycobacteria is suspected
- If a patient was unable to attend at the 5 month time point e.g. travelling outside Western Cape and is then able to attend at a later time point. The purpose of having a repeat sputum at or post end of antitubercular therapy is to confirm microbiological cure as per WHO guidelines.

Proposed collaborative multi-regional TB genomic study

Overall, half of the MTB isolates will be multidrug resistant and half pansusceptible. These 1,000 MTB isolates will undergo whole genome sequencing at the Broad Institute in Cambridge, USA. Quantitative phenotypic drug susceptibly testing will be done for 12 anti-TB drugs at the National Center for Mycobacteria in Zurich. Genome sequences will be analysed to identify different drug-resistance-associated mutations (including compensatory mutations) and MTB lineages. These will then be correlated to quantitative levels of drug resistance. The impact of HIV infection and MTB lineages on drug resistance at the molecular and phenotypic level will be elucidated. As South African collaborators, we would send 120 drug sensitive MTB strains for whole genome sequencing and phenotypic drug sensitivity testing. These strains would be grown from sputum samples which were collected as part of the ongoing ADR Study. Corresponding sociodemographic data, clinical signs and symptoms, radiological data, mycobacteriology data including drug sensitivity testing, current and previous antituberculosis therapy and treatment outcomes for these 120 TB patients would be entered electronically into a secure Research Electronic Data Capture (RedCap) database centrally maintained by Clinical Trials Unit, University of Bern.

sRNA sub-study

Participants will be eligible for the sRNA sub-study if they have smear positive pulmonary TB and have taken 1 dose or less of TB treatment. Visits for the sRNA sub-study are at:

1. Baseline – prior or within 24 hours of commencing TB therapy
2. Week 1-2 – between 7-14 days of commencing therapy
3. Week 4 – between 25-28 days of commencing therapy
4. Week 8 – between 56-63 days of commencing therapy

At each study visit we will take 2 x 3ml of blood for tempus. This will be utilised for host microarray study and will allow us to investigate host transcriptional changes, particularly comparing patients who are responding to treatment and those who have persister bacteria. We will also take 2x 4ml of blood for EDTA to study the level of cytokines and chemokines in plasma, indicating host immune response at the protein level.

<u>Sample collection, processing and analysis</u>

Patients participating in sRNA sub-study will be asked to expectorate 2 sputum samples and sputum induction with hypertonic saline will be done only if patients are unable to spontaneously expectorate. Sputum will be collected into 40 ml tubes. Sputum handling and manipulation will be performed in open air or well-ventilated room whilst wearing protective gear including protective eyewear, N95 face mask, apron and gloves. TRIzol will be added to one sample. The volume of

sputum in collection tube will be estimated. TRIzol reagent will be added at approximately 3 times the volume of sputum to original sputum sample with a pasteur pipette. Samples will be manually vigorously shaken for 1 minute with lid firmly screwed on. The specimen will be transported on ice to the IIDMM on the same day as collection and stored at -80 C for future batched analysis. RNA will be extracted from smear-positive samples with the RNAqueous-Micro kit (Ambion) according to manufacturer's instructions. We then aim to quantify the level of expression of sRNAs by qRT-PCR. Gene expression levels will be normalized to that of 16S rRNA to compensate for intra- and inter-kinetic RT-PCR variations. It will be the ultimate aim to perform RNA-seq (Whole Transcriptome Shotgun Sequencing) on these samples. Sample analysis will be performed at the Division of Mycobacterial of the National Institute for Medical Research (NIMR) in London. The other sputum sample will be sent for liquid mycobacterial culture.

Protocol for Intensive PK evaluation

Intensive PK evaluation	Group	Time post commencement of TB treatment	Days /weeks post commencement of ART	Other information
Pre ART	A	11-17 days	pre-ART	
Post ART	A	57-63 days	Between 4-6 weeks	
ADR/controls	B/C	approximately 2 months		Within 5 days of GeneXpert result from Study visit ABC2)
ADR/controls	B/C	approximately 5 months		Within 5 days of GeneXpert result from study visit ABC3)

Patients will undergo peripheral venous cannulation in order to avoid multiple venopuncture and thereby minimise patient discomfort. If patients prefer to avoid cannulation, the option of multiple venopuncture will be given.

TB drug doses will be taken after an overnight fast of at least 8 hours and observed by a study team member. Blood specimens of at least 2ml will be collected in heparinized tubes. Timings of collection include just prior to the TB drug doses and at 1, 2, 3, 4, 6 and 8 hours after dosing. Patients will eat a standardized breakfast 2 hour post TB drug dose and lunch 5 hours post TB drug dose. The sample will be immediately placed on ice and plasma separated by centrifugation (1500 x g for 10 minutes) within 30 min before storage at -80°C until analysis. The storage tubes containing the plasma samples will be

transferred to the analytical laboratory (Division of Clinical Pharmacology, University of Cape Town) in liquid nitrogen.

Plasma drug concentrations will be quantified by tandem high performance liquid chromatography (HPLC) mass spectrometry.Tandem HPLC mass spectrometry (Applied Biosystems API 2000) will be used. Plasma protein is precipitated with acetonitrile. Samples are vortexed and centrifuged for 5 min at 750 g. Supernatant (5 mL) is injected into the column. Standard curves are plotted. Quality control samples covering the ranges are included with each run. Inter- and intra-day coefficients of variation will be noted.

Statistical Analyses

Data will be analysed using a range of statistical tests. A noncompartmental analysis (NCA) approach will be used to calculate the PK measures and to compare AUC and C_{max}between cases of ADR and matched controls. Multilevel linear mixed-effects regression will used to examine the effects of ART and covariates on anti-TB drugs C_{max}/AUC over time.

Sample size calculation

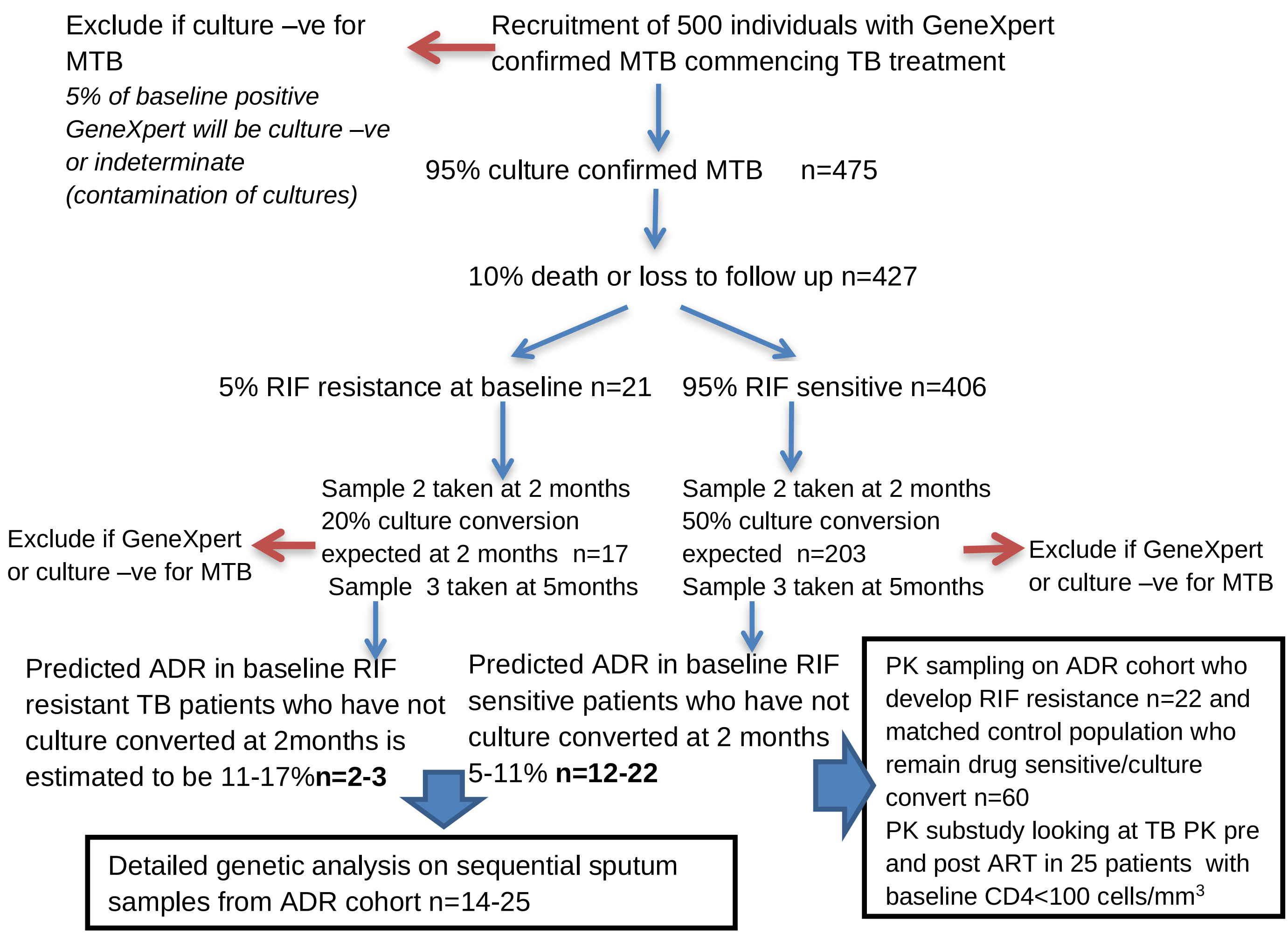

Power calculation for hypothesis 1

Inadequate drug exposure and HIV co-infection predispose to ADR in persons being treated for M. tuberculosis

We assume cases of ADR n=22
Weight, sex and HIV matched controls n=60
Minimal detectable difference= 30%
SD of cases= SD of controls
Coefficient of variation = 50%
alpha = 0.05 (2 sided)
Beta= 0.75
(SD= standard deviation)

Power calculation for hypothesis 2

The change in exposure to rifampicin, isoniazid and pyrazinamide secondary to commencement of ART is not significant having adjusted for appropriate quantifiable covariates

We will set difference in C_{max}pre and post ART <0.5as an equivalence threshold

H0: The difference (d0) between c_{max} (or AUC) pre and post ART is >=0.5

HA: The difference (d0) between c_{max} (or AUC) pre before and post ART <0.5. Hence, rejection of the H0 would further imply (since d0 is so small as to be clinically unimportant) that c_{max}pre and post ART after are the same.

Cases of recruited patients with CD4 ≤100, n=25
d0= 0.5
mean C_{max}= 7.8
SD= 2
alpha= 0.05 (1 sided)
Beta = 0.8
(paired sample calculation)

Sample size considerations for sRNA sub-study

We aim to recruit 20 patients each in Group A and B, 32 patients in Group C and 4 patients each in Group D and E. In our experience, approximately 25% of the patients undergoing chemotherapy will have culture positive MTB after 8 weeks of treatment. Based on this presumptive knowledge, there should be 10 HIV positive TB patients (5 with CD4 count <100 cells/mm^3 and 5 otherwise) and 8 HIV negative TB patients who remained to have detectable MTB at 8 weeks post-treatment. This sample number will give us enough power for comparative analysis. Finally, we aim to recruit total of 8 control patients who present with respiratory symptoms but are cultured negative for MTB (4 with HIV and 4 without). Samples from these patients should not contain any MTB and will be used as background control. The control patients will only be seen at baseline.

Confidentiality

All information collected and documented for the purpose of the study will remain confidential. Participants will be assigned a study number which will label all written/electronic data held for the purposes of the study and on samples. The study number will be the only patient identifier used on the data collection forms and the

electronic database. Data linking the study code to the patients' identity will be password protected on a laptop, with a single written copy kept locked in a secure location at the study site, in case subsequent information gained by study is directly relevant to patient care and the patient needs to be traced.

All clinical data entered onto the secure Research Electronic Data Capture database centrally maintained by Clinical Trials Unit, University of Bern would be fully anonymised and the only patient identifier would be their ADR study number and laboratory code for their MTB isolate.

Social/Economic & Ethical implications

The social, economic & ethical implications for patients donating venous blood and induced sputum for the purpose of these experiments are not predicted to reveal any information that could have further ethical or economic implications for individual patients.

The sputa stored from the screening visit will be disposed of in a manner compatible with appropriate biological safety containment if found to be negative for MTB or if the patient subsequently declines participation in the study. Specific consent will be sought to biobank blood samples for future testing, including host pharmacogenomic testing as described under procedures. We will not be testing specifically for mutations associated with inherited genetic diseases. Any future research using these samples will be subject to ethical review and all samples will be anonymised prior to genetic testing. It will be impossible to trace these results back to individual patients. Therefore, the results of these tests will have no additional impact on individual participants.

The procedures of, peripheral venous cannulation, venepuncture and sputum induction are minimally invasive, generally well tolerated and have no known serious side-effects.

Potential adverse effects for participants

1) Bronchoconstriction may occur during sputum induction but is readily treated with bronchodilators. There are no reports of serious cases in the literature.

2) Nausea and vomiting occurs very rarely and is mild and transient.

Peripheral venous cannulation and venous blood sampling:

1) Bruising may occur commonly but is mild and well tolerated.

2) Pain transiently occurs but is mild.

3) Thrombophlebitis is a rare complication from a peripheral venous cannulation. Aseptic technique will be used to minimise infection risks and apart from saline flushes, there will be no infusions.

No haemodynamic consequences are predicted from removing the volume of blood required for the study.

Chest radiography:

1) Radiation exposure has been associated with an increased risk of malignancy, however the radiation dose required for a single chest radiograph is extremely small and highly unlikely to contribute to a significantly increased risk of malignancy. Most patients recruited will require a chest radiograph as part of routine clinical care.

2) Chest radiography is a simple, quick and well-tolerated procedure used frequently in clinical practice and for screening purposes for a number of pulmonary conditions.

Participants will be informed of these side effects when giving informed consent.

Financial implications

Subjects will compensated per study visit for their inconvenience, discomfort and travel expenses when contributing to the study. This will be R30 per screening visit, ABC1, ABC2, ABC3, sRNA sub-study and unscheduled visits and R200 per visits A1,A2,BC1 and BC2 (when PK sampling is done requiring patients to spend the day at the clinic). There will be no additional financial cost for participants in addition to that incurred by their standard hospital care.

Time expected for participation

It is estimated that participation in the study will require:

- 30 minutes per screening visit
- 1-2 hours per visits ABC1, ABC2 and ABC3
- 8-9 hours per visits A1, A2, BC1 and BC2.
- 30 minutes per additional sRNA sub-study visits at 1-2 and 4 weeks of TB treatment
- 30 minutes per unscheduled visits

It should be noted that patients in group A are likely to have an approximate 21 hours of study involvement and patients in group B and C, 4 to15 hours of study involvement. There will be adequate time allowed in all cases for patients to consider informed consent. Participation will not interfere with routine clinical care.

Benefits to participants/ patients

In general, the results of the study may influence future medical practice and indirectly benefit the patient population by improving clinical care.

This study uses MTBDRplus directly on sputum specimens to pick up isoniazid and rifampicin resistance at 2 and 5 months of follow up. This is not routine practice in the National TB programme where only a smear is performed. If positive, a second sample is sent at this point to check for culture conversion and if appropriate, phenotypic drug sensitivity testing. Hence, patients in the study may benefit from earlier detection of ADR and hence appropriate change of therapy at an earlier stage.

Performing sputum induction at follow up visits may have diagnostic benefit for the patient, as the yield of mycobacterial culture from induced sputum is increased compared to spontaneous sputum.

There are no direct benefits to patients from participating in the sRNA sub-study.

The PK analyses will be batched and run after the clinical study and thus will not be available in real time to affect patient management.

There are no direct benefits to patients whose MTB isolates will be sequenced for the proposed collaborative genomic study.

Potential adverse effects for researchers

1) Working with clinical blood samples: Researchers are experienced in handling blood samples and have been vaccinated against hepatitis B with detectable antibody levels. Routine precautions (wearing gloves, no use of sharps after phlebotomy) will be employed to minimise the risk of HIV transmission.

2) Sputum induction: Due to aerosolising of respiratory secretions there is a theoretical risk of increased transmission of TB, however this is minimised by physical isolation in a custom-made induced sputum facility during the sputum induction procedure and use of N95 masks by the patients and researchers at appropriate times.

3) TRIzol® Reagent contains toxic, corrosive and irritative compounds which could be a health hazard if not handled properly. Direct contact between TRIzol and skin, eyes, and lungs will be avoided by wearing protective wear at all times when handling TRIzol. Should

any part of the body come into direct contact with TRIzol, the exposed area will be washed vigorously with running water for several minutes and medical help sought.

4) All MTB isolates would be handled and prepared in the designated Biological Safety Level 3 laboratory.

Ethical statements

Regulatory compliance and conflicts of interest

The study complies with the Declaration of Helsinki 2008 (http://www.wma.net/e/policy/pdf/17cpdf) and the Department of Health ethical guidelines, 2004 (http://www.doh.gov.za/docs/factsheets/guidelines/ethnics).
No conflicts of interest are recognised by the study team. Research staff will not receive any incentives for recruiting participants or for any other purpose directly related to the study. Personnel involved in the design, conduct and analysis of the study have no proprietary interests relating to the study.

Informed consent process

The informed consent process will involve provision of a written and verbal explanation of the study using the information written on the consent forms, available in English and Xhosa. Patients will be given as much time as they require to consider the information and will be given the opportunity to ask further questions. A trained study team member will be responsible for ensuring that the patient is capable of giving informed consent i.e. that they have been given the appropriate information, have retained and can comprehend it. The study team member taking the consent will be required to countersign the consent forms to indicate this.

Patients will indicate consent by either a written signature or a thumb print. Patients will be given a copy of the consent form to keep in their chosen language with the study information and contact details for the study team. Patients may withdraw their participation in the study at any time.

Data entry and results

Data will be transcribed directly onto clinical record forms. All information and results gained through clinical assessment and investigation for purposes of the study that have implications for patient care will be passed on to the treating clinical team expediently. Laboratory results will be communicated to the clinical team within 5 days of availability to research team. Clinical and laboratory data will be recorded in a password protected Excel spreadsheet, with study number rather than name for patient identification.The PK analyses will be batched and run after the clinical study and thus will not be communicated in real time to the clinical team during the course of treatment.

Outcome

The results of the study will be published in international peer-reviewed journals of high impact, with an open access policy to ensure dissemination. The results will be written by Dr Rockwood for completion in her PhD thesis.

In addition, including as required for publication, data of particular interest from both studies will be released into the public domain with the assistance of both the Imperial College and University of Cape Town public relations departments. As the opportunity arises, data and lay interpretations of the findings will be presented to appropriate public fora and patient interest groups.The results will also be presented to doctors and nurses working in TB service in Khayelitsha.

Timetable and Milestones

	Sep '12-Dec '12	Jan '13-Apr '13	May '13-Aug '13	Sep '13-Dec '13	Jan '14-Apr '14	May'14-Aug '14	Sep '14-Dec '14	Jan '15-Apr '15
Site approvals/ethics								
Recruitment								
Follow up + intensive PK studies								
PK analyses								
Pathogen genome analyses								

Budget

Dr Rockwood has been awarded a Global Health Clinical Fellowship from the Wellcome Trust which provides partial consumables and Dr. Rockwood's salary. See Appendix 1 for budget details.

Key personnel

Robert John Wilkinson is a Wellcome Trust Senior Fellow in Clinical Science and Honorary Professor based in the Institute of Infectious Diseases and Molecular Medicine (IIDMM) at the UCT. Wilkinson's clinical specialty is Infectious Diseases with a special interest in TB. As part of specialist training, Wilkinson completed a PhD in Immunology from the Royal Postgraduate Medical School, Hammersmith Hospital, London and then undertook a 2 year Wellcome Trust Fellowship at Case Western Reserve University, Cleveland, OH working on aspects of genetic susceptibility to, and host bacillary interaction in, tuberculosis. Dr Wilkinson served on the governing body (Council) of the Royal Society of Tropical Medicine and Hygiene from 2001-2003, and was elected a Fellow of the Royal College of Physicians of London in 2003. He also serves on the executive committee of the IIDMM and directs its Clinical Infectious Diseases Research Initiative. Wilkinson has 19 years' experience in both clinical and laboratory aspects of TB.

Helen McIlleronis an Associate Professor in Clinical Pharmacology in the Department Clinical Pharmacology at UCT. Prof.McIlleronis the principal investigator of studies investigating antituberculosis and antiretroviral drug pharmacokinetics in patient populations. Drug-drug interactions in TB/HIV co-infected patients, optimizing dosing in neglected populations such as children and pregnant women and describing PK/PD relationships and interactions within antituberculosis regimens are key areas of interest.

Graeme Meintjes is an Associate Professor in Infectious Diseases in the Department of Medicine at UCT. Prof.Meintjes trained at UCT and is the principal investigator of studies into the pathogenesis and treatment of TB-IRIS. Professor Meintjes has 12 years clinical experience of HIV associated tuberculosis. He has been awarded an Intermediate Wellcome Trust Fellowship.

Sebastien Gagneux is an Associate Professor in microbiology in the Swiss Tropical & Public Health Institute, University of Basel, Switzerland. Prof.Gagneux is Unit Head of Medical parasitology and Infection Biology and Tuberculosis Research. His specialist interests include evolution & population genomics of *Mycobacterium tuberculosis*, evolution & ecology of antibiotic resistance in *Mycobacterium tuberculosis* andmolecular epidemiology of tuberculosis.

Neesha Rockwoodis the holder of a Wellcome Trust Global Health Clinical Fellowship. She is currently undertaking study for a PhD at Imperial College. Dr Rockwood holds a National Training Number (NTN) in HIV/Genitourinary Medicine in the London Deanery and is due to complete specialist training in 2017.

Douglas Young was appointed Fleming Professor of Medical Microbiology at Imperial College in 1993. Professor Young is also a Group Leader at the National Institute for

Medical Research and a consulting editor of the Tuberculosis journal. Professor Young helped to establish the Centre for Molecular Microbiology and Infection (CMMI) at Imperial College in 2001 and coordinated a proposal to the BBSRC/EPSRC which led to formation of the Centre for Integrative Systems Biology at Imperial College (CISBIC) in 2005. Professor Young served as the Chairman of the WHO Steering Group on Immunology of Mycobacteria (IMMYC) from 1992 to 1998, and as member of the Coordinating Board and Chair of the Vaccines Working Group of the Global Partnership to Stop TB from 2002 to 2006. He is Principal Investigator on a $20m project funded jointly by the Bill & Melinda Gates Foundation and the Wellcome Trust to address Grand Challenges in Global Health. He also coordinates an international project to study the economic and health impact of bovine tuberculosis in Ethiopia as part of the Wellcome Trust initiative on Animal Health in the Developing World.

Rachel Lai is a Career Development Fellow at the National Institute for Medical Research. Dr. Lai holds a PhD from Imperial College on Clinical Medical Research and was involved in preclinical HIV vaccine development funded by the Bill and Melinda Gates Foundation and the National Institute of Health HIV Vaccine Research and Design (HIVRAD) Program from 2005-2009.

Appendix 1 Study Flow Chart (PTO as continued on next page)

	Visit ABC1<br>Baseline	Visit A1<br>pre-ART PK[3]	Visit ABC2<br>2month f/u	Visit A2<br>Post-ART PK[6,7]	Visit BC1<br>ADR PK [6]	Visit AE<br>5montl
Days post commencing ATT<br>Days post commencing ART	1-3	11-17<br>-1	57-63	57-63<br>42-48	59-65	147-15
Informed consent	X	X			X	
Demographic data	X					
Contact details	X		X			X
Medical history	X		X			X
Height and weight measurement	X	X		X	X	
Physical examination	X		X			X
Concomitant medications check	X	X		X	X	
Urine pregnancy test (women of child bearing age)	X	X		X	X	
Urine drug screen		X		X	X	
Induced or spontaneous sputum[1]	X (3 pots)		X (3 pots)			X(3 pot
Chest x-ray	X					
Clinical chemistry[2]	X	X		X	X	
CD4 lymphocyte count + HIV VL[3]	X			X		
Blood samples for biobank[4]	X<br>2x 3ml serum samples (gold top), 1x3ml plasma (green top) + 1 blue top tube		X<br>1x 3ml serum sample (gold top) and 1x3ml plasma (green top)			X<br>2x 3ml<br>sample<br>top) an<br>1x3ml p<br>(green
	Visit ABC1<br>Baseline	Visit A1<br>pre-ART PK3	Visit ABC2<br>2month f/u	Visit A2<br>Post-ART PK6,7	Visit BC1<br>ADR PK 6	Visit AE<br>5montl
Adherence check including urine for acetyl isoniazid [5]		X				(X)-urir<br>taken c<br>not tak<br>ABC2
8 hour rifampicin, isoniazid and pyrazinamide pharmacokinetics		X		X	X	

[1]On visit ABC1, sputum samples sent for 1) smear grading 2) liquid mycobacterial culture 3)cultures on LJ media for DNA extraction

NOTE: GeneXpert result will be from patient's clinic folder i.e. done by the TB clinic.

NOTE: Drug sensitivity testing will be done using BACTEC 960 and MTBDRplus at a later date

On visit ABC2 and ABC3, samples sent for 1) MTBDRplus on sputum 2)liquid

mycobacterial culture+ drug sensitivity testing 3) MTBDRplus on culture isolates 4) cultures on LJ media for DNA extraction 5) biobank

[2]Serum albumin for visit A1, A2, BC1 and BC2. A sample will be stored (not sent) on visit ABC1

[3]CD4 and HIV VL will be done if not already done by the clinic in the past 1 month. On visit A2, a serum sample will be stored for archived HIV viral load at later date if appropriate

[4]Future work may include host pharmacogenomics, cytokine profiling (serum) and measurement of serum vitamin D.

[5] On visit ABC2 and ABC3 adherence will be assessed via review of TB treatment cards, self reporting, returned blister packs. Urinary isoniazid metabolites will be measured on visit ABC2. Only if urine sample not taken on visit ABC2, sample must be taken on visit ABC3

[6]All patients must be fasted at least 8 hours

[7]Visit ABC2 and A2 will be combined for group A

* ADR Acquired drug resistance. Rifampicin resistance on follow up visits as indicated by GeneXpert will be taken as a surrogate measure of acquired drug resistance. This is applicable to patients who has baseline rifampicin sensitive MTB

Appendix 2 Budget summary

Materials and consumables	No. of samples	cost/sample (ZAR)	cost/sample (£)	Subtotal(£)
GeneXpert	641	231	19.25	12339.25
TB culture +ves	770	100	8.33	6416.67
TB culture -ves	391	80	6.67	2606.67
Bactec 960 RIF/INH DST	778	319.24	26.60	20697.39
Proportions DST for second line drugs	107	396	33.00	3531.00
MTBDRplus	778	168.78	14.07	10942.57
smear	500	23.4	1.95	975.00
urine acetylINH	406	140	11.67	4736.67
CXR	100	240	20.00	2000.00
HIV VL	332	300	25.00	8300.00
CD4	200	75	6.25	1250.00
RIF levels for pre and post ART substudy	1050	250	20.83	7291.67
RIF levels for ADR cases + controls	595	250	20.83	12,395.83
INH levels for ADR cases + controls	595	250	20.83	12,395.83
Z levels for ADR cases + controls	595	250	20.83	12,395.83
chem pathology (albumin only)	214	34.84	2.90	621.31
DNA extraction	75	120	10.00	750.00
Sequencing	75	1200	100.00	7500.00
venesection/cannulation/blood bottles	1	8400	700.00	700.00
nebuliser attachments/sputum pots	1	2400	200.00	200.00
N95 masks	1	3000	250.00	250.00
Patient remuneration (NOT consumable)	1	60000	5000.00	5000.00
TOTAL				**£128,295.67**

References

(1) World Health Organization. WHO report 2011 Global Tuberculosis control. 2011; Available at: http://www.who.int/tb/publications/global_report/2011/gtbr11_main.pdf. Accessed march/28th, 2012.

(2) Day C, Barron P, Massyn N, Padarath A, English R. District Health Barometer 2010/11. Available at: http://www.hst.org.za/publications/district-health-barometer-201011. Accessed 07/19, 2012.

(3) Mac Kenzie WR, Heilig CM, Bozeman L, Johnson JL, Muzanye G, Dunbar D, et al. Geographic differences in time to culture conversion in liquid media: Tuberculosis Trials Consortium study 28. Culture conversion is delayed in Africa. PLoS One 2011 Apr 11;6(4):e18358.

(4) Visser ME, Stead MC, Walzl G, Warren R, Schomaker M, Grewal HM, et al. Baseline predictors of sputum culture conversion in pulmonary tuberculosis: importance of cavities, smoking, time to detection and W-Beijing genotype. PLoS One 2012;7(1):e29588.

(5) Hesseling AC, Walzl G, Enarson DA, Carroll NM, Duncan K, Lukey PT, et al. Baseline sputum time to detection predicts month two culture conversion and relapse in non-HIV-infected patients. Int J Tuberc Lung Dis 2010 May;14(5):560-570.

(6) World Health Organization. Multidrug and extensively drug-resistant TB (M/XDR-TB) 2010 Global report on surveillance and response. Available at: http://whqlibdoc.who.int/publications/2010/9789241599191_eng.pdf. Accessed 07/19, 2012.

(7) World Health Organization. The Global Task Force on XDR-TB. Update February 2007 Stop TB Department 2007; Available at: http://www.stoptb.org/events/world_tb_day/2007/assets/documents/globaltaskforce_update_feb07.pdf. Accessed July 26, 2012.

(8) Laserson KF, Thorpe LE, Leimane V, Weyer K, Mitnick CD, Riekstina V, et al. Speaking the same language: treatment outcome definitions for multidrug-resistant tuberculosis. Int J Tuberc Lung Dis 2005 Jun;9(6):640-645.

(9) Torun T, Gungor G, Ozmen I, Bolukbasi Y, Maden E, Bicakci B, et al. Side effects associated with the treatment of multidrug-resistant tuberculosis. Int J Tuberc Lung Dis 2005 Dec;9(12):1373-1377.

(10) Nathanson E, Gupta R, Huamani P, Leimane V, Pasechnikov AD, Tupasi TE, et al. Adverse events in the treatment of multidrug-resistant tuberculosis: results from the DOTS-Plus initiative. Int J Tuberc Lung Dis 2004 Nov;8(11):1382-1384.

(11) Shin SS, Pasechnikov AD, Gelmanova IY, Peremitin GG, Strelis AK, Mishustin S, et al. Adverse reactions among patients being treated for MDR-TB in Tomsk, Russia. Int J Tuberc Lung Dis 2007 Dec;11(12):1314-1320.

(12) Carroll MW, Lee M, Cai Y, Hallahan CW, Shaw PA, Min JH, et al. Frequency of adverse reactions to first- and second-line anti-tuberculosis chemotherapy in a Korean cohort. Int J Tuberc Lung Dis 2012 Jul;16(7):961-966.

(13) Isaakidis P, Varghese B, Mansoor H, Cox HS, Ladomirska J, Saranchuk P, et al. Adverse Events among HIV/MDR-TB Co-Infected Patients Receiving Antiretroviral and Second Line Anti-TB Treatment in Mumbai, India. PLoS One 2012;7(7):e40781.

(14) World Health Organization. 2011/2012 Tuberculosis Global Facts. 2011; Available at: http://www.who.int/tb/publications/2011/factsheet_tb_2011.pdf.

(15) Nathanson E, Lambregts-van Weezenbeek C, Rich ML, Gupta R, Bayona J, Blondal K, et al. Multidrug-resistant tuberculosis management in resource-limited settings. Emerg Infect Dis 2006 Sep;12(9):1389-1397.

(16) Palacios E, Franke M, Munoz M, Hurtado R, Dallman R, Chalco K, et al. HIV-positive patients treated for multidrug-resistant tuberculosis: clinical outcomes in the HAART era. Int J Tuberc Lung Dis 2012 Mar;16(3):348-354.

(17) Gandhi NR, Shah NS, Andrews JR, Vella V, Moll AP, Scott M, et al. HIV coinfection in multidrug- and extensively drug-resistant tuberculosis results in high early mortality. Am J Respir Crit Care Med 2010 Jan 1;181(1):80-86.

(18) Brust JC, Gandhi NR, Carrara H, Osburn G, Padayatchi N. High treatment failure and default rates for patients with multidrug-resistant tuberculosis in KwaZulu-Natal, South Africa, 2000-2003. Int J Tuberc Lung Dis 2010 Apr;14(4):413-419.

(19) Farley JE, Ram M, Pan W, Waldman S, Cassell GH, Chaisson RE, et al. Outcomes of multi-drug resistant tuberculosis (MDR-TB) among a cohort of South African patients with high HIV prevalence. PLoS One 2011;6(7):e20436.

(20) Russell DG, Barry CE,3rd, Flynn JL. Tuberculosis: what we don't know can, and does, hurt us. Science 2010 May 14;328(5980):852-856.

(21) Pasipanodya JG, Gumbo T. A new evolutionary and pharmacokinetic-pharmacodynamic scenario for rapid emergence of resistance to single and multiple anti-tuberculosis drugs. Curr Opin Pharmacol 2011 Oct;11(5):457-463.

(22) World Health Organisation. Treatment of tuberculosis guidelines. Fourth edition. 2010; Available at: http://whqlibdoc.who.int/publications/2010/9789241547833_eng.pdf. Accessed July/30, 2010.

(23) Mitchison DA. The Garrod Lecture. Understanding the chemotherapy of tuberculosis--current problems. J Antimicrob Chemother 1992 May;29(5):477-493.

(24) Mitchison DA. Role of individual drugs in the chemotherapy of tuberculosis. Int J Tuberc Lung Dis 2000 Sep;4(9):796-806.

(25) Gumbo T, Louie A, Deziel MR, Liu W, Parsons LM, Salfinger M, et al. Concentration-dependent Mycobacterium tuberculosis killing and prevention of resistance by rifampin. Antimicrob Agents Chemother 2007 Nov;51(11):3781-3788.

(26) Heifets L, Lindholm-Levy P. Pyrazinamide sterilizing activity in vitro against semidormant Mycobacterium tuberculosis bacterial populations. Am Rev Respir Dis 1992 May;145(5):1223-1225.

(27) Nuermberger E, Grosset J. Pharmacokinetic and pharmacodynamic issues in the treatment of mycobacterial infections. Eur J Clin Microbiol Infect Dis 2004 Apr;23(4):243-255.

(28) Edginton ME, Sekatane CS, Goldstein SJ. Patients' beliefs: do they affect tuberculosis control? A study in a rural district of South Africa. Int J Tuberc Lung Dis 2002 Dec;6(12):1075-1082.

(29) Johnston JC, Shahidi NC, Sadatsafavi M, Fitzgerald JM. Treatment outcomes of multidrug-resistant tuberculosis: a systematic review and meta-analysis. PLoS One 2009 Sep 9;4(9):e6914.

(30) Kurbatova EV, Taylor A, Gammino VM, Bayona J, Becerra M, Danilovitz M, et al. Predictors of poor outcomes among patients treated for multidrug-resistant tuberculosis at DOTS-plus projects. Tuberculosis (Edinb) 2012 Jul 10.

(31) Iseman MD. Treatment of multidrug-resistant tuberculosis. N Engl J Med 1993 Sep 9;329(11):784-791.

(32) Temple B, Ayakaka I, Ogwang S, Nabanjja H, Kayes S, Nakubulwa S, et al. Rate and amplification of drug resistance among previously-treated patients with tuberculosis in Kampala, Uganda. Clin Infect Dis 2008 Nov 1;47(9):1126-1134.

(33) Pepper DJ, Rebe K, Morroni C, Wilkinson RJ, Meintjes G. Clinical deterioration during antitubercular treatment at a district hospital in South Africa: the importance of drug resistance and AIDS defining illnesses. PLoS One 2009;4(2):e4520.

(34) Seung KJ, Gelmanova IE, Peremitin GG, Golubchikova VT, Pavlova VE, Sirotkina OB, et al. The effect of initial drug resistance on treatment response and acquired drug resistance during standardized short-course chemotherapy for tuberculosis. Clin Infect Dis 2004 Nov 1;39(9):1321-1328.

(35) Yoshiyama T, Yanai H, Rhiengtong D, Palittapongarnpim P, Nampaisan O, Supawitkul S, et al. Development of acquired drug resistance in recurrent tuberculosis patients with various previous treatment outcomes. Int J Tuberc Lung Dis 2004 Jan;8(1):31-38.

(36) Cox HS, Niemann S, Ismailov G, Doshetov D, Orozco JD, Blok L, et al. Risk of acquired drug resistance during short-course directly observed treatment of tuberculosis in an area with high levels of drug resistance. Clin Infect Dis 2007 Jun 1;44(11):1421-1427.

(37) Bonnet M, Pardini M, Meacci F, Orrù G, Yesilkaya H, Jarosz T, et al. **Treatment of tuberculosis in a region with high drug resistance: outcomes, drug resistance amplification and re-infection** . PloS one 2011;6(8):e23081.

(38) Buu TN, Huyen MN, van Soolingen D, Lan NT, Quy HT, Tiemersma EW, et al. The Mycobacterium tuberculosis Beijing genotype does not affect tuberculosis treatment failure in Vietnam. Clin Infect Dis 2010 Oct 15;51(8):879-886.

(39) Quy HT, Lan NT, Borgdorff MW, Grosset J, Linh PD, Tung LB, et al. Drug resistance among failure and relapse cases of tuberculosis: is the standard re-treatment regimen adequate? Int J Tuberc Lung Dis 2003 Jul;7(7):631-636.

(40) Porco TC, Oh P, Flood JM. Anti-tuberculosis drug resistance acquired during treatment: an analysis of cases reported in California, 1994-2006. Clin Infect Dis 2012 Dec 7.

(41) Gandhi NR, Moll A, Sturm AW, Pawinski R, Govender T, Lalloo U, et al. Extensively drug-resistant tuberculosis as a cause of death in patients co-infected with tuberculosis and HIV in a rural area of South Africa. Lancet 2006 Nov 4;368(9547):1575-1580.

(42) Moodley P, Shah NS, Tayob N, Connolly C, Zetola N, Gandhi N, et al. Spread of extensively drug-resistant tuberculosis in KwaZulu-Natal province, South Africa. PLoS One 2011;6(5):e17513.

(43) Borrell S, Gagneux S. Strain diversity, epistasis and the evolution of drug resistance in Mycobacterium tuberculosis. Clin Microbiol Infect 2011 06;17(1469-0691; 1198-743; 6):815-820.

(44) Gagneux S, Long CD, Small PM, Van T, Schoolnik GK, Bohannan BJ. The competitive cost of antibiotic resistance in Mycobacterium tuberculosis. Science 2006 Jun 30;312(5782):1944-1946.

(45) Borrell S, Gagneux S. Infectiousness, reproductive fitness and evolution of drug-resistant Mycobacterium tuberculosis. Int J Tuberc Lung Dis 2009 Dec;13(12):1456-1466.

(46) Fenner L, Egger M, Bodmer T, Altpeter E, Zwahlen M, Jaton K, et al. Effect of mutation and genetic background on drug resistance in Mycobacterium tuberculosis. Antimicrob Agents Chemother 2012 Jun;56(6):3047-3053.

(47) Comas I, Borrell S, Roetzer A, Rose G, Malla B, Kato-Maeda M, et al. Whole-genome sequencing of rifampicin-resistant Mycobacterium tuberculosis strains identifies compensatory mutations in RNA polymerase genes. Nat Genet 2011 Dec 18;44(1):106-110.

(48) de Vos M, Muller B, Borrell S, Black PA, van Helden PD, Warren RM, et al. Putative compensatory mutations in the rpoC gene of rifampin-resistant Mycobacterium tuberculosis are associated with ongoing transmission. Antimicrob Agents Chemother 2013 Feb;57(2):827-832.

(49) Middelkoop K, Bekker LG, Mathema B, Myer L, Shashkina E, Whitelaw A, et al. Factors affecting tuberculosis strain success over 10 years in a high TB- and HIV-burdened community. Int J Epidemiol 2014 Aug;43(4):1114-1122.

(50) Middelkoop K, Bekker LG, Mathema B, Shashkina E, Kurepina N, Whitelaw A, et al. Molecular epidemiology of Mycobacterium tuberculosis in a South African community with high HIV prevalence. J Infect Dis 2009 Oct 15;200(8):1207-1211.

(51) Cowley D, Govender D, February B, Wolfe M, Steyn L, Evans J, et al. Recent and rapid emergence of W-Beijing strains of Mycobacterium tuberculosis in Cape Town, South Africa. Clin Infect Dis 2008 11/15;47(1537-6591; 1058-4838; 10):1252-1259.

(52) Muller B, Borrell S, Rose G, Gagneux S. The heterogeneous evolution of multidrug-resistant Mycobacterium tuberculosis. Trends Genet 2013 Mar;29(3):160-169.

(53) Mehta JB, Shantaveerapa H, Byrd RP,Jr, Morton SE, Fountain F, Roy TM. Utility of rifampin blood levels in the treatment and follow-up of active pulmonary tuberculosis in patients who were slow to respond to routine directly observed therapy. Chest 2001 Nov;120(5):1520-1524.

(54) Weiner M, Burman W, Vernon A, Benator D, Peloquin CA, Khan A, et al. Low isoniazid concentrations and outcome of tuberculosis treatment with once-weekly isoniazid and rifapentine. Am J Respir Crit Care Med 2003 May 15;167(10):1341-1347.

(55) Chideya S, Winston CA, Peloquin CA, Bradford WZ, Hopewell PC, Wells CD, et al. Isoniazid, rifampin, ethambutol, and pyrazinamide pharmacokinetics and treatment outcomes among a predominantly HIV-infected cohort of adults with tuberculosis from Botswana. Clin Infect Dis 2009 Jun 15;48(12):1685-1694.

(56) Pasipanodya JG, Srivastava S, Gumbo T. Meta-analysis of clinical studies supports the pharmacokinetic variability hypothesis for acquired drug resistance and failure of antituberculosis therapy. Clin Infect Dis 2012 Jul;55(2):169-177.

(57) Mitchison DA. Pharmacokinetic/pharmacodynamic parameters and the choice of high-dosage rifamycins. Int J Tuberc Lung Dis 2012 Sep;16(9):1186-1189.

(58) Jayaram R, Gaonkar S, Kaur P, Suresh BL, Mahesh BN, Jayashree R, et al. Pharmacokinetics-pharmacodynamics of rifampin in an aerosol infection model of tuberculosis. Antimicrob Agents Chemother 2003 Jul;47(7):2118-2124.

(59) Siddiqi N, Das R, Pathak N, Banerjee S, Ahmed N, Katoch VM, et al. Mycobacterium tuberculosis isolate with a distinct genomic identity overexpresses a tap-like efflux pump. Infection 2004 Apr;32(2):109-111.

(60) Gumbo T, Louie A, Liu W, Brown D, Ambrose PG, Bhavnani SM, et al. Isoniazid bactericidal activity and resistance emergence: integrating pharmacodynamics and pharmacogenomics to predict efficacy in different ethnic populations. Antimicrob Agents Chemother 2007 Jul;51(7):2329-2336.

(61) Srivastava S, Musuka S, Sherman C, Meek C, Leff R, Gumbo T. Efflux-pump-derived multiple drug resistance to ethambutol monotherapy in Mycobacterium tuberculosis and the pharmacokinetics and pharmacodynamics of ethambutol. J Infect Dis 2010 Apr 15;201(8):1225-1231.

(62) Gumbo T, Dona CS, Meek C, Leff R. Pharmacokinetics-pharmacodynamics of pyrazinamide in a novel in vitro model of tuberculosis for sterilizing effect: a paradigm for faster assessment of new antituberculosis drugs. Antimicrob Agents Chemother 2009 Aug;53(8):3197-3204.

(63) Kjellsson MC, Via LE, Goh A, Weiner D, Low KM, Kern S, et al. Pharmacokinetic evaluation of the penetration of antituberculosis agents in rabbit pulmonary lesions. Antimicrob Agents Chemother 2012 Jan;56(1):446-457.

(64) Goutelle S, Bourguignon L, Maire PH, Van Guilder M, Conte JE,Jr, Jelliffe RW. Population modeling and Monte Carlo simulation study of the pharmacokinetics and antituberculosis pharmacodynamics of rifampin in lungs. Antimicrob Agents Chemother 2009 Jul;53(7):2974-2981.

(65) Ziglam HM, Baldwin DR, Daniels I, Andrew JM, Finch RG. Rifampicin concentrations in bronchial mucosa, epithelial lining fluid, alveolar macrophages and serum following a single 600 mg oral dose in patients undergoing fibre-optic bronchoscopy. J Antimicrob Chemother 2002 Dec;50(6):1011-1015.

(66) Wilkins JJ, Savic RM, Karlsson MO, Langdon G, McIlleron H, Pillai G, et al. Population pharmacokinetics of rifampin in pulmonary tuberculosis patients, including a semimechanistic model to describe variable absorption. Antimicrob Agents Chemother 2008 Jun;52(6):2138-2148.

(67) Wilkins JJ, Langdon G, McIlleron H, Pillai G, Smith PJ, Simonsson US. Variability in the population pharmacokinetics of isoniazid in South African tuberculosis patients. Br J Clin Pharmacol 2011 Jul;72(1):51-62.

(68) Wilkins JJ, Langdon G, McIlleron H, Pillai GC, Smith PJ, Simonsson US. Variability in the population pharmacokinetics of pyrazinamide in South African tuberculosis patients. Eur J Clin Pharmacol 2006 Sep;62(9):727-735.

(69) McIlleron H, Rustomjee R, Vahedi M, Mthiyane T, Denti P, Connolly C, et al. Reduced antituberculosis drug concentrations in HIV-infected patients who are men or have low weight: implications for international dosing guidelines. Antimicrob Agents Chemother 2012 Jun;56(6):3232-3238.

(70) McIlleron H, Wash P, Burger A, Norman J, Folb PI, Smith P. Determinants of rifampin, isoniazid, pyrazinamide, and ethambutol pharmacokinetics in a cohort of tuberculosis patients. Antimicrob Agents Chemother 2006 Apr;50(4):1170-1177.

(71) Boman G, Ringberger VA. Binding of rifampicin by human plasma proteins. Eur J Clin Pharmacol 1974 Aug 23;7(5):369-373.

(72) Barroso EC, Pinheiro VG, Facanha MC, Carvalho MR, Moura ME, Campelo CL, et al. Serum concentrations of rifampin, isoniazid, and intestinal absorption, permeability in patients with multidrug resistant tuberculosis. Am J Trop Med Hyg 2009 Aug;81(2):322-329.

(73) Gurumurthy P, Ramachandran G, Hemanth Kumar AK, Rajasekaran S, Padmapriyadarsini C, Swaminathan S, et al. Decreased bioavailability of rifampin and other antituberculosis drugs in patients with advanced human immunodeficiency virus disease. Antimicrob Agents Chemother 2004 Nov;48(11):4473-4475.

(74) Choudhri SH, Hawken M, Gathua S, Minyiri GO, Watkins W, Sahai J, et al. Pharmacokinetics of antimycobacterial drugs in patients with tuberculosis, AIDS, and diarrhea. Clin Infect Dis 1997 Jul;25(1):104-111.

(75) Panchagnula R, Rungta S, Sancheti P, Agrawal S, Kaul CL. In vitro evaluation of food effect on the bioavailability of rifampicin from antituberculosis fixed dose combination formulations. Farmaco 2003 Nov;58(11):1099-1103.

(76) Weiner M, Peloquin C, Burman W, Luo CC, Engle M, Prihoda TJ, et al. Effects of tuberculosis, race, and human gene SLCO1B1 polymorphisms on rifampin concentrations. Antimicrob Agents Chemother 2010 Oct;54(10):4192-4200.

(77) Loktionov A, Moore W, Spencer SP, Vorster H, Nell T, O'Neill IK, et al. Differences in N-acetylation genotypes between Caucasians and Black South Africans: implications for cancer prevention. Cancer Detect Prev 2002;26(1):15-22.

(78) Chigutsa E, Visser ME, Swart EC, Denti P, Pushpakom S, Egan D, et al. The SLCO1B1 rs4149032 polymorphism is highly prevalent in South Africans and is associated with reduced rifampin concentrations: dosing implications. Antimicrob Agents Chemother 2011 Sep;55(9):4122-4127.

(79) Ruslami R, Nijland H, Aarnoutse R, Alisjahbana B, Soeroto AY, Ewalds S, et al. Evaluation of high-versus standard-dose rifampin in Indonesian patients with pulmonary tuberculosis. Antimicrob Agents Chemother 2006 Feb;50(2):822-823.

(80) Esen M, Kremsner PG, Schleucher R, Gassler M, Imoukhuede EB, Imbault N, et al. Safety and immunogenicity of GMZ2 - a MSP3-GLURP fusion protein malaria vaccine candidate. Vaccine 2009 Nov 16;27(49):6862-6868.

(81) van Ingen J, Aarnoutse RE, Donald PR, Diacon AH, Dawson R, Plemper van Balen G, et al. Why Do We Use 600 mg of Rifampicin in Tuberculosis Treatment? Clin Infect Dis 2011 May;52(9):e194-9.

(82) Smythe W, Khandelwal A, Merle C, Rustomjee R, Gninafon M, Bocar Lo M, et al. A semimechanistic pharmacokinetic-enzyme turnover model for rifampin autoinduction in adult tuberculosis patients. Antimicrob Agents Chemother 2012 Apr;56(4):2091-2098.

(83) van Ingen J, Aarnoutse R, de Vries G, Boeree MJ, van Soolingen D. Low-level rifampicin-resistant Mycobacterium tuberculosis strains raise a new therapeutic challenge. Int J Tuberc Lung Dis 2011 Jul;15(7):990-992.

(84) Ruslami R, Nijland HM, Alisjahbana B, Parwati I, van Crevel R, Aarnoutse RE. Pharmacokinetics and tolerability of a higher rifampin dose versus the standard dose in pulmonary tuberculosis patients. Antimicrob Agents Chemother 2007 Jul;51(7):2546-2551.

(85) Diacon AH, Patientia RF, Venter A, van Helden PD, Smith PJ, McIlleron H, et al. Early bactericidal activity of high-dose rifampin in patients with pulmonary tuberculosis evidenced by positive sputum smears. Antimicrob Agents Chemother 2007 Aug;51(8):2994-2996.

(86) Srivastava S, Pasipanodya JG, Meek C, Leff R, Gumbo T. Multidrug-resistant tuberculosis not due to noncompliance but to between-patient pharmacokinetic variability. J Infect Dis 2011 Dec 15;204(12):1951-1959.

(87) Dartois V. Drug forgiveness and interpatient pharmacokinetic variability in tuberculosis. J Infect Dis 2011 Dec 15;204(12):1827-1829.

(88) Srivastava S, Sherman C, Meek C, Leff R, Gumbo T. Pharmacokinetic mismatch does not lead to emergence of isoniazid- or rifampin-resistant Mycobacterium tuberculosis but to better antimicrobial effect: a new paradigm for antituberculosis drug scheduling. Antimicrob Agents Chemother 2011 Nov;55(11):5085-5089.

(89) Louw GE, Warren RM, Gey van Pittius NC, Leon R, Jimenez A, Hernandez-Pando R, et al. Rifampicin reduces susceptibility to ofloxacin in rifampicin-resistant Mycobacterium tuberculosis through efflux. Am J Respir Crit Care Med 2011 Jul 15;184(2):269-276.

(90) Colangeli R, Helb D, Sridharan S, Sun J, Varma-Basil M, Hazbon MH, et al. The Mycobacterium tuberculosis iniA gene is essential for activity of an efflux pump that confers drug tolerance to both isoniazid and ethambutol. Mol Microbiol 2005 Mar;55(6):1829-1840.

(91) Gupta AK, Chauhan DS, Srivastava K, Das R, Batra S, Mittal M, et al. Estimation of efflux mediated multi-drug resistance and its correlation with expression levels of two major efflux pumps in mycobacteria. J Commun Dis 2006 Mar;38(3):246-254.

(92) Waddell SJ, Butcher PD. Microarray analysis of whole genome expression of intracellular Mycobacterium tuberculosis. Curr Mol Med 2007 May;7(3):287-296.

(93) Waddell SJ, Butcher PD, Stoker NG. RNA profiling in host-pathogen interactions. Curr Opin Microbiol 2007 Jun;10(3):297-302.

(94) Liu JM, Camilli A. A broadening world of bacterial small RNAs. Curr Opin Microbiol 2010 Feb;13(1):18-23.

(95) Gottesman S. Micros for microbes: non-coding regulatory RNAs in bacteria. Trends Genet 2005 Jul;21(7):399-404.

(96) Padalon-Brauch G, Hershberg R, Elgrably-Weiss M, Baruch K, Rosenshine I, Margalit H, et al. Small RNAs encoded within genetic islands of Salmonella typhimurium show host-induced expression and role in virulence. Nucleic Acids Res 2008 Apr;36(6):1913-1927.

(97) Romby P, Wagner EG. Exploring the complex world of RNA regulation. Biol Cell 2008 Jan;100(1):e1-3.

(98) Papenfort K, Vogel J. Regulatory RNA in bacterial pathogens. Cell Host Microbe 2010 Jul 22;8(1):116-127.

(99) DiChiara JM, Contreras-Martinez LM, Livny J, Smith D, McDonough KA, Belfort M. Multiple small RNAs identified in Mycobacterium bovis BCG are also expressed in Mycobacterium tuberculosis and Mycobacterium smegmatis. Nucleic Acids Res 2010 Jul;38(12):4067-4078.

(100) Arnvig KB, Young DB. Identification of small RNAs in Mycobacterium tuberculosis. Mol Microbiol 2009 Aug;73(3):397-408.

(101) Arnvig KB, Comas I, Thomson NR, Houghton J, Boshoff HI, Croucher NJ, et al. Sequence-based analysis uncovers an abundance of non-coding RNA in the total transcriptome of Mycobacterium tuberculosis. PLoS Pathog 2011 Nov;7(11):e1002342.

(102) Keren I, Minami S, Rubin E, Lewis K. Characterization and transcriptome analysis of Mycobacterium tuberculosis persisters. MBio 2011 Jun 14;2(3):e00100-11.

(103) McKinney JD, Honer zu Bentrup K, Munoz-Elias EJ, Miczak A, Chen B, Chan WT, et al. Persistence of Mycobacterium tuberculosis in macrophages and mice requires the glyoxylate shunt enzyme isocitrate lyase. Nature 2000 Aug 17;406(6797):735-738.

(104) Fenhalls G, Stevens L, Moses L, Bezuidenhout J, Betts JC, Helden Pv P, et al. In situ detection of Mycobacterium tuberculosis transcripts in human lung granulomas reveals differential gene expression in necrotic lesions. Infect Immun 2002 Nov;70(11):6330-6338.

(105) Garton NJ, Waddell SJ, Sherratt AL, Lee SM, Smith RJ, Senner C, et al. Cytological and transcript analyses reveal fat and lazy persister-like bacilli in tuberculous sputum. PLoS Med 2008 Apr 1;5(4):e75.

(106) Cox HS, McDermid C, Azevedo V, Muller O, Coetzee D, Simpson J, et al. Epidemic levels of drug resistant tuberculosis (MDR and XDR-TB) in a high HIV prevalence setting in Khayelitsha, South Africa. PLoS One 2010 Nov 15;5(11):e13901.

(107) McIlleron H, Meintjes G, Burman WJ, Maartens G. Complications of antiretroviral therapy in patients with tuberculosis: drug interactions, toxicity, and immune reconstitution inflammatory syndrome. J Infect Dis 2007 Aug 15;196 Suppl 1:S63-75.

(108) Weiner M, Benator D, Burman W, Peloquin CA, Khan A, Vernon A, et al. Association between acquired rifamycin resistance and the pharmacokinetics of rifabutin and isoniazid among patients with HIV and tuberculosis. Clin Infect Dis 2005 May 15;40(10):1481-1491.

(109) Li J, Munsiff SS, Driver CR, Sackoff J. Relapse and acquired rifampin resistance in HIV-infected patients with tuberculosis treated with rifampin- or rifabutin-based regimens in New York City, 1997-2000. Clin Infect Dis 2005 Jul 1;41(1):83-91.

(110) Lutfey M, Della-Latta P, Kapur V, Palumbo LA, Gurner D, Stotzky G, et al. Independent origin of mono-rifampin-resistant Mycobacterium tuberculosis in patients with AIDS. Am J Respir Crit Care Med 1996 Feb;153(2):837-840.

(111) Munsiff SS, Joseph S, Ebrahimzadeh A, Frieden TR. Rifampin-monoresistant tuberculosis in New York City, 1993-1994. Clin Infect Dis 1997 Dec;25(6):1465-1467.

(112) Corbett EL, Watt CJ, Walker N, Maher D, Williams BG, Raviglione MC, et al. The growing burden of tuberculosis: global trends and interactions with the HIV epidemic. Arch Intern Med 2003 May 12;163(9):1009-1021.

(113) Patel KB, Belmonte R, Crowe HM. Drug malabsorption and resistant tuberculosis in HIV-infected patients. N Engl J Med 1995 Feb 2;332(5):336-337.

(114) Peloquin CA, Berning SE, Huitt GA, Iseman MD. AIDS and TB drug absorption. Int J Tuberc Lung Dis 1999 Dec;3(12):1143-1144.

(115) Sahai J, Gallicano K, Swick L, Tailor S, Garber G, Seguin I, et al. Reduced plasma concentrations of antituberculosis drugs in patients with HIV infection. Ann Intern Med 1997 Aug 15;127(4):289-293.

(116) Taylor B, Smith PJ. Does AIDS impair the absorption of antituberculosis agents? Int J Tuberc Lung Dis 1998 Aug;2(8):670-675.

(117) Perlman DC, Segal Y, Rosenkranz S, Rainey PM, Remmel RP, Salomon N, et al. The clinical pharmacokinetics of rifampin and ethambutol in HIV-infected persons with tuberculosis. Clin Infect Dis 2005 Dec 1;41(11):1638-1647.

(118) Jonsson S, Davidse A, Wilkins J, Van der Walt JS, Simonsson US, Karlsson MO, et al. Population pharmacokinetics of ethambutol in South African tuberculosis patients. Antimicrob Agents Chemother 2011 Sep;55(9):4230-4237.

(119) Bao Y, Silva TM, Guerrant RL, Lima AM, Fox JW. Direct analysis of mannitol, lactulose and glucose in urine samples by high-performance anion-exchange chromatography with pulse amperometric detection. Clinical evaluation of intestinal permeability in human immunodeficiency virus infection. J Chromatogr B Biomed Appl 1996 Oct 11;685(1):105-112.

(120) Saleri N, Dembele SM, Villani P, Carvalho AC, Cusato M, Bonkoungou V, et al. Systemic exposure to rifampicin in patients with tuberculosis and advanced HIV disease during highly active antiretroviral therapy in Burkina Faso. J Antimicrob Chemother 2012 Feb;67(2):469-472.

(121) Volmink J, Garner P. Directly observed therapy for treating tuberculosis. Cochrane Database Syst Rev 2007 Oct 17;(4)(4):CD003343.

(122) Parrish NM, Dick JD, Bishai WR. Mechanisms of latency in Mycobacterium tuberculosis. Trends Microbiol 1998 Mar;6(3):107-112.
